# Traditional Chinese Medicine Oral Liquids Combined With Azithromycin for *Mycoplasma pneumoniae* Pneumonia in Children: A Bayesian Network Meta-Analysis

**DOI:** 10.3389/fphar.2021.652412

**Published:** 2021-05-28

**Authors:** Zhe Chen, Qingyang Shi, Yingying Peng, Yongjie Chen, Lujia Cao, Bo Pang, Zhaochen Ji, Chunxiang Liu, Junhua Zhang

**Affiliations:** ^1^ Evidence-Based Medicine Center, Tianjin University of Traditional Chinese Medicine, Tianjin, China; ^2^ Department of Pediatrics, First Teaching Hospital of Tianjin University of Traditional Chinese Medicine, Tianjin, China; ^3^ National Clinical Research Center for Chinese Medicine Acupuncture and Moxibustion, Tianjin, China; ^4^ Department of Epidemiology and Statistic, School of Public Health, Tianjin Medical University, Tianjin, China

**Keywords:** *Mycoplasma pneumoniae* pneumonia in children, traditional Chinese medicine oral liquids, Azithromycin, network meta-analysis, randomized controlled trial

## Abstract

**Background:** *Mycoplasma pneumoniae* pneumonia (MPP) causes flu-like symptoms in children, increasing the burden on the health and education systems. In China, traditional Chinese medicine oral liquids (TCMOLs) combined with azithromycin (TCMOLs + Azithromycin) is commonly used to treat MPP in children. However, TCMOLs with the optimal clinical applicability remain unknown. Here, we evaluated the clinical effectiveness and safety of TCMOLs + Azithromycin in children with MPP.

**Methods:** We searched PubMed, Embase, Cochrane Library, Ovid, Web of Science, China National Knowledge Infrastructure (CNKI), Wanfang Data Knowledge Service Platform, and VIP information resource integration service platform databases for eligible randomized controlled trials (RCTs) published from database inception to October 2020. Two reviewers independently performed data extraction and risk of bias assessment. After Bayesian random effect modeling and surface under the cumulative ranking curve (SUCRA) scoring, we ranked each intervention. We assessed heterogeneity using multivariate meta-regression for potential modifiers and used the Grading of Recommendations, Assessment, Development, and Evaluation to rate pooled evidence’s certainty.

**Results:** In the 63 included RCTs with 6,410 children, five different TCMOLs were combined with azithromycin. TCMOLs + Azithromycin had significantly better primary outcomes than did azithromycin alone. Of all TCMOLs, Xiaoer Xiaoji Zhike (XEXJZK)+Azithromycin showed the best effectiveness with respect to the response rate (odds ratio [OR] = 6.5, 95% credible interval [CrI] = 4.3–10; low certainty) and pulmonary rales disappearance time (mean difference [MD] = −2.1, 95% CrI: −2.9 to −1.2; low certainty) with SUCRA 85 and 80%, respectively. Pudilan Xiaoyan + Azithromycin showed the highest effectiveness with respect to cough disappearance time (MD = −2.6, 95% CrI: −3.4 to −1.7; very low certainty) and fever disappearance time (MD = −1.8, 95% CrI: −2.3 to −1.3; very low certainty) with SUCRA 87 and 87%, respectively. The difference in the adverse effects between TCMOLs + Azithromycin and azithromycin alone was nonsignificant.

**Conclusion:** Of the different TCMOLs, XEXJZK may be the best option to combine with azithromycin to treat children with MPP. However, our results should be interpreted with caution due to the low certainty of evidence. In general, TCMOLs’ safety remains unclear because of a lack of evidence. More high-quality RCTs are needed to further evaluate efficacy and safety of these TCMOLs.

## Introduction

*Mycoplasma pneumoniae* (MP) is a main causative agent underlying lower respiratory tract infections in the children and adolescents, at regional and epidemic levels globally (Principi et al., 2001; Jain et al., 2015; Kutty et al., 2019). Among children and adolescents with community-acquired pneumonia, MP pneumonia (MPP) is noted in 10–40% of cases, all at an annually increasing incidence (Korppi et al., 2004; Biondi et al., 2014; Jain et al., 2015). When prolonged course or aggravated infection occurs, MPP affects the nervous, digestive, and cardiovascular system and results in extrapulmonary manifestations (Narita, 2009; Narita, 2010; Flateau et al., 2013; Langley et al., 2016). Although MPP is usually a benign self-limiting disease with a mild and moderate degree of severity, it may lead to severe infection and warrant hospitalization.

MP is a small self-replicating bacterium, which lacks a cell wall and has a very small genome (Razin et al., 1998). Due to the biological structure of MP, antibiotics that act on the MP ribosome and inhibit protein synthesis are used for clinical therapy (Waites et al., 2009). Macrolides, tetracyclines, and fluoroquinolones are considered highly effective against MP infection. Of these, macrolides are the firstline antibiotic used in pediatric MPP treatment (Li et al., 2009).

Macrolide therapy administered in the early stages of MPP shortens the hospitalization and symptom duration (Shah et al., 2012; Lee et al., 2018). Of these macrolides, azithromycin is the firstline medication demonstrates good tolerance and compliance (Shan et al., 2017; Wang and Yang, 2018). Although short-term azithromycin use is effective and safe, its long-term use may lead to drug resistance and increased the incidence of adverse events (AEs) of skin, respiratory, digestive, and nervous system (Smith et al., 2015; Yang et al., 2018). Moreover, the increasing use of azithromycin in Asia, Europe, and North America has resulted in increased MP drug resistance—representing a new challenge (Pereyre et al., 2016). No high-quality evidence on the long-term use of antibiotics for treating MPP in children has been reported thus far (Gardiner et al., 2015), hinting at the potential utility of the alternative medicine.

As traditional Chinese medicine (TCM) continues to evolve, its usefulness in MPP management and treatment are being studied (Liu et al., 2010; Yang et al., 2017). TCM acts through “multiple prescriptions, multiple targets, and multiple pathways” (Sun et al., 2020); hence, it can not only alleviate MPP symptoms but also negate azithromycin drug resistance and prevent the associated AEs to a certain extent (Duan et al., 2019).

For treating upper respiratory tract infections in children, Chinese physicians incline to use combination therapy with TCM (Shi et al., 2019). The TCM oral liquids (TCMOLs) have been approved by China’s National Medical Products Administration for use in children with MPP. A real-world study in a Chinese hospital reported that the combination therapy of TCMOLs and antibiotics is used widely in China (Ren et al., 2016). TCMOLs have been used not only for MPP but also for other acute upper respiratory infections (Gao et al., 2015). In addition, TCMOLs may also have anti-inflammatory, immunoregulation, and IgM response–suppressive effects (Guo et al., 2018; Shi et al., 2020). Although some studies have reported on the effects of TCMOLs, the TCMOL with highest efficacy against MPP in children has not been identified. Therefore, here, we conducted a network meta-analysis to assess the efficacy and safety of the different TCMOLs in combination with azithromycin.

## Methods

We followed the Preferred Reporting Items for Systematic Reviews and Meta-Analyses (PRISMA) and the PRISMA-extension for network meta-analysis to report our results (Moher et al., 2009; Hutton et al., 2015; Shamseer et al., 2015). Our protocol is also registered in PROSPERO (number: CRD42020197026).

### Eligibility Criteria

#### Types of Studies

We only included randomized controlled trials (RCTs), without restrictions on their language and publication type.

#### Types of Participants

We included the studies on ≤15-year-old patients with a clinical diagnosis of MPP. No restriction on the patients’ sex, disease course, geography, and race were implemented. However, only the studies in which the clinical diagnostic criteria followed clinical guidelines or expert consensus were considered (Jiang et al., 2014; Ni, 2019; Lu and Zhang 2020).

#### Types of Interventions

We only considered combinations of various TCMOLs with azithromycin as well as azithromycin alone. As mentioned, the use of TCMOLs is approved by the National Medical Products Administration and can be looked found on the Chinese government’s website. In all studies, the TCMOL administration mode was oral, but no restriction was placed on azithromycin administration mode, dose, and duration.

#### Types of Outcomes

##### Primary Outcomes

1. Response rate. We calculated response rate as [(number of total patients−number of invalid patients)/number of total patients] × 100%. Patients with unchanged or worsening symptoms (e.g., fever, cough, pulmonary rales, and pulmonary shadows in x-ray) were considered invalid. The timeframe of response rate was 2 weeks (14 days).
2. Cough disappearance time. The disappearance of cough was defined as the comprehensive score of day cough symptom score + night cough symptom score being ≤1 for at least 24 h (Lai, 2016). Cough disappearance time was defined as the time interval between treatment initiation and cough disappearance.
3. Fever disappearance time. The disappearance of fever was defined as three or more consecutive measurements of normal body temperature (36–37.3°C) taken 7 h apart maintained over at least 24 h. Fever disappearance time was defined as the time interval between treatment initiation and fever disappearance.
4. Pulmonary rales disappearance time. The disappearance of pulmonary rales was defined as no pulmonary rales being found over at least 24 h. Pulmonary rales disappearance time was defined as the time interval between treatment initiation and pulmonary rales disappearance.

##### Secondary Outcomes

1. Related clinical indicators: Length of hospitalization and disappearance time of pulmonary shadows in x-ray.
2. Inflammatory cytokine: Changes from baseline of absolute tumor necrosis factor (TNF) α, absolute interleukin (IL) 6, and absolute C-reaction protein (CRP).
3. Safety: AEs and adverse incidence rates (ARs).

##### Excluded Criteria

We excluded the studies having missing outcomes, having obvious errors, or having no full-text articles available. We also excluded studies on patients with the clinical diagnosis of non-MPP (e.g., severe MPP or refractory MPP) and the studies comparing the effects of non-azithromycin (e.g., clarithromycin, erythromycin, roxithromycin, or combinations of azithromycin and other non-azithromycin antibiotics). Studies on patients with other organ disorder or serious diseases were also excluded.

### Search Strategy

We searched China National Knowledge Infrastructure (CNKI), Wanfang Data Knowledge Service Platform, VIP information resource integration service platform, PubMed, Embase, Cochrane Library, Ovid, and Web of Science databases for randomized controlled studies on the use of different TCMOLs combined with azithromycin in children with MPP—published from database inception to October 2020. The search was performed using a combination of MeSH terms and free words. The complete search strategy is given in Supplementary File S1. We also searched the reference list of the included studies and clinical trial registries to find more relevant RCTs (Supplementary File S1).

### Literature Screening and Data Extraction

Two researchers (ZC and ZJ) screened and filtered the candidate articles independently according to the criteria. First, they screened the titles and abstracts, and then, they read the full-text to make a final decision. We set a uniform standard to less the measurement bias for data extraction. The extracted information was as follows:

1) Study characteristics: authors, publication year and country.
2) Basic information of the included populations: total number of patients, treatment duration, sex, age, and illness course.
3) Details in the importance of interventions: medication names and usage, intervention types, and comparison types.
4) Information and definitions of outcomes: response rate, fever disappearance time, cough disappearance time, pulmonary rales disappearance time, and number of any AEs.

If there was disagreement among the reviewers, a third researcher (CL) made the final decision on the inclusion of the study.

### Risk of Bias Assessment

Two researchers (LC and YP) independently used the Cochrane risk of bias tool to evaluate the included RCTs’ quality (Higgins et al., 2011). Seven terms were included in the evaluation: random sequence generation, allocation concealment, blinding of participants and personnel, blinding of outcome assessment, incomplete outcome data, selective reporting, and other bias. For each term, a judgment was formed with high, unclear, and low risk of bias. The study with all low risk of bias across the terms was labeled as having overall low risk of bias. The study with at least one high risk of bias across the terms was labeled as having overall high risk of bias. Others were labeled as having overall some concerns risk of bias. In case a disagreement occurred here, a third researcher resolved it through discussion or consultation.

### Statistical Analysis

We measured dichotomous outcomes (response rate and AR) as odds ratios (ORs) and continuous outcomes (fever, cough, pulmonary rales, and pulmonary shadow disappearance time; hospitalization length; and change in all inflammatory cytokines from baseline) as mean differences (MDs).

We performed a network meta-analysis with combined direct and indirect comparisons using the Bayesian random method on the basis of the consistency assumption. Between-study heterogeneity was set with a vague prior. The MDs and log (OR) values were derived from the posterior distribution of the model. The medians and their corresponding 95% credible intervals (CrIs) were also reported.

The models were optimized using the Markov chain Monte Carlo (MCMC) method with weighted sample size running in four chains. The iterations were set as least 200,000 to obtain model convergence. The convergence was assessed using the Brooks–Gelman–Rubin method with potential scale reduction factor up to 1. The ranking of interventions was evaluated based on the surface under the cumulative ranking curve (SUCRA).

We also conducted univariate meta-regressions to detect potential modifiers. Here, five regressors were considered: male proportion, age, treatment duration, illness course, and drug delivery mode. We used predictive mean matching imputation to address some missing data in the regressors and rated the cumulative evidence using the Grading of Recommendations, Assessment, Development, and Evaluation framework and the expanded version for network meta-analysis (Guyatt et al., 2011; Puhan et al., 2014; Brignardello-Petersen et al., 2018). First, we rated the direct comparisons according to the four domains of risk of bias, heterogeneity, indirectness, and publication bias. The rating of indirect comparisons was derived from the rating of the direct comparisons that contributed to the indirect evidence. The rating of network results was combined from the direct and indirect evidence with further considerations of imprecision and incoherence.

The publication bias was assessed by comparison-adjusted funnel plot with Begg’s rank test.

All estimates were calculated using R (version 3.6.2) along with the MCMC engine JAGS (version 3.4.0). The Cochrane tool RevMan (version 5.3) was used for plotting a risk of bias graph.

## Results

### Literature Review

**FIGURE 1:**
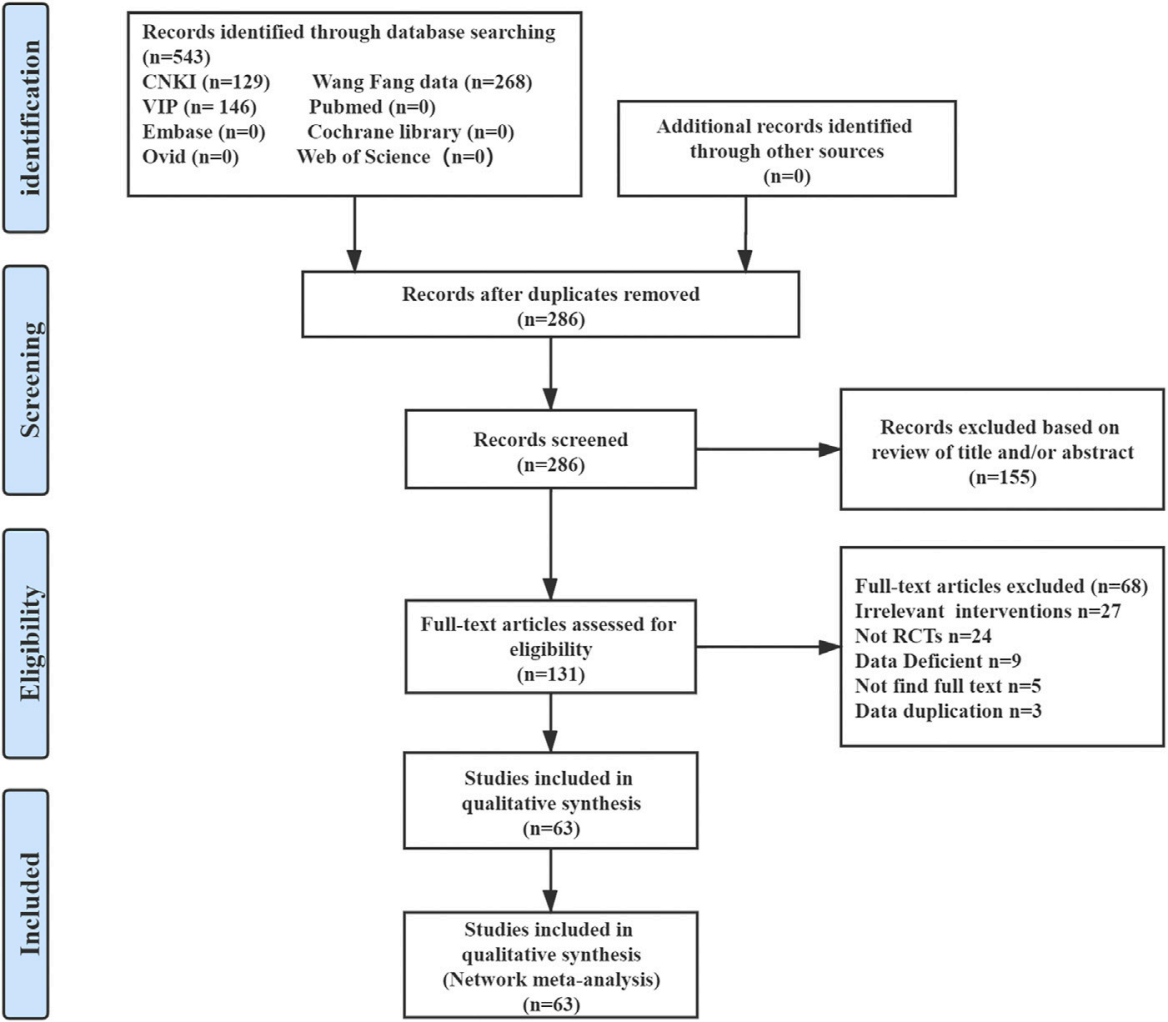
Summary of evidence search and selection.

The database search afforded 543 articles; after screening and removal of duplicates, 286 articles remained. Finally, after screening the titles, abstracts and full-texts, 63 RCTs were included in the final network analysis (Figure 1).

### Studies and Characteristics

The 63 RCTs (Supplementary File S2) included 6,410 children with MPP (*n* = 3,209 [intervention group] and 3,201 [comparison group]). All patients were Chinese, and most of them were infants, toddlers, and preschoolers (median age = 5.84 years)—with median disease course and treatment duration of 5.6 and 13 days, respectively.

Five combined interventions were included: azithromycin alone, with Pudilan Xiaoyan oral liquid (PDLXY; PDLXY + Azithromycin), with Shuanghuanlian oral liquid (SHL; SHL + Azithromycin), with Xiaoer Feire Kechuan oral liquid (XEFRKC; XEFRKC + Azithromycin), or with Xiaoer Xiaoji Zhike oral liquid (XRXJZK; XRXJZK + Azithromycin). Summary of all included studies and detailed chemical characterizations of TCMOLs are listed in Supplementary Tables S1, S2.

Regarding primary outcomes, 93.65, 57.14, 60.32, and 46.03% of the included RCTs reported response rate, cough disappearance time, fever disappearance time, and pulmonary rales disappearance time, respectively. Regarding safety, 46.03% of the included RCTs reported the number of AEs. Complete information is listed in Supplementary Table S3.

### Methodological Quality

All the included RCTs used “random assignment,” and 23 of the RCTs mentioned specific methods, with most of them using the random number tables. In most RCTs, allocation concealment and blinding were not reported: only two RCTs mentioned the blinding of participants and personnel, and only one reported the blinding of outcome assessment. There were no missing data in all included RCTs. Thirty-five of the RCTs did not mention the selective reporting outcomes. Other risks of biases were unclear. The complete evaluation of the risk of bias is presented in Supplementary Figures S1, S2.

## Results of the Network Meta-Analysis

### Primary Outcomes

#### Response Rate

**FIGURE 2:**
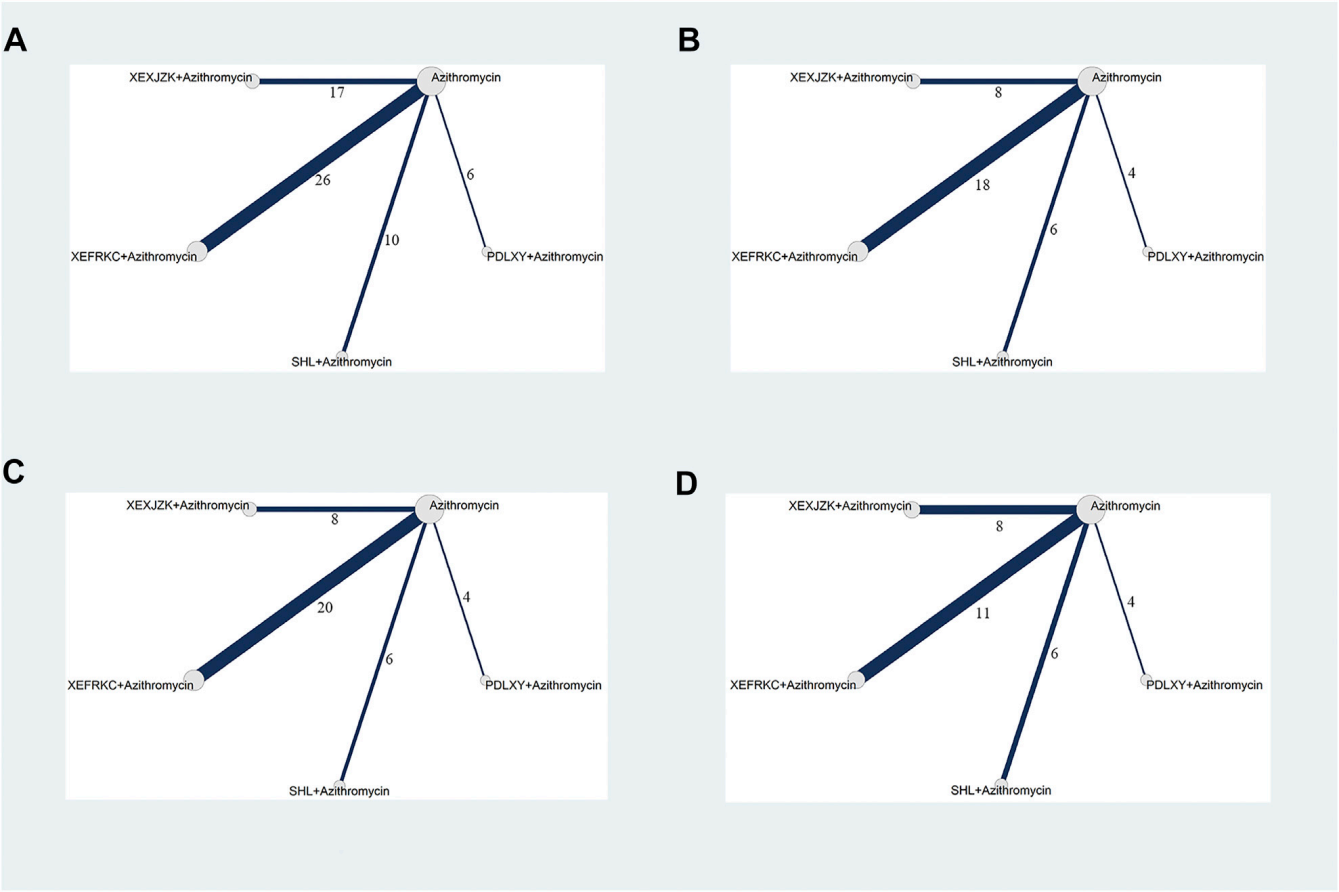
Network Plots of Primary Outcomes. PDLXY: Pudilan Xiaoyan oral liquid; SHL: Shuanghuanlian oral liquid; XEFRKC: Xiaoer Feire Kechuan oral liquid; XRXJZK: Xiaoer Xiaoji Zhike oral liquid. **(A)** response rate; **(B)** cough disappearance time; **(C)** fever disappearance time; **(D)** pulmonary rales disappearance time.

Fifty-nine RCTs reported the response rates for four TCMOLs combined with azithromycin for treating MPP in 5,940 children. The network plot is presented in Figure 2A.

The four interventions demonstrated significant differences compared with azithromycin alone: PDLXY + Azithromycin (OR = 4.3, 95% CrI: 2.3–7.8), SHL + Azithromycin (OR = 5.4, 95% CrI: 3.3–9.2), XEFRKC + Azithromycin (OR = 5.1, 95% CrI: 3.8–7.0), and XEXJZK + Azithromycin (OR = 6.5, 95% CrI: 4.3–10; Table 1 and Supplementary Figure S3A). The network analysis demonstrated no significant differencesw between PDLXY + Azithromycin, SHL + Azithromycin, XEFRKC + Azithromycin, and XEXJZK + Azithromycin outcomes (Table 1).

**TABLE 1:** League table of response rate and cough disappearance time.

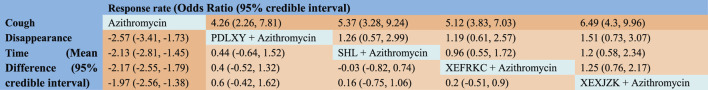

Notes: PDLXY: Pudilan Xiaoyan oral liquid; SHL: Shuanghuanlian oral liquid; XEFRKC: Xiaoer Feire Kechuan oral liquid; XRXJZK: Xiaoer Xiaoji Zhike oral liquid.

Dark brown numbers represent significance, whereas light brown numbers represent no significance.

Regarding the response rate, XEXJZK + Azithromycin had evidence with low certainty of evidence but the best SUCRA value (85%). SHL + Azithromycin, XEFRKC + Azithromycin, and PDLXY + Azithromycin showed low certainty of evidence (SUCRA = 64, 59, and 40%, respectively; Table 2 and Supplementary Figure S4A).

**TABLE 2:** Grading of recommendations, assessment, development, and evaluation of primary outcomes of TCMOLs + Azithromycin compared with azithromycin.

Primary outcomes	Interventions	Relative effect (95% CrI)	Numbers of participants (studies)	Quality of evidence^a^ (GRADE assessment)
Response rate	XEXJZK + A vs. A	OR 6.49 (4.3, 9.96)	1,697 (Seventeen studies)	Low^b^
	XEFRKC + A vs. A	OR 5.12 (3.83, 7.03)	2,543 (Twenty-six studies)	Low^b^
	SHL + A vs. A	OR 5.37 (3.28, 9.24)	1,028 (Ten studies)	Low^b^
	PDLXY + A vs. A	OR 4.26 (2.26, 7.81)	672 (Six studies)	Low^b^
Disappearance time of cough	XEXJZK + A vs. A	MD −1.97 (−2.56, −1.38)	837 (Eight studies)	Low^b^
	XEFRKC + A vs. A	MD −2.17 (−2.55, −1.79)	1,685 (Eighteen studies)	Very low^b^ ^c^ ^f^
	SHL + A vs. A	MD −2.13 (−2.81, −1.45)	520 (Six studies)	Low^b^
	PDLXY + A vs. A	MD −2.57 (−3.41, −1.73)	392 (Four studies)	Low^b^
Disappearance time of fever	XEXJZK + A vs. A	MD −1.74 (−2.1, −1.36)	837 (Eight studies)	Very low^b^ ^c^
	XEFRKC + A vs. A	MD −1.03 (−1.26, −0.79)	1795 (Twenty studies)	Very low^b^ ^c^ ^f^
	SHL + A vs. A	MD −1.27 (−1.71, −0.82)	520 (Six studies)	Very low^b^ ^c^
	PDLXY + A vs. A	MD −1.78 (−2.28, −1.3)	392 (Four studies)	Very low^b^ ^c^
Disappearance time of pulmonary rales	XEXJZK + A vs. A	MD −2.07 (−2.95, −1.2)	837 (Eight studies)	Low^b^
	XEFRKC + A vs. A	MD −1.32 (−2.08, −0.58)	947 (Eleven studies)	Very low^b^ ^c^ ^f^
	SHL + A vs. A	MD −1.73 (−2.73, −0.7)	520 (Six studies)	Very low^b^ ^c^
	PDLXY + A vs. A	MD −1.86 (−3.1, −0.63)	392 (Four studies)	Very low^b^ ^c^

**OR:** Odds Ratio; **MD:** Mean Difference; **95% CrI:** 95% Credible Interval; **GRADE:** Grading of Recommendations, Assessment, Development, and Evaluation; **Interventions:** PDLXY: Pudilan Xiaoyan oral liquid; SHL: Shuanghuanlian oral liquid; XEFRKC: Xiaoer Feire Kechuan oral liquid; XRXJZK: Xiaoer Xiaoji Zhike oral liquid; A: Azithromycin.

a Estimates for primary outcomes with the Grading of Recommendations, Assessment, Development, and Evaluation Assessment.

b Downgraded because of risk of bias.

c Downgraded because of inconsistency.

d Downgraded because of indirectness.

e Downgraded because of imprecision.

f Downgraded because of publication bias.

#### Cough Disappearance Time

Thirty-six RCTs reported cough disappearance time for four TCMOLs combined with azithromycin for treating MPP in 3,434 children. The network plot is presented in Figure 2B.

The four interventions showed significant differences compared with azithromycin alone: PDLXY + Azithromycin (MD = −2.6, 95% CrI: −3.4 to −1.7), SHL + Azithromycin (MD = −2.1, 95% CrI: −2.8 to −1.5), XEFRKC + Azithromycin (MD = −2.2, 95% CrI: −2.6 to −1.8), and XEXJZK + Azithromycin (MD = −2, 95% CrI: −2.6 to −1.4; Table 1 and Supplementary Figure S3B). The network analysis showed no significant differences between PDLXY + Azithromycin, SHL + Azithromycin, XEFRKC + Azithromycin, and XEXJZK + Azithromycin (Table 1).

For cough disappearance time, XEXJZK + Azithromycin showed evidence with low certainty of evidence and a 61% SUCRA. For PDLXY + Azithromycin and SHL + Azithromycin, evidence quality demonstrated low certainty (SUCRA: 87 and 58%, respectively), whereas for XEFRKC + Azithromycin, it demonstrated very low certainty (SUCRA: 44%; Table 2 and Supplementary Figure S4B).

#### Fever Disappearance Time

Thirty-eight RCTs reported fever disappearance time for four TCMOLs combined with azithromycin for treating MPP in 3,544 children. The network plot is presented in Figure 2C.

The four interventions exhibited significant differences compared with azithromycin alone: PDLXY + Azithromycin (MD = −1.8, 95% CrI: −2.3 to −1.3), SHL + Azithromycin (MD = −1.3, 95% CrI: −1.7 to −0.82), XEFRKC + Azithromycin (MD = −1.0, 95% CrI: −1.3 to −0.79), and XEXJZK + Azithromycin (MD = −1.7, 95% CrI: −2.1 to −1.4; Table 3 and Supplementary Figure S3C). The network analysis showed significant differences among PDLXY + Azithromycin, SHL + Azithromycin, and XEXJZK + Azithromycin and between XEZJZK + Azithromycin and PDLXY + Azithromycin compared with XEFRKC + Azithromycin (Table 3).

**TABLE 3:** League table of fever and pulmonary rales disappearance time.

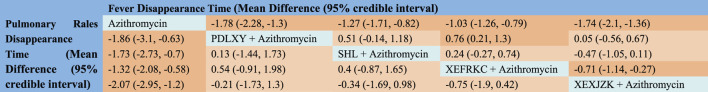

Notes: PDLXY: Pudilan Xiaoyan oral liquid; SHL: Shuanghuanlian oral liquid; XEFRKC: Xiaoer Feire Kechuan oral liquid; XRXJZK: Xiaoer Xiaoji Zhike oral liquid.

Dark brown numbers represent significance, whereas light brown numbers represent no significance.

For fever disappearance time, PDLXY + Azithromycin, XEXJZK + Azithromycin, SHL + Azithromycin, and XEFRKC + Azithromycin showed very low certainty of evidence (SUCRA: 87, 85, 49, and 29%, Respectively; Table 2 and Supplementary Figure S4C).

#### Pulmonary Rales Disappearance Time

Twenty-nine RCTs reported pulmonary rales disappearance time for four TCMOLs combined with azithromycin for treating MPP in 2,696 children. The network plot is presented in Figure 2D.

The four interventions demonstrated significant differences compared with azithromycin alone: PDLXY + Azithromycin (MD = −1.9, 95% CrI: −3.1 to −0.63), SHL + Azithromycin (MD = −1.7, 95% CrI: −2.7 to −0.7), XEFRKC + Azithromycin (MD = −1.3, 95% CrI: −2.1 to −0.58), and XEXJZK + Azithromycin (MD = −2.1, 95% CrI: −2.9 to −1.2; Table 3 and Supplementary Figure S3D). The network analysis indicated no significant differences between PDLXY + Azithromycin, SHL + Azithromycin, XEFRKC + Azithromycin, and XEXJZK + Azithromycin (Table 3).

For pulmonary rales disappearance time, XEXJZK + Azithromycin showed evidence with low certainty but the best SUCRA value (80%). PDLXY + Azithromycin, SHL + Azithromycin, and XEFRKC + Azithromycin showed very low certainty of evidence (SUCRA: 68, 62, and 39%, respectively; Table 2 and Supplementary Figure S4D).

#### Biplot of Primary Outcomes

Based on the SUCRA of the primary outcomes, we use the biplot to combine the SUCRA of response rate with the SUCRAs of cough, fever, and pulmonary rales disappearance times so as to find the best optional interventions.

**FIGURE 3:**
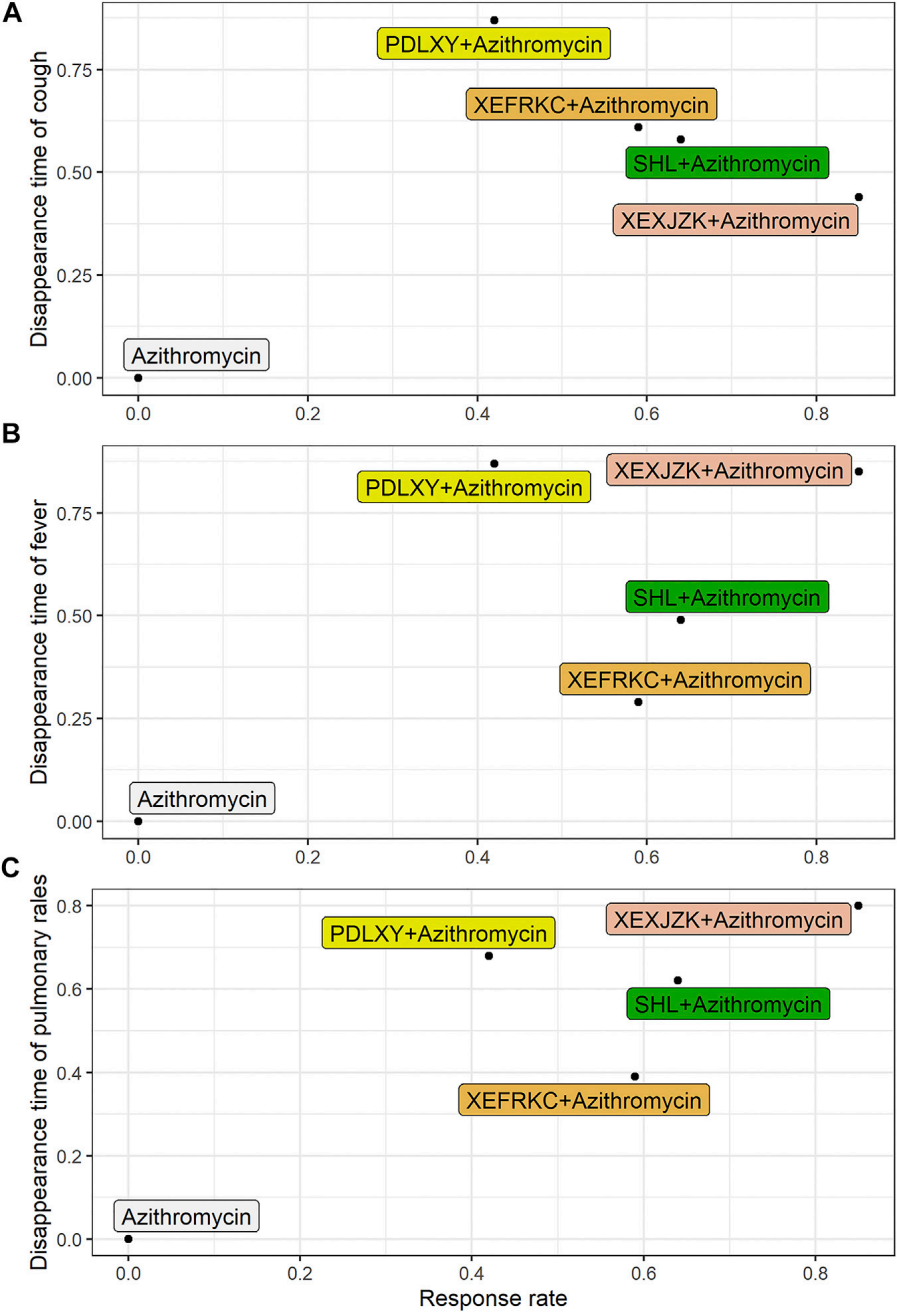
Biplot of Primary Outcomes. PDLXY: Pudilan Xiaoyan oral liquid; SHL: Shuanghuanlian oral liquid; XEFRKC: Xiaoer Feire Kechuan oral liquid; XRXJZK: Xiaoer Xiaoji Zhike oral liquid. **(A)** cough disappearance time + response rate; **(B)** fever disappearance time + response rate; **(C)** pulmonary rales disappearance time + response rate.

The results of three biplots further confirmed that XEXJZK + Azithromycin had the highest SUCRA value at 37.4, 72.25, and 68% among the eligible interventions (Figure 3 and Supplementary Table S4).

## Secondary Outcomes

### Related Clinical Indicators

#### Hospitalization Length

Twenty-nine RCTs reported hospitalization length after MPP treatment with three TCMOLs combined with azithromycin in 2,696 children. The network plot is illustrated in Supplementary Figure S5A.

The three interventions demonstrated significant differences compared with azithromycin (Supplementary Figure S6A). Moreover, the network analysis showed significant differences between SHL + Azithromycin and XEFRKC + Azithromycin (Supplementary Table S5).

According to SUCRA, SHL + Azithromycin (SUCRA: 92%) was superior to others, followed by XEXJZK + Azithromycin (SUCRA: 71%; Supplementary Figure S7A).

#### Pulmonary Shadow Disappearance Time

Seven RCTs reported disappearance time of pulmonary shadows in x-ray after MPP treatment with three TCMOLs combined with azithromycin in 551 children. The network plot is presented in Supplementary Figure S5B.

The three interventions demonstrated a significant difference compared with azithromycin (Supplementary Figure S6B). The network analysis showed significant differences between XEFRKC + Azithromycin and XEXJZK + Azithromycin (Supplementary Table S5).

According to SUCRA, XEXJZK + Azithromycin (SUCRA: 94%) was superior to others, followed by SHL + Azithromycin (SUCRA: 69%; Supplementary Figure S7B).

#### Inflammatory Cytokines

##### IL-6

Twelve RCTs reported IL-6 changes after MPP treatment with three TCMOLs combined with azithromycin in 1,281 children. The network plot is given in Supplementary Figure S8A.

Only two interventions indicated significant differences compared with azithromycin (Supplementary Figure S9A). The network analysis showed no significant differences among the interventions (Supplementary Table S6).

According to SUCRA, SHL + Azithromycin (SUCRA: 85%) was superior to others, followed by XEFRKC + Azithromycin (SUCRA: 59%; Supplementary Figure S10A).

##### TNF-α

Ten RCTs reported TNF-α changes after MPP treatment with four TCMOLs combined with azithromycin in 1,145 children. The network plot is shown in Supplementary Figure SB.

Only one intervention demonstrated a significant difference compared with azithromycin alone (Supplementary Figure S9B). The network analysis showed no significant difference among all three interventions (Supplementary Table S6).

According to SUCRA, SHL + Azithromycin (SUCRA: 94%) was superior to others, followed by XEFRKC + Azithromycin (SUCRA: 59%; Supplementary Figure S10B).

##### CRP

Nine RCTs reported CRP changes after MPP treatment with three TCMOLs combined with azithromycin in 1,087 children. The network plot is shown in Supplementary Figure S8C.

Only one intervention indicated significant differences compared with azithromycin alone (Supplementary Figure S9C). The network analysis demonstrated no significant difference among the four interventions (Supplementary Table S7).

According to SUCRA, XEFRKC + Azithromycin (SUCRA: 70%) was superior to others, followed by XEZJZK + Azithromycin (SUCRA: 66%; Supplementary Figure S10C).

### Safety

Regarding safety, 46.03, 38.1, 26.98, 26.98, and 14.29% of RCTs reported the number of AEs, ARs, abdominal pain and diarrhea incidence, nausea and vomiting incidence, and skin rash incidence.

#### AEs

Thirty-nine RCTs reported 158 and 224 AEs after MPP treatment with TCMOLs + Azithromycin and azithromycin alone, respectively (PDLXY + Azithromycin [4 trials, 9 AEs], SHL + Azithromycin [8 trials, 31 AEs], XEFRKC + Azithromycin [10 trials, 54 AEs], and XEXJZK + Azithromycin [17 trials, 64 AEs]; Supplementary Table S8).

#### ARs

Twenty-four RCTs reported ARs after MPP treatment with four TCMOLs combined with azithromycin. The network plot is shown in Supplementary Figure S11.

In terms of ARs and abdominal pain and diarrhea incidence, the difference between four interventions and azithromycin alone was nonsignificant (Supplementary Figures S12A,B). In terms of nausea and vomiting incidence and skin rash incidence, only one intervention demonstrated a significant difference compared with azithromycin alone (Supplementary Figures S12C,D).

### Meta-Regression

Meta-regression was used to analyze the five covariates of primary outcomes and further explore potential confounding factors that may affect outcomes: gender proportion, age, treatment duration, disease course, and drug delivery mode. The meta-regression results showed no obvious modifiers in the primary outcomes, indicating that the results were relatively stable (Supplementary Table S9 and Supplementary Figure S13).

### Publication Bias

Begg’s test was used to identify the possible publication bias related to the different interventions and the impact of small sample studies. Different interventions were marked with different colored points. The results demonstrated asymmetry distribution in the funnel plot of the response rate (*p* = 0.0019), indicating potential publication bias.

**FIGURE 4:**
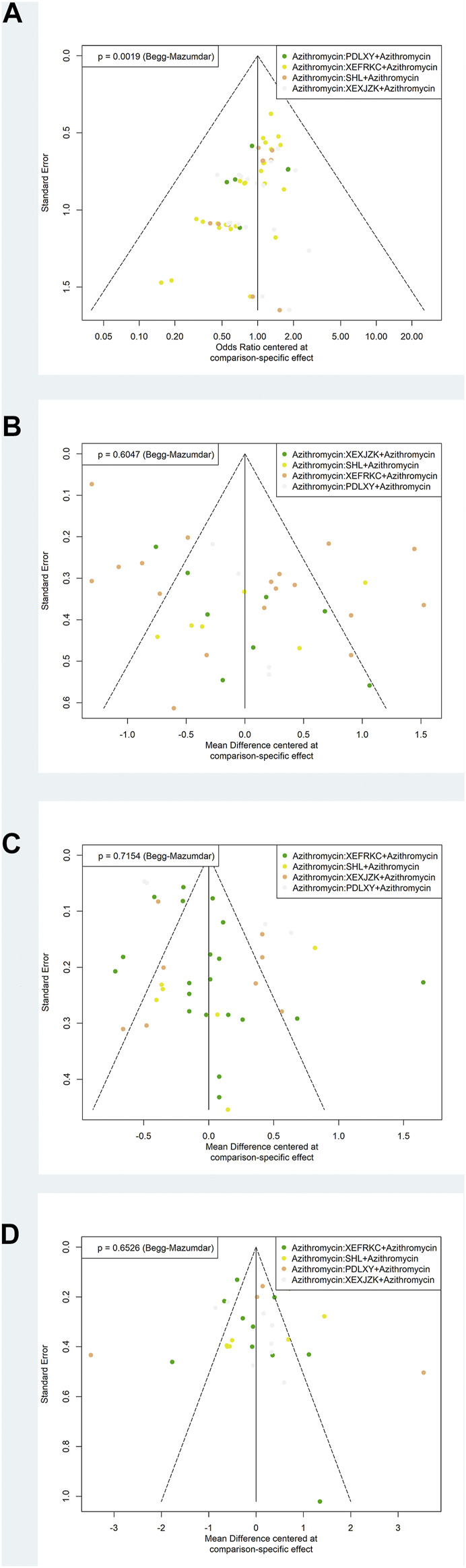
Funnel Plots of Primary Outcomes. PDLXY: Pudilan Xiaoyan oral liquid; SHL: Shuanghuanlian oral liquid; XEFRKC: Xiaoer Feire Kechuan oral liquid; XRXJZK: Xiaoer Xiaoji Zhike oral liquid. **(A)** response rate; **(B)** cough disappearance time; **(C)** fever disappearance time; **(D)** pulmonary rales disappearance time.

Finally, distributions in the funnel plot of the disappearance times of cough (*p* = 0.6047), fever (*p* = 0.7154), and pulmonary rales (*p* = 0.6526) were all symmetric (Figure 4).

## Discussion

### General Interpretations

Based on the 63 included RCTs on 6,410 children with MPP, we found that all TCMOLs combined with azithromycin demonstrated significantly better outcomes than azithromycin alone did—with respect to cough, fever, and pulmonary rales. Of the different TCMOLs, XEXJZK + Azithromycin showed the highest odds of response rate and shortened pulmonary rales disappearance time. Moreover, PDLXY + Azithromycin demonstrated the largest reductions in the cough and fever disappearance times. Furthermore, XEXJZK + Azithromycin shortened hospitalization length and pulmonary shadow disappearance time. No significant differences were found in terms of AEs; however, the related evidence is insufficient and requires further investigation.

In general, TCMOLs might be effective in relieving cough, fever and pulmonary rales in children with MPP. Specifically, XEXJZK and PDLXY might have the most significant effects. However, we noted that the related certainty of evidence was generally low to very low, suggesting that the research evaluating TCMOLs further is needed.

### Comparison With Other Studies

A previous meta-analysis on TCMOLs combined with azithromycin for treating MPP in children focused only on a single TCMOL’s efficacy and safety (Zhu et al., 2019; Li et al., 2020; Zhang et al., 2020). Although its results could aid clinicians in making clinical decisions, it could not indicate optimal effects of various TCMOLs in clinical practice. Meta-analysis and network meta-analysis of TCM injections (TCMIs) combined with azithromycin in the treatment of MPP in children have also been published (Duan et al., 2019). Their results have some instructive significance for the clinical applications of TCMIs. However, the national policies and the patients’ willingness in China might somewhat limit the TCMIs’ utility in the real-life clinical settings. Therefore, the current study focused on comparing the clinical efficacy and safety of different TCMOLs to select the best interventions, which is helpful for further clinical practice.

### Implications for Practice

The included TCMOLs (PDLXY, XEFRKC, XEXJZK, and SHL) are registered and approved for clinical use by State Food and Drug Administration. Because TCMOLs contains many TCM ingredients, the potential risk of impact in clinical applications may occur.

The safety and potential effects of high-frequency use of TCM ingredients (*Scutellaria baicalensis* and *Isatis tinctoria*) and possibly high-risk TCM ingredients (ephedra and areca nut) should be evaluated: *I. tinctoria* can cause allergic reactions and anaphylactoid reactions, even leading to urticaria, multiple granulomas, and hemolysis (Wang et al., 2012; Zhang, 2016). *S. baicalensis* can cause skin allergy (Lin et al., 2016); when used in large doses, it can cause maternal toxicity (Tian et al., 2009). Ephedrine alkaloids in ephedra can stimulate the central nervous system and sympathetic nervous system to produce adverse reactions, all of which can affect tissues including the myocardium, bladder smooth muscles, and external urethral sphincter (Odaguchi et al., 2019). In general, ephedra has many adverse events, including cardiovascular and cerebrovascular diseases (stroke, hypertension, palpitation, and heart attack), gastrointestinal diseases (nausea and vomiting), and mental disorders (anxiety) (Schulman, 2003). Areca nut not only has a carcinogenic, mutagenic effect but also has an effect on the reproductive system (Liu et al., 2013). In case of an overdose, the areca nut’s chemical components (alkaloids and tannins) can cause cardiotoxicity and neurotoxicity, even leading to death (Lin et al., 2018; Jia et al., 2020).

Due to a lack of professional knowledge, some patients think that TCM is safe, and thus, they use it uninterrupted over a long term and eventually overdose (Ernst, 2007). Excessive intake of TCM can not only cause an increase in adverse reactions but also affect the effectiveness of clinical treatment (Schulman, 2003). The incidence of adverse events and diseases related to TCM herbs has newly become a serious public problem; thus, cautious clinical use of TCMOLs is advised by experts and by various guidelines.

### Strengths and Limitations of This Study

#### Strengths

We used the Bayesian approach to compare various TCMOLs in combination with azithromycin for treating MPP in children. To select the best TCMOL-based interventions and generate a better guide for clinical decision-making based on TCM, we conducted a detailed literature review, which made the included literature more comprehensive and made our results more plausible. The inclusion and exclusion criteria were highly stringent. The interventions in all included studies involved azithromycin alone and in combination with TCMOLs—all reducing the effect of the potential heterogeneity and making the results more precise.

Here, we considered not only response rate but also cough, fever, and pulmonary rales disappearance times as primary outcomes. Because the response rate and other primary outcomes are strongly correlated, we drew a biplot to further improve our results’ accuracy. To eliminate the potential confounding factors and ensure the stability of our results, we performed meta-regression of five covariates that potentially affected the primary outcomes.

#### Limitations

Although we applied no limitations on the study population and location, all the included studies were found to be conducted in China; this may have caused risk of bias.

There were also some deficiencies in the methodological quality of these studies. The random sequence generation of some studies was high-risk, and methods of the random sequence generation were not fully mentioned in most studies. Only two trials reported blinding, and none mentioned allocation concealment.

The small sample also made it difficult to detect significant differences between the treatment and control groups.

Regarding the secondary outcomes, few studies reported medical imaging (pulmonary shadows in x-ray) and laboratory testing (inflammatory cytokine and CRP levels). Thus, it was impossible to compare the superiority or inferiority of all the interventions.

There was no direct comparative study on various TCMOLs combined with azithromycin found. Therefore, we used Bayesian network meta-analysis to make the indirect comparison, which may have caused inconsistency between direct and indirect comparisons.

## Conclusion

In conclusion, XEXJZK + Azithromycin, with the highest SUCRA value, may be the best treatment option for MPP in children. However, because of the low certainty of evidence, caution should be exercised when interpreting this result. Although the evidence of safety did not show the significant difference between TCMOLs + Azithromycin and azithromycin alone, the adverse effects remained unclear, mainly due to the lack of high-quality evidence. The dissemination of the evidence should also be cautioned due to the limitations. Finally, high-quality RCTs with focus on patient-important outcomes are thus required.

## Data Availability

The raw data supporting the conclusion of this article will be made available by the authors, without undue reservation.
