# Supplementary material for: Traditional Chinese Medicine Oral Liquids Combined With Azithromycin for Mycoplasma pneumoniae Pneumonia in Children: A Bayesian Network Meta-Analysis
Source: Front Pharmacol. 2021 May 28;12:652412. doi: 10.3389/fphar.2021.652412 (PMC8194818; doi:10.3389/fphar.2021.652412)
Supplement: Supplementary file 1 [file DataSheet1.DOCX]

**Supplementary Material**

**Contents of Supplementary Figure, Table, File, and PRISMA checklist**

[**Figure 1 Risk of Bias of Included Studies 3**](#_Toc69202209)

[**Figure 2 Risk of Bias Summary 4**](#_Toc69202210)

[**Figure 3 Forest Plots of Primary Outcomes 5**](#_Toc69202211)

[**Figure 4 Rank Plots and SUCRA of Primary Outcomes 6**](#_Toc69202212)

[**Figure 5 Network Plots of Length of Hospitalization Time and Disappearance Time of Pulmonary Shadows in X-ray 7**](#_Toc69202213)

[**Figure 6 Forest Plots of Length of Hospitalization Time and Disappearance Time of Pulmonary Shadows in X-ray 8**](#_Toc69202214)

[**Figure 7 Rank Plots and SUCRA of Length of Hospitalization Time and Disappearance Time of Pulmonary Shadows in X-ray 9**](#_Toc69202215)

[**Figure 8 Network Plots of Inflammatory Cytokine 10**](#_Toc69202216)

[**Figure 9 Forest Plots of Inflammatory Cytokine 11**](#_Toc69202217)

[**Figure 10 Rank Plots and SUCRA of Inflammatory Cytokine 12**](#_Toc69202218)

[**Figure 11 Network Plots of Safety 13**](#_Toc69202219)

[**Figure 12 Forest Plots of Safety 14**](#_Toc69202220)

[**Figure 13 The Meta-regression of Primary Outcomes 15**](#_Toc69202221)

[**Table 1 Summary Table of All Included Studies 16**](#_Toc69202222)

[**Table 2 Detailed Chemical Characterizations of Traditional Chinese Medicine Oral Liquids 32**](#_Toc69202223)

[**Table 3 Characteristics of Included Studies 34**](#_Toc69202224)

[**Table 4 The SUCRA of Primary Outcomes in Biplot 43**](#_Toc69202225)

[**Table 5 League table of Length of Hospitalization Time and Disappearance Time of Pulmonary Shadows in X-ray 44**](#_Toc69202226)

[**Table 6 League Table of Interleukin-6 and Tumor Necrosis Factor-α 44**](#_Toc69202227)

[**Table 7 League Table of C-reactive Protein 46**](#_Toc69202228)

[**Table 8 Adverse Events 47**](#_Toc69202229)

[**Table 9 The Meta-regression for Primary Outcomes 50**](#_Toc69202230)

[**File 1 The Detailed Search Strategy 55**](#_Toc69202231)

[**File 2 Reference of Included Studies 56**](#_Toc69202232)

[**PRISMA Checklist 61**](#_Toc69202233)

# Figure 1 Risk of Bias of Included Studies


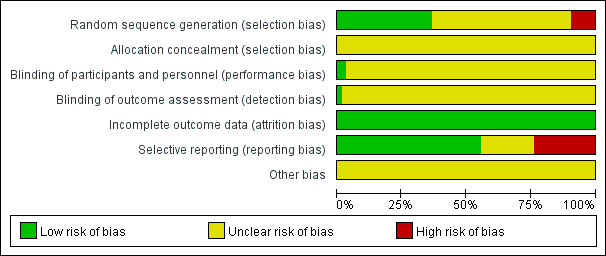


# Figure 2 Risk of Bias Summary


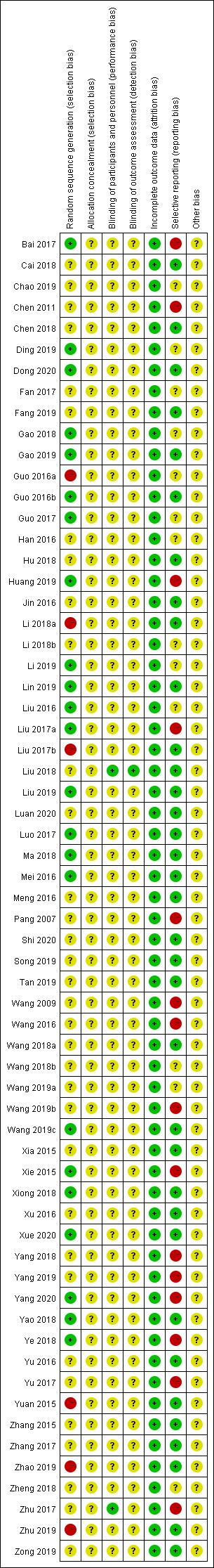


#

# Figure 3 Forest Plots of Primary Outcomes


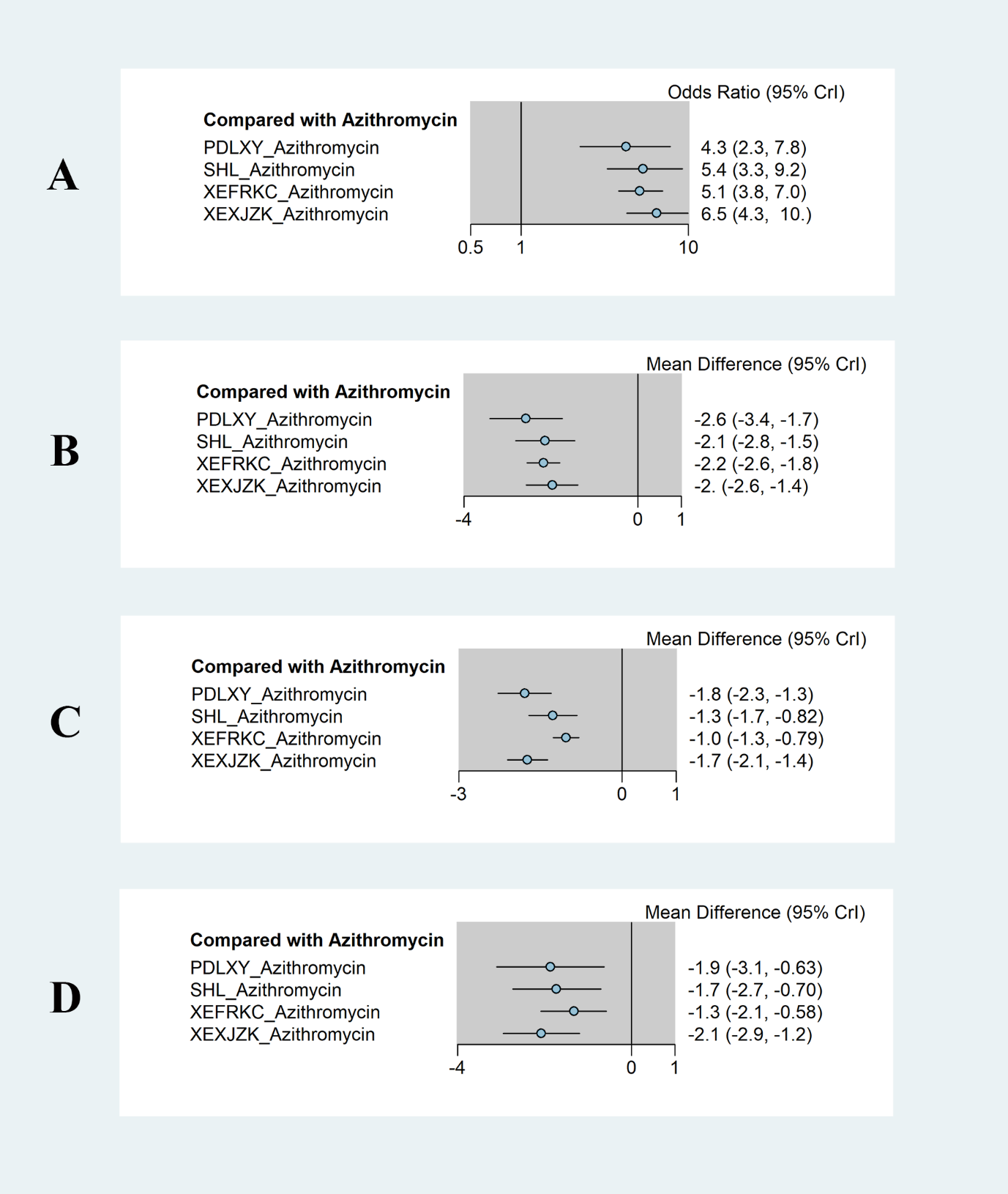


(PDLXY: Pudilan Xiaoyan oral liquid; SHL: Shuanghuanlian oral liquid; XEFRKC: Xiaoer Feire Kechuan oral liquid; XRXJZK: Xiaoer Xiaoji Zhike oral liquid. **(A)** response rate; **(B)** disappearance time of cough; **(C)** disappearance time of fever; **(D)** disappearance time of pulmonary rales)

# Figure 4 Rank Plots and SUCRA of Primary Outcomes


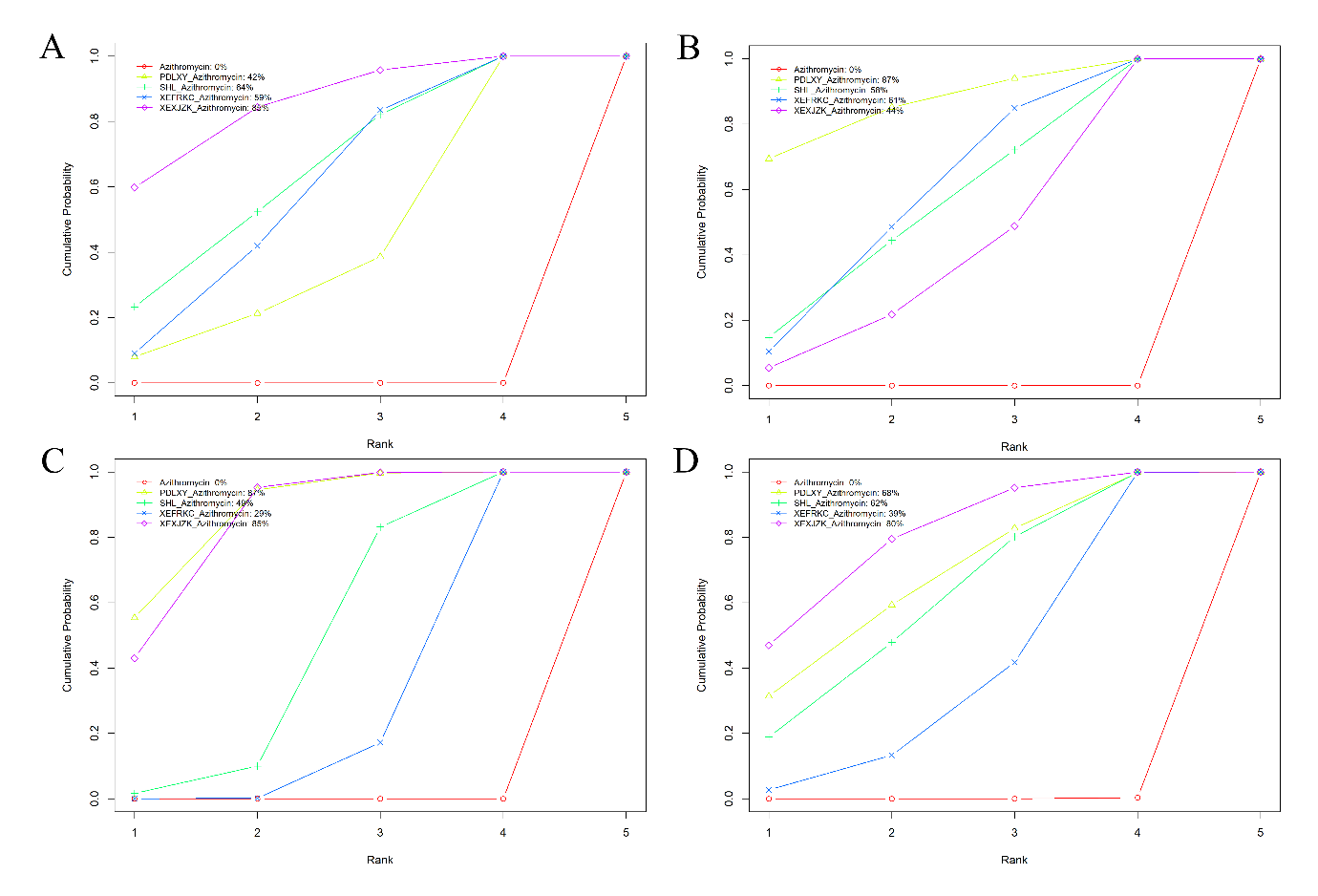


(PDLXY: Pudilan Xiaoyan oral liquid; SHL: Shuanghuanlian oral liquid; XEFRKC: Xiaoer Feire Kechuan oral liquid; XRXJZK: Xiaoer Xiaoji Zhike oral liquid. **(A)** response rate; **(B)** disappearance time of cough; **(C)** disappearance time of fever; **(D)** disappearance time of pulmonary rales)

# Figure 5 Network Plots of Length of Hospitalization Time and Disappearance Time of Pulmonary Shadows in X-ray


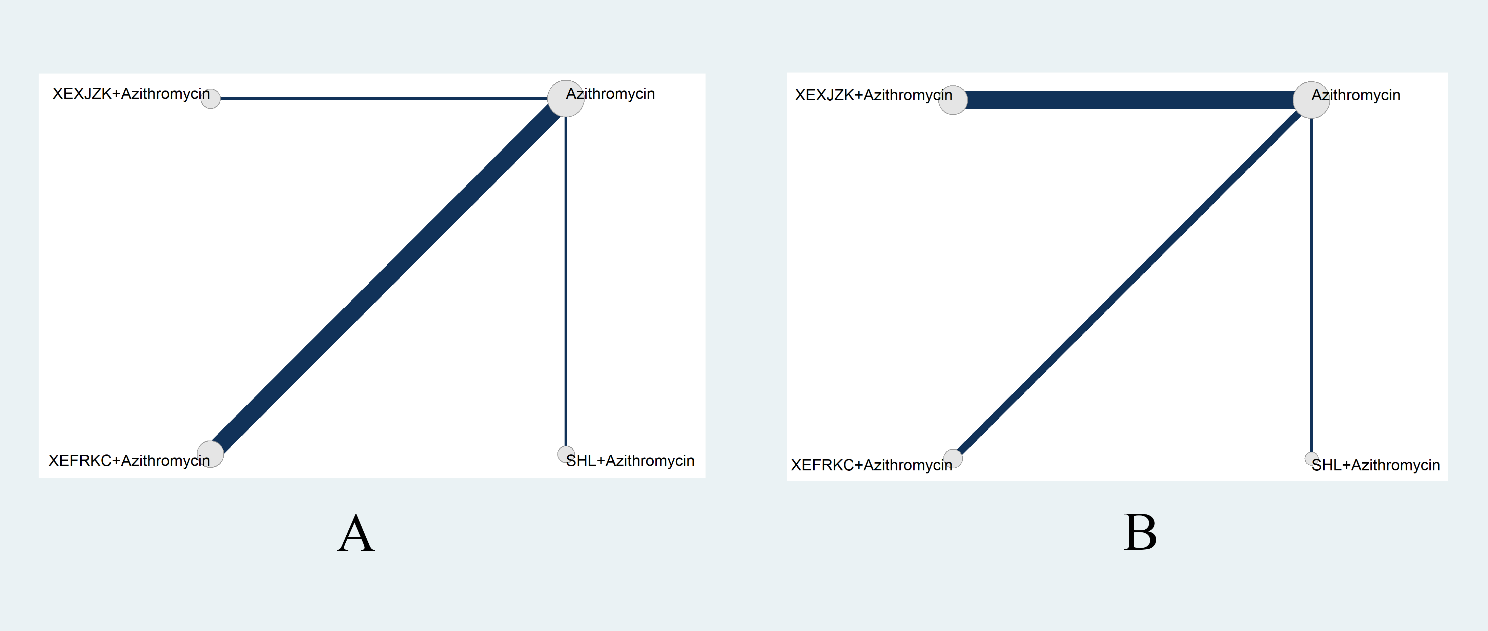


(PDLXY: Pudilan Xiaoyan oral liquid; SHL: Shuanghuanlian oral liquid; XEFRKC: Xiaoer Feire Kechuan oral liquid; XRXJZK: Xiaoer Xiaoji Zhike oral liquid. **(A)** average hospitalization time; **(B)** disappearance time of pulmonary shadows in X-ray)

# Figure 6 Forest Plots of Length of Hospitalization Time and Disappearance Time of Pulmonary Shadows in X-ray
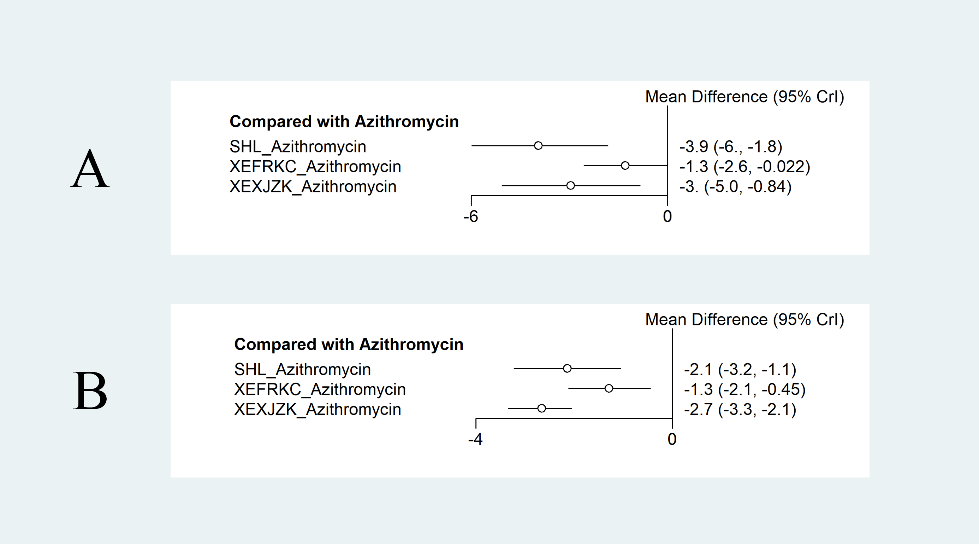


(PDLXY: Pudilan Xiaoyan oral liquid; SHL: Shuanghuanlian oral liquid; XEFRKC: Xiaoer Feire Kechuan oral liquid; XRXJZK: Xiaoer Xiaoji Zhike oral liquid. **(A)** average hospitalization time; **(B)** disappearance time of pulmonary shadows in X-ray)

# Figure 7 Rank Plots and SUCRA of Length of Hospitalization Time and Disappearance Time of Pulmonary Shadows in X-ray


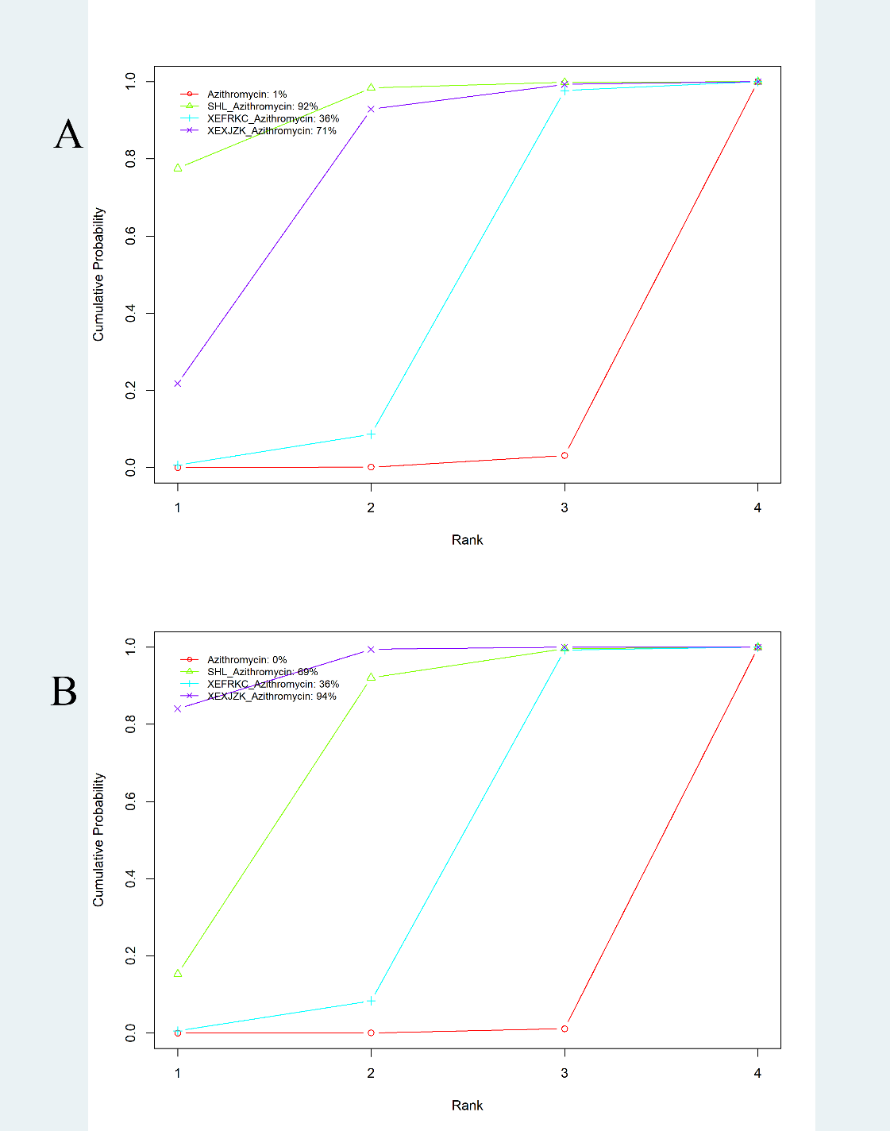


(PDLXY: Pudilan Xiaoyan oral liquid; SHL: Shuanghuanlian oral liquid; XEFRKC: Xiaoer Feire Kechuan oral liquid; XRXJZK: Xiaoer Xiaoji Zhike oral liquid. **(A)** average hospitalization time; **(B)** disappearance time of pulmonary shadows in X-ray)

# Figure 8 Network Plots of Inflammatory Cytokine


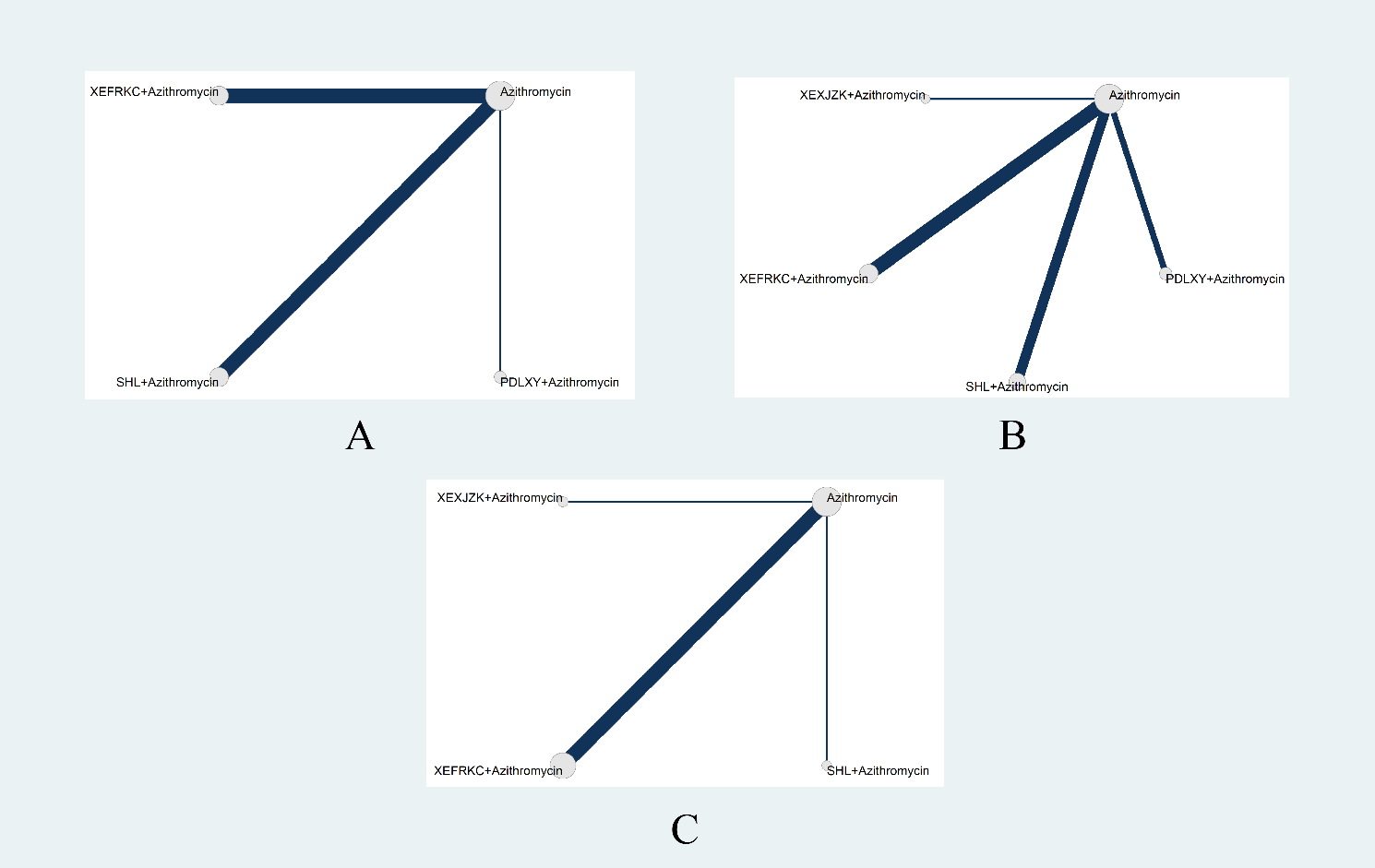


(PDLXY: Pudilan Xiaoyan oral liquid; SHL: Shuanghuanlian oral liquid; XEFRKC: Xiaoer Feire Kechuan oral liquid; XRXJZK: Xiaoer Xiaoji Zhike oral liquid. (A) Interleukin-6; (B) Tumor necrosis factor-α; (C) C-reactive protein)

# Figure 9 Forest Plots of Inflammatory Cytokine


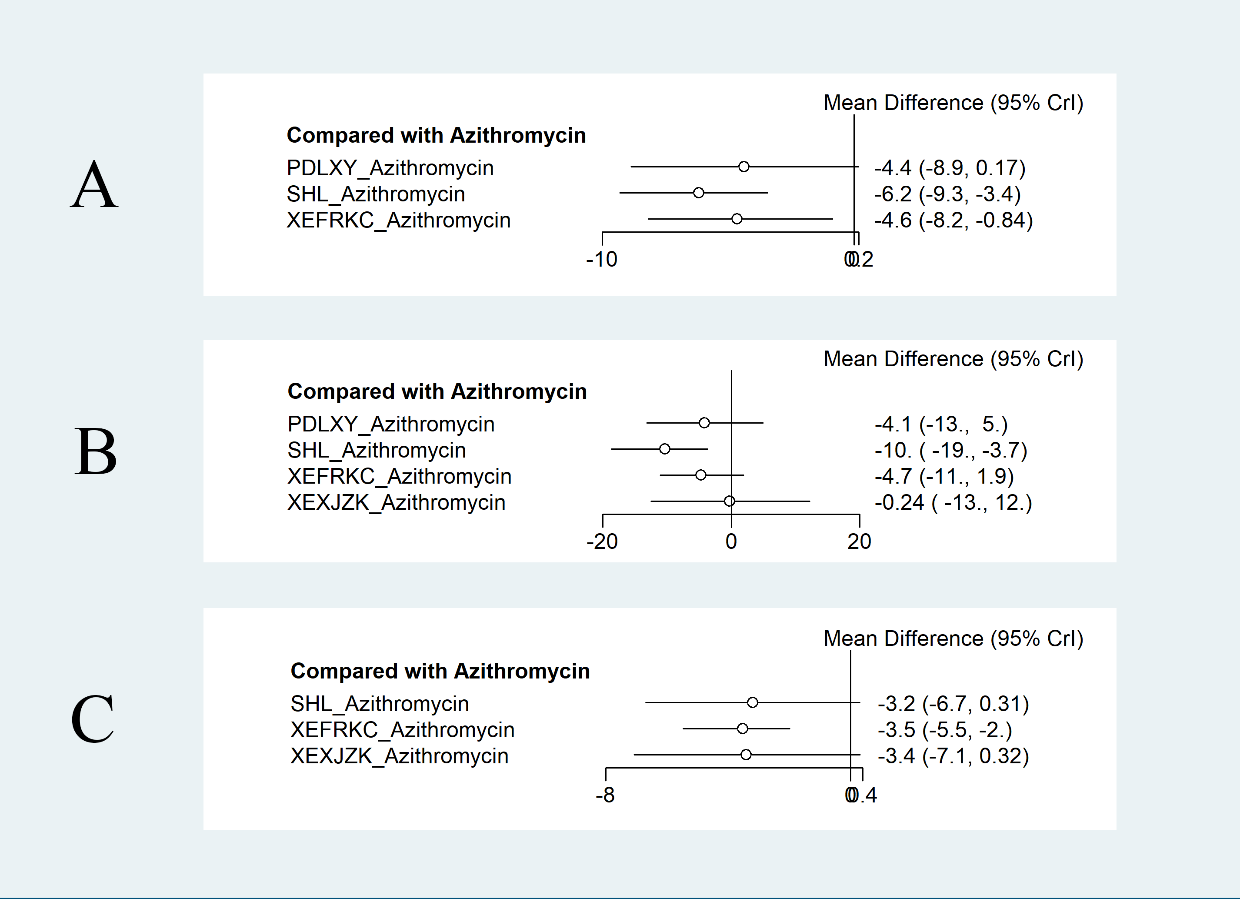


(PDLXY: Pudilan Xiaoyan oral liquid; SHL: Shuanghuanlian oral liquid; XEFRKC: Xiaoer Feire Kechuan oral liquid; XRXJZK: Xiaoer Xiaoji Zhike oral liquid. (A) Interleukin-6; (B) Tumor necrosis factor-α; (C) C-reactive protein)

# Figure 10 Rank Plots and SUCRA of Inflammatory Cytokine


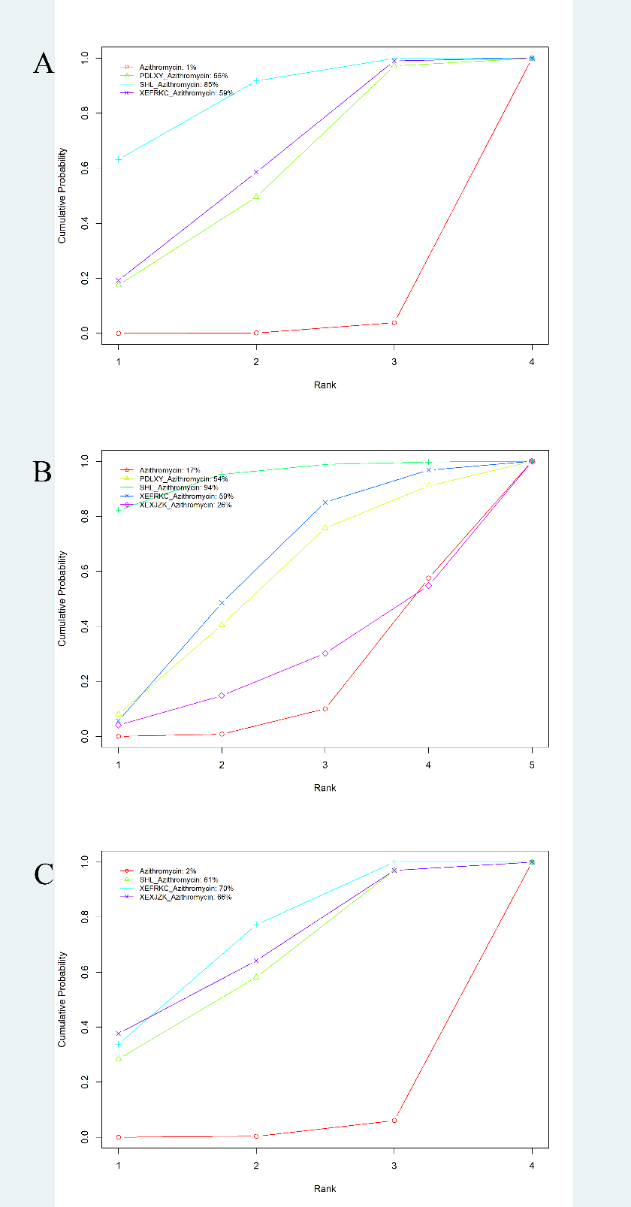


(PDLXY: Pudilan Xiaoyan oral liquid; SHL: Shuanghuanlian oral liquid; XEFRKC: Xiaoer Feire Kechuan oral liquid; XRXJZK: Xiaoer Xiaoji Zhike oral liquid. (A) Interleukin-6; (B) Tumor necrosis factor-α; (C) C-reactive protein)

# Figure 11 Network Plots of Safety


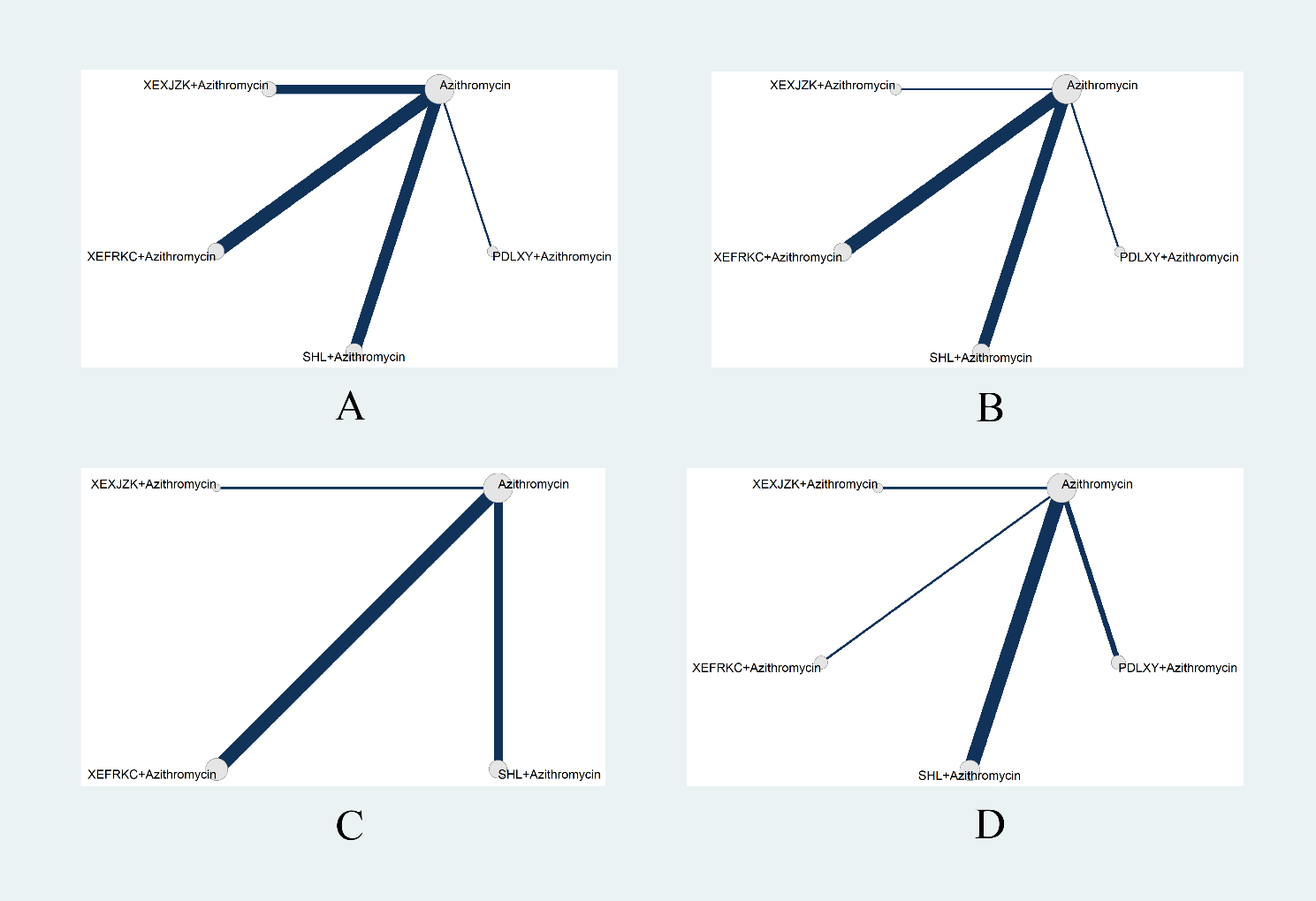


(PDLXY: Pudilan Xiaoyan oral liquid; SHL: Shuanghuanlian oral liquid; XEFRKC: Xiaoer Feire Kechuan oral liquid; XRXJZK: Xiaoer Xiaoji Zhike oral liquid. (A) adverse rate; (B) abdominal pain and diarrhea; (C) nausea and vomiting; (D) skin rash)

## Figure 12 Forest Plots of Safety


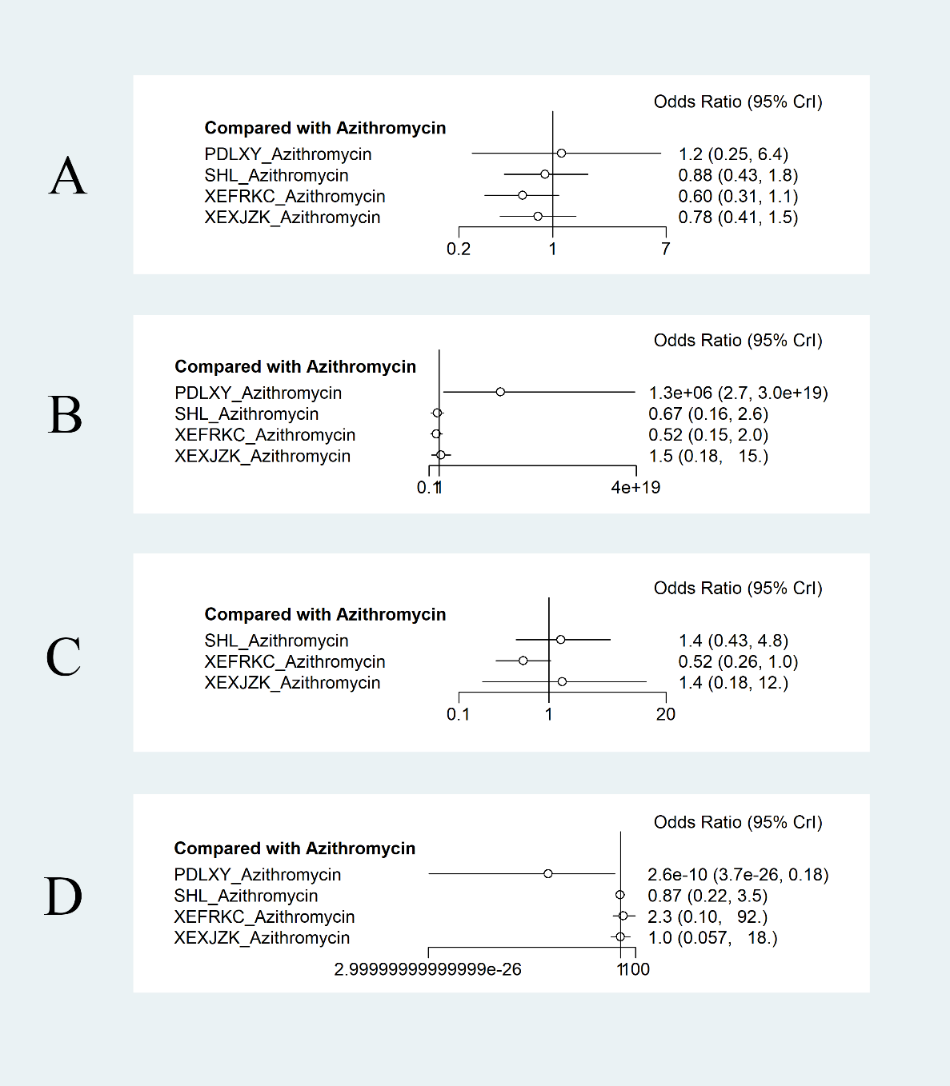


(PDLXY: Pudilan Xiaoyan oral liquid; SHL: Shuanghuanlian oral liquid; XEFRKC: Xiaoer Feire Kechuan oral liquid; XRXJZK: Xiaoer Xiaoji Zhike oral liquid. (A) adverse rate; (B) abdominal pain and diarrhea; (C) nausea and vomiting; (D) skin rash)

## Figure 13 The Meta-regression of Primary Outcomes


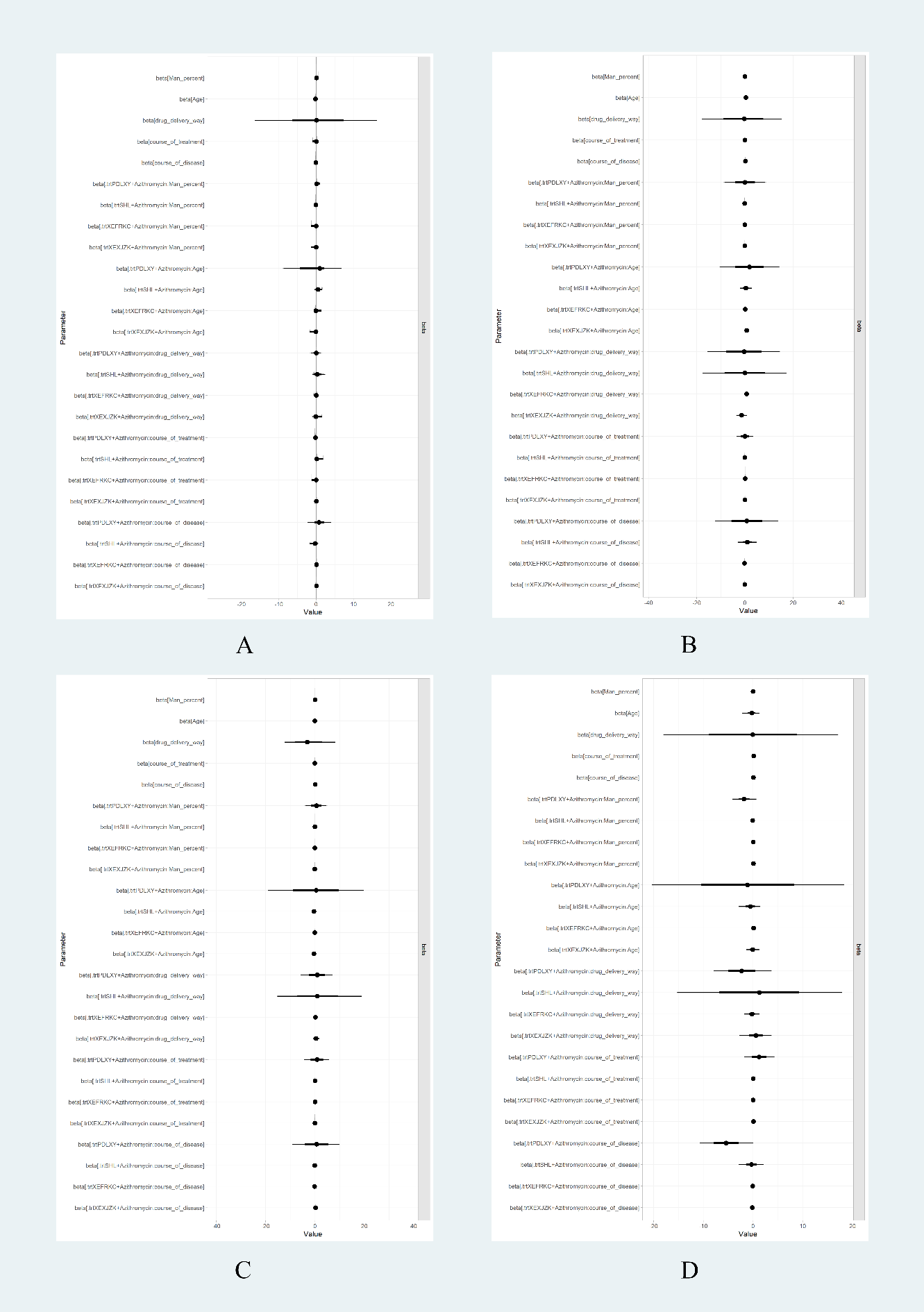


(PDLXY: Pudilan Xiaoyan oral liquid; SHL: Shuanghuanlian oral liquid; XEFRKC: Xiaoer Feire Kechuan oral liquid; XRXJZK: Xiaoer Xiaoji Zhike oral liquid. **(A)** response rate; **(B)** disappearance time of cough; **(C)** disappearance time of pulmonary rales; **(D)** disappearance time of pulmonary rales)

# Table 1 Summary Table of All Included Studies

| **Study** | **Formulation** | **Source** | **Raw material** | **Quality control reported? (Y/N)** | **Chemical analysis reported? (Y/N)** |
| --- | --- | --- | --- | --- | --- |
| Fan and He, 2017 | Shuanghuanglian oral liquid | Henan Tailong Pharmaceutical Co., Ltd. (SFDA approval number: Z41020565) | Lonicera japonica Thunb. [Caprifoliaceae](Lonicera japonica); Scutellaria baicalensis Georgi [Lamiaceae](baical skullcap root); Forsythia suspensa (Thunb.) Vahl [Oleaceae](fructus forsythiae suspensae) | Y - Prepared according to People's Republic of China Pharmacopoeia | N |
| Guo and He, 2017 | Shuanghuanglian oral liquid | Henan Tailong Pharmaceutical Co., Ltd. (SFDA approval number: Z41020565) | Lonicera japonica Thunb. [Caprifoliaceae](Lonicera japonica); Scutellaria baicalensis Georgi [Lamiaceae](baical skullcap root); Forsythia suspensa (Thunb.) Vahl [Oleaceae](fructus forsythiae suspensae) | Y - Prepared according to People's Republic of China Pharmacopoeia | N |
| Liu, 2019 | Shuanghuanglian oral liquid | Harbin Pharmaceutical Group Sanjing Pharmaceutical Co., Ltd. (SFDA approval number: Z10920053) | Lonicera japonica Thunb. [Caprifoliaceae](Lonicera japonica); Scutellaria baicalensis Georgi [Lamiaceae](baical skullcap root); Forsythia suspensa (Thunb.) Vahl [Oleaceae](fructus forsythiae suspensae) | Y - Prepared according to People's Republic of China Pharmacopoeia | N |
| Liu et al., 2016 | Shuanghuanglian oral liquid | Harbin huili Pharmaceutical Co., Ltd. (SFDA approval number: Z2302092) | Lonicera japonica Thunb. [Caprifoliaceae](Lonicera japonica); Scutellaria baicalensis Georgi [Lamiaceae](baical skullcap root); Forsythia suspensa (Thunb.) Vahl [Oleaceae](fructus forsythiae suspensae) | Y - Prepared according to People's Republic of China Pharmacopoeia | N |
| Liu, 2018 | Shuanghuanglian oral liquid | Henan Tailong Pharmaceutical Co., Ltd. (SFDA approval number: Z41020565) | Lonicera japonica Thunb. [Caprifoliaceae](Lonicera japonica); Scutellaria baicalensis Georgi [Lamiaceae](baical skullcap root); Forsythia suspensa (Thunb.) Vahl [Oleaceae](fructus forsythiae suspensae) | Y - Prepared according to People's Republic of China Pharmacopoeia | N |
| Shi, 2020 | Shuanghuanglian oral liquid | Henan Tailong Pharmaceutical Co., Ltd. (SFDA approval number: Z41020565) | Lonicera japonica Thunb. [Caprifoliaceae](Lonicera japonica); Scutellaria baicalensis Georgi [Lamiaceae](baical skullcap root); Forsythia suspensa (Thunb.) Vahl [Oleaceae](fructus forsythiae suspensae) | Y - Prepared according to People's Republic of China Pharmacopoeia | N |
| Wang, 2018 | Shuanghuanglian oral liquid | Henan Tailong Pharmaceutical Co., Ltd. (SFDA approval number: Z41020565) | Lonicera japonica Thunb. [Caprifoliaceae](Lonicera japonica); Scutellaria baicalensis Georgi [Lamiaceae](baical skullcap root); Forsythia suspensa (Thunb.) Vahl [Oleaceae](fructus forsythiae suspensae) | Y - Prepared according to People's Republic of China Pharmacopoeia | N |
| Wang et al., 2018 | Shuanghuanglian oral liquid | Harbin Pharmaceutical Group Sanjing Pharmaceutical Co., Ltd. (SFDA approval number: Z10920053) | Lonicera japonica Thunb. [Caprifoliaceae](Lonicera japonica); Scutellaria baicalensis Georgi [Lamiaceae](baical skullcap root); Forsythia suspensa (Thunb.) Vahl [Oleaceae](fructus forsythiae suspensae) | Y - Prepared according to People's Republic of China Pharmacopoeia | N |
| Yao, 2018 | Shuanghuanglian oral liquid | Henan Tailong Pharmaceutical Co., Ltd. (SFDA approval number: Z41020565) | Lonicera japonica Thunb. [Caprifoliaceae](Lonicera japonica); Scutellaria baicalensis Georgi [Lamiaceae](baical skullcap root); Forsythia suspensa (Thunb.) Vahl [Oleaceae](fructus forsythiae suspensae) | Y - Prepared according to People's Republic of China Pharmacopoeia | N |
| Zhang, 2017 | Shuanghuanglian oral liquid | Henan Tailong Pharmaceutical Co., Ltd. (SFDA approval number: Z41020565) | Lonicera japonica Thunb. [Caprifoliaceae](Lonicera japonica); Scutellaria baicalensis Georgi [Lamiaceae](baical skullcap root); Forsythia suspensa (Thunb.) Vahl [Oleaceae](fructus forsythiae suspensae) | Y - Prepared according to People's Republic of China Pharmacopoeia | N |
| Gao and Wu, 2019 | Pudilan Xiaoyan oral liquid | Jumpcan Pharmaceutical Co., Ltd. (SFDA approval number: Z20030095) | Taraxacum officinale F.H.Wigg. [Asteraceae]; Isatis tinctoria L. [Brassicaceae](Isatidis Radix);  Corydalis bungeana Turcz. [Papaveraceae] (bunge corydalis herb); Scutellaria baicalensis Georgi [Lamiaceae](baical skullcap root) | Y - Prepared according to People's Republic of China Pharmacopoeia | N |
| Guo, 2016a | Pudilan Xiaoyan oral liquid | Jumpcan Pharmaceutical Co., Ltd. (SFDA approval number: Z20030095) | Taraxacum officinale F.H.Wigg. [Asteraceae]; Isatis tinctoria L. [Brassicaceae](Isatidis Radix);  Corydalis bungeana Turcz. [Papaveraceae] (bunge corydalis herb); Scutellaria baicalensis Georgi [Lamiaceae](baical skullcap root) | Y - Prepared according to People's Republic of China Pharmacopoeia | N |
| Guo, 2016b | Pudilan Xiaoyan oral liquid | Prepared by Guo, 2016b | Taraxacum officinale F.H.Wigg. [Asteraceae]; Isatis tinctoria L. [Brassicaceae](Isatidis Radix);  Corydalis bungeana Turcz. [Papaveraceae] (bunge corydalis herb); Scutellaria baicalensis Georgi [Lamiaceae](baical skullcap root) | Y - Prepared according to People's Republic of China Pharmacopoeia | N |
| Wang 2009 | Pudilan Xiaoyan oral liquid | Jumpcan Pharmaceutical Co., Ltd. (SFDA approval number: Z20030095) | Taraxacum officinale F.H.Wigg. [Asteraceae]; Isatis tinctoria L. [Brassicaceae](Isatidis Radix);  Corydalis bungeana Turcz. [Papaveraceae] (bunge corydalis herb); Scutellaria baicalensis Georgi [Lamiaceae](baical skullcap root) | Y - Prepared according to People's Republic of China Pharmacopoeia | N |
| Xia et al., 2015 | Pudilan Xiaoyan oral liquid | Prepared by Xia et al., 2015 | Taraxacum officinale F.H.Wigg. [Asteraceae]; Isatis tinctoria L. [Brassicaceae](Isatidis Radix);  Corydalis bungeana Turcz. [Papaveraceae] (bunge corydalis herb); Scutellaria baicalensis Georgi [Lamiaceae](baical skullcap root) | Y - Prepared according to People's Republic of China Pharmacopoeia | N |
| Zhang et al., 2015 | Pudilan Xiaoyan oral liquid | Jumpcan Pharmaceutical Co., Ltd. (SFDA approval number: Z20030095) | Taraxacum officinale F.H.Wigg. [Asteraceae]; Isatis tinctoria L. [Brassicaceae](Isatidis Radix);  Corydalis bungeana Turcz. [Papaveraceae] (bunge corydalis herb); Scutellaria baicalensis Georgi [Lamiaceae](baical skullcap root) | Y - Prepared according to People's Republic of China Pharmacopoeia | N |
| Bai, 2017 | Xiaoer Feire Kechuan oral liquid | Heilongjiang sunflower Pharmaceutical Co., Ltd. (SFDA approval number: Z10950080) | Ephedra sinica Stapf [Ephedraceae](chinese ephedra); Corydalis bungeana Turcz. [Papaveraceae] (bunge corydalis herb); Gypsum (CaSO4·2H2O); Lonicera japonica Thunb. [Caprifoliaceae](Lonicera japonica); Forsythia suspensa (Thunb.) Vahl [Oleaceae](fructus forsythiae suspensae); Anemarrhena asphodeloides Bunge (Common Anemarrhena Rhizome); Scutellaria baicalensis Georgi [Lamiaceae](baical skullcap root); Isatis tinctoria L. [Brassicaceae](Isatidis Radix); Ophiopogon japonicus (Thunb.) Ker Gawl.(Radix Ophiopogonis); Houttuynia cordata Thunb. [Saururaceae](heartleaf houttuynia herb) | Y - Prepared according to People's Republic of China Pharmacopoeia | N |
| Cai, 2018 | Xiaoer Feire Kechuan oral liquid | Heilongjiang sunflower Pharmaceutical Co., Ltd. (SFDA approval number: Z10950080) | Ephedra sinica Stapf [Ephedraceae](chinese ephedra); Corydalis bungeana Turcz. [Papaveraceae] (bunge corydalis herb); Gypsum (CaSO4·2H2O); Lonicera japonica Thunb. [Caprifoliaceae](Lonicera japonica); Forsythia suspensa (Thunb.) Vahl [Oleaceae](fructus forsythiae suspensae); Anemarrhena asphodeloides Bunge (Common Anemarrhena Rhizome); Scutellaria baicalensis Georgi [Lamiaceae](baical skullcap root); Isatis tinctoria L. [Brassicaceae](Isatidis Radix); Ophiopogon japonicus (Thunb.) Ker Gawl.(Radix Ophiopogonis); Houttuynia cordata Thunb. [Saururaceae](heartleaf houttuynia herb) | Y - Prepared according to People's Republic of China Pharmacopoeia | N |
| Chen, 2011 | Xiaoer Feire Kechuan oral liquid | Heilongjiang sunflower Pharmaceutical Co., Ltd. (SFDA approval number: Z10950080) | Ephedra sinica Stapf [Ephedraceae](chinese ephedra); Corydalis bungeana Turcz. [Papaveraceae] (bunge corydalis herb); Gypsum (CaSO4·2H2O); Lonicera japonica Thunb. [Caprifoliaceae](Lonicera japonica); Forsythia suspensa (Thunb.) Vahl [Oleaceae](fructus forsythiae suspensae); Anemarrhena asphodeloides Bunge (Common Anemarrhena Rhizome); Scutellaria baicalensis Georgi [Lamiaceae](baical skullcap root); Isatis tinctoria L. [Brassicaceae](Isatidis Radix); Ophiopogon japonicus (Thunb.) Ker Gawl.(Radix Ophiopogonis); Houttuynia cordata Thunb. [Saururaceae](heartleaf houttuynia herb) | Y - Prepared according to People's Republic of China Pharmacopoeia | N |
| Ding and Hu, 2019 | Xiaoer Feire Kechuan oral liquid | Heilongjiang sunflower Pharmaceutical Co., Ltd. (SFDA approval number: Z10950080) | Ephedra sinica Stapf [Ephedraceae](chinese ephedra); Corydalis bungeana Turcz. [Papaveraceae] (bunge corydalis herb); Gypsum (CaSO4·2H2O); Lonicera japonica Thunb. [Caprifoliaceae](Lonicera japonica); Forsythia suspensa (Thunb.) Vahl [Oleaceae](fructus forsythiae suspensae); Anemarrhena asphodeloides Bunge (Common Anemarrhena Rhizome); Scutellaria baicalensis Georgi [Lamiaceae](baical skullcap root); Isatis tinctoria L. [Brassicaceae](Isatidis Radix); Ophiopogon japonicus (Thunb.) Ker Gawl.(Radix Ophiopogonis); Houttuynia cordata Thunb. [Saururaceae](heartleaf houttuynia herb) | Y - Prepared according to People's Republic of China Pharmacopoeia | N |
| Dong et al., 2020 | Xiaoer Feire Kechuan oral liquid | Heilongjiang sunflower Pharmaceutical Co., Ltd. (SFDA approval number: Z10950080) | Ephedra sinica Stapf [Ephedraceae](chinese ephedra); Corydalis bungeana Turcz. [Papaveraceae] (bunge corydalis herb); Gypsum (CaSO4·2H2O); Lonicera japonica Thunb. [Caprifoliaceae](Lonicera japonica); Forsythia suspensa (Thunb.) Vahl [Oleaceae](fructus forsythiae suspensae); Anemarrhena asphodeloides Bunge (Common Anemarrhena Rhizome); Scutellaria baicalensis Georgi [Lamiaceae](baical skullcap root); Isatis tinctoria L. [Brassicaceae](Isatidis Radix); Ophiopogon japonicus (Thunb.) Ker Gawl.(Radix Ophiopogonis); Houttuynia cordata Thunb. [Saururaceae](heartleaf houttuynia herb) | Y - Prepared according to People's Republic of China Pharmacopoeia | N |
| Fang, 2019 | Xiaoer Feire Kechuan oral liquid | Heilongjiang sunflower Pharmaceutical Co., Ltd. (SFDA approval number: Z10950080) | Ephedra sinica Stapf [Ephedraceae](chinese ephedra); Corydalis bungeana Turcz. [Papaveraceae] (bunge corydalis herb); Gypsum (CaSO4·2H2O); Lonicera japonica Thunb. [Caprifoliaceae](Lonicera japonica); Forsythia suspensa (Thunb.) Vahl [Oleaceae](fructus forsythiae suspensae); Anemarrhena asphodeloides Bunge (Common Anemarrhena Rhizome); Scutellaria baicalensis Georgi [Lamiaceae](baical skullcap root); Isatis tinctoria L. [Brassicaceae](Isatidis Radix); Ophiopogon japonicus (Thunb.) Ker Gawl.(Radix Ophiopogonis); Houttuynia cordata Thunb. [Saururaceae](heartleaf houttuynia herb) | Y - Prepared according to People's Republic of China Pharmacopoeia | N |
| Gao, 2018 | Xiaoer Feire Kechuan oral liquid | Heilongjiang sunflower Pharmaceutical Co., Ltd. (SFDA approval number: Z10950080) | Ephedra sinica Stapf [Ephedraceae](chinese ephedra); Corydalis bungeana Turcz. [Papaveraceae] (bunge corydalis herb); Gypsum (CaSO4·2H2O); Lonicera japonica Thunb. [Caprifoliaceae](Lonicera japonica); Forsythia suspensa (Thunb.) Vahl [Oleaceae](fructus forsythiae suspensae); Anemarrhena asphodeloides Bunge (Common Anemarrhena Rhizome); Scutellaria baicalensis Georgi [Lamiaceae](baical skullcap root); Isatis tinctoria L. [Brassicaceae](Isatidis Radix); Ophiopogon japonicus (Thunb.) Ker Gawl.(Radix Ophiopogonis); Houttuynia cordata Thunb. [Saururaceae](heartleaf houttuynia herb) | Y - Prepared according to People's Republic of China Pharmacopoeia | N |
| Han et al., 2016 | Xiaoer Feire Kechuan oral liquid | Heilongjiang sunflower Pharmaceutical Co., Ltd. (SFDA approval number: Z10950080) | Ephedra sinica Stapf [Ephedraceae](chinese ephedra); Corydalis bungeana Turcz. [Papaveraceae] (bunge corydalis herb); Gypsum (CaSO4·2H2O); Lonicera japonica Thunb. [Caprifoliaceae](Lonicera japonica); Forsythia suspensa (Thunb.) Vahl [Oleaceae](fructus forsythiae suspensae); Anemarrhena asphodeloides Bunge (Common Anemarrhena Rhizome); Scutellaria baicalensis Georgi [Lamiaceae](baical skullcap root); Isatis tinctoria L. [Brassicaceae](Isatidis Radix); Ophiopogon japonicus (Thunb.) Ker Gawl.(Radix Ophiopogonis); Houttuynia cordata Thunb. [Saururaceae](heartleaf houttuynia herb) | Y - Prepared according to People's Republic of China Pharmacopoeia | N |
| Jin, 2016 | Xiaoer Feire Kechuan oral liquid | Heilongjiang sunflower Pharmaceutical Co., Ltd. (SFDA approval number: Z10950080) | Ephedra sinica Stapf [Ephedraceae](chinese ephedra); Corydalis bungeana Turcz. [Papaveraceae] (bunge corydalis herb); Gypsum (CaSO4·2H2O); Lonicera japonica Thunb. [Caprifoliaceae](Lonicera japonica); Forsythia suspensa (Thunb.) Vahl [Oleaceae](fructus forsythiae suspensae); Anemarrhena asphodeloides Bunge (Common Anemarrhena Rhizome); Scutellaria baicalensis Georgi [Lamiaceae](baical skullcap root); Isatis tinctoria L. [Brassicaceae](Isatidis Radix); Ophiopogon japonicus (Thunb.) Ker Gawl.(Radix Ophiopogonis); Houttuynia cordata Thunb. [Saururaceae](heartleaf houttuynia herb) | Y - Prepared according to People's Republic of China Pharmacopoeia | N |
| Li, 2019 | Xiaoer Feire Kechuan oral liquid | Heilongjiang sunflower Pharmaceutical Co., Ltd. (SFDA approval number: Z10950080) | Ephedra sinica Stapf [Ephedraceae](chinese ephedra); Corydalis bungeana Turcz. [Papaveraceae] (bunge corydalis herb); Gypsum (CaSO4·2H2O); Lonicera japonica Thunb. [Caprifoliaceae](Lonicera japonica); Forsythia suspensa (Thunb.) Vahl [Oleaceae](fructus forsythiae suspensae); Anemarrhena asphodeloides Bunge (Common Anemarrhena Rhizome); Scutellaria baicalensis Georgi [Lamiaceae](baical skullcap root); Isatis tinctoria L. [Brassicaceae](Isatidis Radix); Ophiopogon japonicus (Thunb.) Ker Gawl.(Radix Ophiopogonis); Houttuynia cordata Thunb. [Saururaceae](heartleaf houttuynia herb) | Y - Prepared according to People's Republic of China Pharmacopoeia | N |
| Lin et al., 2019 | Xiaoer Feire Kechuan oral liquid | Heilongjiang sunflower Pharmaceutical Co., Ltd. (SFDA approval number: Z10950080) | Ephedra sinica Stapf [Ephedraceae](chinese ephedra); Corydalis bungeana Turcz. [Papaveraceae] (bunge corydalis herb); Gypsum (CaSO4·2H2O); Lonicera japonica Thunb. [Caprifoliaceae](Lonicera japonica); Forsythia suspensa (Thunb.) Vahl [Oleaceae](fructus forsythiae suspensae); Anemarrhena asphodeloides Bunge (Common Anemarrhena Rhizome); Scutellaria baicalensis Georgi [Lamiaceae](baical skullcap root); Isatis tinctoria L. [Brassicaceae](Isatidis Radix); Ophiopogon japonicus (Thunb.) Ker Gawl.(Radix Ophiopogonis); Houttuynia cordata Thunb. [Saururaceae](heartleaf houttuynia herb) | Y - Prepared according to People's Republic of China Pharmacopoeia | N |
| Liu, 2017 | Xiaoer Feire Kechuan oral liquid | Heilongjiang sunflower Pharmaceutical Co., Ltd. (SFDA approval number: Z10950080) | Ephedra sinica Stapf [Ephedraceae](chinese ephedra); Corydalis bungeana Turcz. [Papaveraceae] (bunge corydalis herb); Gypsum (CaSO4·2H2O); Lonicera japonica Thunb. [Caprifoliaceae](Lonicera japonica); Forsythia suspensa (Thunb.) Vahl [Oleaceae](fructus forsythiae suspensae); Anemarrhena asphodeloides Bunge (Common Anemarrhena Rhizome); Scutellaria baicalensis Georgi [Lamiaceae](baical skullcap root); Isatis tinctoria L. [Brassicaceae](Isatidis Radix); Ophiopogon japonicus (Thunb.) Ker Gawl.(Radix Ophiopogonis); Houttuynia cordata Thunb. [Saururaceae](heartleaf houttuynia herb) | Y - Prepared according to People's Republic of China Pharmacopoeia | N |
| Liu and Cao, 2017 | Xiaoer Feire Kechuan oral liquid | Heilongjiang sunflower Pharmaceutical Co., Ltd. (SFDA approval number: Z10950080) | Ephedra sinica Stapf [Ephedraceae](chinese ephedra); Corydalis bungeana Turcz. [Papaveraceae] (bunge corydalis herb); Gypsum (CaSO4·2H2O); Lonicera japonica Thunb. [Caprifoliaceae](Lonicera japonica); Forsythia suspensa (Thunb.) Vahl [Oleaceae](fructus forsythiae suspensae); Anemarrhena asphodeloides Bunge (Common Anemarrhena Rhizome); Scutellaria baicalensis Georgi [Lamiaceae](baical skullcap root); Isatis tinctoria L. [Brassicaceae](Isatidis Radix); Ophiopogon japonicus (Thunb.) Ker Gawl.(Radix Ophiopogonis); Houttuynia cordata Thunb. [Saururaceae](heartleaf houttuynia herb) | Y - Prepared according to People's Republic of China Pharmacopoeia | N |
| Luan et al., 2020 | Xiaoer Feire Kechuan oral liquid | Heilongjiang sunflower Pharmaceutical Co., Ltd. (SFDA approval number: Z10950080) | Ephedra sinica Stapf [Ephedraceae](chinese ephedra); Corydalis bungeana Turcz. [Papaveraceae] (bunge corydalis herb); Gypsum (CaSO4·2H2O); Lonicera japonica Thunb. [Caprifoliaceae](Lonicera japonica); Forsythia suspensa (Thunb.) Vahl [Oleaceae](fructus forsythiae suspensae); Anemarrhena asphodeloides Bunge (Common Anemarrhena Rhizome); Scutellaria baicalensis Georgi [Lamiaceae](baical skullcap root); Isatis tinctoria L. [Brassicaceae](Isatidis Radix); Ophiopogon japonicus (Thunb.) Ker Gawl.(Radix Ophiopogonis); Houttuynia cordata Thunb. [Saururaceae](heartleaf houttuynia herb) | Y - Prepared according to People's Republic of China Pharmacopoeia | N |
| Luo et al., 2017 | Xiaoer Feire Kechuan oral liquid | Heilongjiang sunflower Pharmaceutical Co., Ltd. (SFDA approval number: Z10950080) | Ephedra sinica Stapf [Ephedraceae](chinese ephedra); Corydalis bungeana Turcz. [Papaveraceae] (bunge corydalis herb); Gypsum (CaSO4·2H2O); Lonicera japonica Thunb. [Caprifoliaceae](Lonicera japonica); Forsythia suspensa (Thunb.) Vahl [Oleaceae](fructus forsythiae suspensae); Anemarrhena asphodeloides Bunge (Common Anemarrhena Rhizome); Scutellaria baicalensis Georgi [Lamiaceae](baical skullcap root); Isatis tinctoria L. [Brassicaceae](Isatidis Radix); Ophiopogon japonicus (Thunb.) Ker Gawl.(Radix Ophiopogonis); Houttuynia cordata Thunb. [Saururaceae](heartleaf houttuynia herb) | Y - Prepared according to People's Republic of China Pharmacopoeia | N |
| Ma et al., 2018 | Xiaoer Feire Kechuan oral liquid | Heilongjiang sunflower Pharmaceutical Co., Ltd. (SFDA approval number: Z10950080) | Ephedra sinica Stapf [Ephedraceae](chinese ephedra); Corydalis bungeana Turcz. [Papaveraceae] (bunge corydalis herb); Gypsum (CaSO4·2H2O); Lonicera japonica Thunb. [Caprifoliaceae](Lonicera japonica); Forsythia suspensa (Thunb.) Vahl [Oleaceae](fructus forsythiae suspensae); Anemarrhena asphodeloides Bunge (Common Anemarrhena Rhizome); Scutellaria baicalensis Georgi [Lamiaceae](baical skullcap root); Isatis tinctoria L. [Brassicaceae](Isatidis Radix); Ophiopogon japonicus (Thunb.) Ker Gawl.(Radix Ophiopogonis); Houttuynia cordata Thunb. [Saururaceae](heartleaf houttuynia herb) | Y - Prepared according to People's Republic of China Pharmacopoeia | N |
| Mei, 2016 | Xiaoer Feire Kechuan oral liquid | Heilongjiang sunflower Pharmaceutical Co., Ltd. (SFDA approval number: Z10950080) | Ephedra sinica Stapf [Ephedraceae](chinese ephedra); Corydalis bungeana Turcz. [Papaveraceae] (bunge corydalis herb); Gypsum (CaSO4·2H2O); Lonicera japonica Thunb. [Caprifoliaceae](Lonicera japonica); Forsythia suspensa (Thunb.) Vahl [Oleaceae](fructus forsythiae suspensae); Anemarrhena asphodeloides Bunge (Common Anemarrhena Rhizome); Scutellaria baicalensis Georgi [Lamiaceae](baical skullcap root); Isatis tinctoria L. [Brassicaceae](Isatidis Radix); Ophiopogon japonicus (Thunb.) Ker Gawl.(Radix Ophiopogonis); Houttuynia cordata Thunb. [Saururaceae](heartleaf houttuynia herb) | Y - Prepared according to People's Republic of China Pharmacopoeia | N |
| Meng, 2016 | Xiaoer Feire Kechuan oral liquid | Heilongjiang sunflower Pharmaceutical Co., Ltd. (SFDA approval number: Z10950080) | Ephedra sinica Stapf [Ephedraceae](chinese ephedra); Corydalis bungeana Turcz. [Papaveraceae] (bunge corydalis herb); Gypsum (CaSO4·2H2O); Lonicera japonica Thunb. [Caprifoliaceae](Lonicera japonica); Forsythia suspensa (Thunb.) Vahl [Oleaceae](fructus forsythiae suspensae); Anemarrhena asphodeloides Bunge (Common Anemarrhena Rhizome); Scutellaria baicalensis Georgi [Lamiaceae](baical skullcap root); Isatis tinctoria L. [Brassicaceae](Isatidis Radix); Ophiopogon japonicus (Thunb.) Ker Gawl.(Radix Ophiopogonis); Houttuynia cordata Thunb. [Saururaceae](heartleaf houttuynia herb) | Y - Prepared according to People's Republic of China Pharmacopoeia | N |
| Pang and Guo, 2007 | Xiaoer Feire Kechuan oral liquid | Heilongjiang sunflower Pharmaceutical Co., Ltd. (SFDA approval number: Z10950080) | Ephedra sinica Stapf [Ephedraceae](chinese ephedra); Corydalis bungeana Turcz. [Papaveraceae] (bunge corydalis herb); Gypsum (CaSO4·2H2O); Lonicera japonica Thunb. [Caprifoliaceae](Lonicera japonica); Forsythia suspensa (Thunb.) Vahl [Oleaceae](fructus forsythiae suspensae); Anemarrhena asphodeloides Bunge (Common Anemarrhena Rhizome); Scutellaria baicalensis Georgi [Lamiaceae](baical skullcap root); Isatis tinctoria L. [Brassicaceae](Isatidis Radix); Ophiopogon japonicus (Thunb.) Ker Gawl.(Radix Ophiopogonis); Houttuynia cordata Thunb. [Saururaceae](heartleaf houttuynia herb) | Y - Prepared according to People's Republic of China Pharmacopoeia | N |
| Song, 2019 | Xiaoer Feire Kechuan oral liquid | Heilongjiang sunflower Pharmaceutical Co., Ltd. (SFDA approval number: Z10950080) | Ephedra sinica Stapf [Ephedraceae](chinese ephedra); Corydalis bungeana Turcz. [Papaveraceae] (bunge corydalis herb); Gypsum (CaSO4·2H2O); Lonicera japonica Thunb. [Caprifoliaceae](Lonicera japonica); Forsythia suspensa (Thunb.) Vahl [Oleaceae](fructus forsythiae suspensae); Anemarrhena asphodeloides Bunge (Common Anemarrhena Rhizome); Scutellaria baicalensis Georgi [Lamiaceae](baical skullcap root); Isatis tinctoria L. [Brassicaceae](Isatidis Radix); Ophiopogon japonicus (Thunb.) Ker Gawl.(Radix Ophiopogonis); Houttuynia cordata Thunb. [Saururaceae](heartleaf houttuynia herb) | Y - Prepared according to People's Republic of China Pharmacopoeia | N |
| Tan, 2019 | Xiaoer Feire Kechuan oral liquid | Heilongjiang sunflower Pharmaceutical Co., Ltd. (SFDA approval number: Z10950080) | Ephedra sinica Stapf [Ephedraceae](chinese ephedra); Corydalis bungeana Turcz. [Papaveraceae] (bunge corydalis herb); Gypsum (CaSO4·2H2O); Lonicera japonica Thunb. [Caprifoliaceae](Lonicera japonica); Forsythia suspensa (Thunb.) Vahl [Oleaceae](fructus forsythiae suspensae); Anemarrhena asphodeloides Bunge (Common Anemarrhena Rhizome); Scutellaria baicalensis Georgi [Lamiaceae](baical skullcap root); Isatis tinctoria L. [Brassicaceae](Isatidis Radix); Ophiopogon japonicus (Thunb.) Ker Gawl.(Radix Ophiopogonis); Houttuynia cordata Thunb. [Saururaceae](heartleaf houttuynia herb) | Y - Prepared according to People's Republic of China Pharmacopoeia | N |
| Wang, 2019a | Xiaoer Feire Kechuan oral liquid | Heilongjiang sunflower Pharmaceutical Co., Ltd. (SFDA approval number: Z10950080) | Ephedra sinica Stapf [Ephedraceae](chinese ephedra); Corydalis bungeana Turcz. [Papaveraceae] (bunge corydalis herb); Gypsum (CaSO4·2H2O); Lonicera japonica Thunb. [Caprifoliaceae](Lonicera japonica); Forsythia suspensa (Thunb.) Vahl [Oleaceae](fructus forsythiae suspensae); Anemarrhena asphodeloides Bunge (Common Anemarrhena Rhizome); Scutellaria baicalensis Georgi [Lamiaceae](baical skullcap root); Isatis tinctoria L. [Brassicaceae](Isatidis Radix); Ophiopogon japonicus (Thunb.) Ker Gawl.(Radix Ophiopogonis); Houttuynia cordata Thunb. [Saururaceae](heartleaf houttuynia herb) | Y - Prepared according to People's Republic of China Pharmacopoeia | N |
| Wang, 2019b | Xiaoer Feire Kechuan oral liquid | Heilongjiang sunflower Pharmaceutical Co., Ltd. (SFDA approval number: Z10950080) | Ephedra sinica Stapf [Ephedraceae](chinese ephedra); Corydalis bungeana Turcz. [Papaveraceae] (bunge corydalis herb); Gypsum (CaSO4·2H2O); Lonicera japonica Thunb. [Caprifoliaceae](Lonicera japonica); Forsythia suspensa (Thunb.) Vahl [Oleaceae](fructus forsythiae suspensae); Anemarrhena asphodeloides Bunge (Common Anemarrhena Rhizome); Scutellaria baicalensis Georgi [Lamiaceae](baical skullcap root); Isatis tinctoria L. [Brassicaceae](Isatidis Radix); Ophiopogon japonicus (Thunb.) Ker Gawl.(Radix Ophiopogonis); Houttuynia cordata Thunb. [Saururaceae](heartleaf houttuynia herb) | Y - Prepared according to People's Republic of China Pharmacopoeia | N |
| Xiong, 2018 | Xiaoer Feire Kechuan oral liquid | Heilongjiang sunflower Pharmaceutical Co., Ltd. (SFDA approval number: Z10950080) | Ephedra sinica Stapf [Ephedraceae](chinese ephedra); Corydalis bungeana Turcz. [Papaveraceae] (bunge corydalis herb); Gypsum (CaSO4·2H2O); Lonicera japonica Thunb. [Caprifoliaceae](Lonicera japonica); Forsythia suspensa (Thunb.) Vahl [Oleaceae](fructus forsythiae suspensae); Anemarrhena asphodeloides Bunge (Common Anemarrhena Rhizome); Scutellaria baicalensis Georgi [Lamiaceae](baical skullcap root); Isatis tinctoria L. [Brassicaceae](Isatidis Radix); Ophiopogon japonicus (Thunb.) Ker Gawl.(Radix Ophiopogonis); Houttuynia cordata Thunb. [Saururaceae](heartleaf houttuynia herb) | Y - Prepared according to People's Republic of China Pharmacopoeia | N |
| Xu, 2016 | Xiaoer Feire Kechuan oral liquid | Heilongjiang sunflower Pharmaceutical Co., Ltd. (SFDA approval number: Z10950080) | Ephedra sinica Stapf [Ephedraceae](chinese ephedra); Corydalis bungeana Turcz. [Papaveraceae] (bunge corydalis herb); Gypsum (CaSO4·2H2O); Lonicera japonica Thunb. [Caprifoliaceae](Lonicera japonica); Forsythia suspensa (Thunb.) Vahl [Oleaceae](fructus forsythiae suspensae); Anemarrhena asphodeloides Bunge (Common Anemarrhena Rhizome); Scutellaria baicalensis Georgi [Lamiaceae](baical skullcap root); Isatis tinctoria L. [Brassicaceae](Isatidis Radix); Ophiopogon japonicus (Thunb.) Ker Gawl.(Radix Ophiopogonis); Houttuynia cordata Thunb. [Saururaceae](heartleaf houttuynia herb) | Y - Prepared according to People's Republic of China Pharmacopoeia | N |
| Yang, 2018 | Xiaoer Feire Kechuan oral liquid | Heilongjiang sunflower Pharmaceutical Co., Ltd. (SFDA approval number: Z10950080) | Ephedra sinica Stapf [Ephedraceae](chinese ephedra); Corydalis bungeana Turcz. [Papaveraceae] (bunge corydalis herb); Gypsum (CaSO4·2H2O); Lonicera japonica Thunb. [Caprifoliaceae](Lonicera japonica); Forsythia suspensa (Thunb.) Vahl [Oleaceae](fructus forsythiae suspensae); Anemarrhena asphodeloides Bunge (Common Anemarrhena Rhizome); Scutellaria baicalensis Georgi [Lamiaceae](baical skullcap root); Isatis tinctoria L. [Brassicaceae](Isatidis Radix); Ophiopogon japonicus (Thunb.) Ker Gawl.(Radix Ophiopogonis); Houttuynia cordata Thunb. [Saururaceae](heartleaf houttuynia herb) | Y - Prepared according to People's Republic of China Pharmacopoeia | N |
| Yang et al., 2020 | Xiaoer Feire Kechuan oral liquid | Heilongjiang sunflower Pharmaceutical Co., Ltd. (SFDA approval number: Z10950080) | Ephedra sinica Stapf [Ephedraceae](chinese ephedra); Corydalis bungeana Turcz. [Papaveraceae] (bunge corydalis herb); Gypsum (CaSO4·2H2O); Lonicera japonica Thunb. [Caprifoliaceae](Lonicera japonica); Forsythia suspensa (Thunb.) Vahl [Oleaceae](fructus forsythiae suspensae); Anemarrhena asphodeloides Bunge (Common Anemarrhena Rhizome); Scutellaria baicalensis Georgi [Lamiaceae](baical skullcap root); Isatis tinctoria L. [Brassicaceae](Isatidis Radix); Ophiopogon japonicus (Thunb.) Ker Gawl.(Radix Ophiopogonis); Houttuynia cordata Thunb. [Saururaceae](heartleaf houttuynia herb) | Y - Prepared according to People's Republic of China Pharmacopoeia | N |
| Yu and Yuan, 2016 | Xiaoer Feire Kechuan oral liquid | Heilongjiang sunflower Pharmaceutical Co., Ltd. (SFDA approval number: Z10950080) | Ephedra sinica Stapf [Ephedraceae](chinese ephedra); Corydalis bungeana Turcz. [Papaveraceae] (bunge corydalis herb); Gypsum (CaSO4·2H2O); Lonicera japonica Thunb. [Caprifoliaceae](Lonicera japonica); Forsythia suspensa (Thunb.) Vahl [Oleaceae](fructus forsythiae suspensae); Anemarrhena asphodeloides Bunge (Common Anemarrhena Rhizome); Scutellaria baicalensis Georgi [Lamiaceae](baical skullcap root); Isatis tinctoria L. [Brassicaceae](Isatidis Radix); Ophiopogon japonicus (Thunb.) Ker Gawl.(Radix Ophiopogonis); Houttuynia cordata Thunb. [Saururaceae](heartleaf houttuynia herb) | Y - Prepared according to People's Republic of China Pharmacopoeia | N |
| Zhu, 2019 | Xiaoer Feire Kechuan oral liquid | Heilongjiang sunflower Pharmaceutical Co., Ltd. (SFDA approval number: Z10950080) | Ephedra sinica Stapf [Ephedraceae](chinese ephedra); Corydalis bungeana Turcz. [Papaveraceae] (bunge corydalis herb); Gypsum (CaSO4·2H2O); Lonicera japonica Thunb. [Caprifoliaceae](Lonicera japonica); Forsythia suspensa (Thunb.) Vahl [Oleaceae](fructus forsythiae suspensae); Anemarrhena asphodeloides Bunge (Common Anemarrhena Rhizome); Scutellaria baicalensis Georgi [Lamiaceae](baical skullcap root); Isatis tinctoria L. [Brassicaceae](Isatidis Radix); Ophiopogon japonicus (Thunb.) Ker Gawl.(Radix Ophiopogonis); Houttuynia cordata Thunb. [Saururaceae](heartleaf houttuynia herb) | Y - Prepared according to People's Republic of China Pharmacopoeia | N |
| Zhu et al., 2017 | Xiaoer Feire Kechuan oral liquid | Heilongjiang sunflower Pharmaceutical Co., Ltd. (SFDA approval number: Z10950080) | Ephedra sinica Stapf [Ephedraceae](chinese ephedra); Corydalis bungeana Turcz. [Papaveraceae] (bunge corydalis herb); Gypsum (CaSO4·2H2O); Lonicera japonica Thunb. [Caprifoliaceae](Lonicera japonica); Forsythia suspensa (Thunb.) Vahl [Oleaceae](fructus forsythiae suspensae); Anemarrhena asphodeloides Bunge (Common Anemarrhena Rhizome); Scutellaria baicalensis Georgi [Lamiaceae](baical skullcap root); Isatis tinctoria L. [Brassicaceae](Isatidis Radix); Ophiopogon japonicus (Thunb.) Ker Gawl.(Radix Ophiopogonis); Houttuynia cordata Thunb. [Saururaceae](heartleaf houttuynia herb) | Y - Prepared according to People's Republic of China Pharmacopoeia | N |
| Chao and Huang, 2019 | Xiaoer Xiaoji Zhike oral liquid | Lunan Houpu Pharmaceutical Co., Ltd. (SFDA approval number: Z10970022) | Crataegus pinnatifida Bunge [Rosaceae](chinese hawthorn); Areca catechu L. [Arecaceae](areca nut); Citrus × aurantium L. [Rutaceae](aurantii fructus immaturus); Eriobotrya japonica (Thunb.) Lindl. [Rosaceae](eriobotryae folium); Trichosanthes kirilowii Maxim.(fructus trichosanthis); Raphanus raphanistrum subsp. sativus (L.) Domin [Brassicaceae](semen raphani); Descurainia sophia (L.) Webb ex Prantl [Brassicaceae](semen lepidii ); Platycodon grandiflorus (Jacq.) A.DC. [Campanulaceae](balloon flower root); Forsythia suspensa (Thunb.) Vahl [Oleaceae](fructus forsythiae suspensae); Cicadae Periostracum | Y - Prepared according to People's Republic of China Pharmacopoeia | N |
| Chen, 2018 | Xiaoer Xiaoji Zhike oral liquid | Lunan Houpu Pharmaceutical Co., Ltd. (SFDA approval number: Z10970022) | Crataegus pinnatifida Bunge [Rosaceae](chinese hawthorn); Areca catechu L. [Arecaceae](areca nut); Citrus × aurantium L. [Rutaceae](aurantii fructus immaturus); Eriobotrya japonica (Thunb.) Lindl. [Rosaceae](eriobotryae folium); Trichosanthes kirilowii Maxim.(fructus trichosanthis); Raphanus raphanistrum subsp. sativus (L.) Domin [Brassicaceae](semen raphani); Descurainia sophia (L.) Webb ex Prantl [Brassicaceae](semen lepidii ); Platycodon grandiflorus (Jacq.) A.DC. [Campanulaceae](balloon flower root); Forsythia suspensa (Thunb.) Vahl [Oleaceae](fructus forsythiae suspensae); Cicadae Periostracum | Y - Prepared according to People's Republic of China Pharmacopoeia | N |
| Hu, 2018 | Xiaoer Xiaoji Zhike oral liquid | Lunan Houpu Pharmaceutical Co., Ltd. (SFDA approval number: Z10970022) | Crataegus pinnatifida Bunge [Rosaceae](chinese hawthorn); Areca catechu L. [Arecaceae](areca nut); Citrus × aurantium L. [Rutaceae](aurantii fructus immaturus); Eriobotrya japonica (Thunb.) Lindl. [Rosaceae](eriobotryae folium); Trichosanthes kirilowii Maxim.(fructus trichosanthis); Raphanus raphanistrum subsp. sativus (L.) Domin [Brassicaceae](semen raphani); Descurainia sophia (L.) Webb ex Prantl [Brassicaceae](semen lepidii ); Platycodon grandiflorus (Jacq.) A.DC. [Campanulaceae](balloon flower root); Forsythia suspensa (Thunb.) Vahl [Oleaceae](fructus forsythiae suspensae); Cicadae Periostracum | Y - Prepared according to People's Republic of China Pharmacopoeia | N |
| Huang, 2019 | Xiaoer Xiaoji Zhike oral liquid | Lunan Houpu Pharmaceutical Co., Ltd. (SFDA approval number: Z10970022) | Crataegus pinnatifida Bunge [Rosaceae](chinese hawthorn); Areca catechu L. [Arecaceae](areca nut); Citrus × aurantium L. [Rutaceae](aurantii fructus immaturus); Eriobotrya japonica (Thunb.) Lindl. [Rosaceae](eriobotryae folium); Trichosanthes kirilowii Maxim.(fructus trichosanthis); Raphanus raphanistrum subsp. sativus (L.) Domin [Brassicaceae](semen raphani); Descurainia sophia (L.) Webb ex Prantl [Brassicaceae](semen lepidii ); Platycodon grandiflorus (Jacq.) A.DC. [Campanulaceae](balloon flower root); Forsythia suspensa (Thunb.) Vahl [Oleaceae](fructus forsythiae suspensae); Cicadae Periostracum | Y - Prepared according to People's Republic of China Pharmacopoeia | N |
| Li, 2018 | Xiaoer Xiaoji Zhike oral liquid | Lunan Houpu Pharmaceutical Co., Ltd. (SFDA approval number: Z10970022) | Crataegus pinnatifida Bunge [Rosaceae](chinese hawthorn); Areca catechu L. [Arecaceae](areca nut); Citrus × aurantium L. [Rutaceae](aurantii fructus immaturus); Eriobotrya japonica (Thunb.) Lindl. [Rosaceae](eriobotryae folium); Trichosanthes kirilowii Maxim.(fructus trichosanthis); Raphanus raphanistrum subsp. sativus (L.) Domin [Brassicaceae](semen raphani); Descurainia sophia (L.) Webb ex Prantl [Brassicaceae](semen lepidii ); Platycodon grandiflorus (Jacq.) A.DC. [Campanulaceae](balloon flower root); Forsythia suspensa (Thunb.) Vahl [Oleaceae](fructus forsythiae suspensae); Cicadae Periostracum | Y - Prepared according to People's Republic of China Pharmacopoeia | N |
| Li and Jin 2018 | Xiaoer Xiaoji Zhike oral liquid | Lunan Houpu Pharmaceutical Co., Ltd. (SFDA approval number: Z10970022) | Crataegus pinnatifida Bunge [Rosaceae](chinese hawthorn); Areca catechu L. [Arecaceae](areca nut); Citrus × aurantium L. [Rutaceae](aurantii fructus immaturus); Eriobotrya japonica (Thunb.) Lindl. [Rosaceae](eriobotryae folium); Trichosanthes kirilowii Maxim.(fructus trichosanthis); Raphanus raphanistrum subsp. sativus (L.) Domin [Brassicaceae](semen raphani); Descurainia sophia (L.) Webb ex Prantl [Brassicaceae](semen lepidii ); Platycodon grandiflorus (Jacq.) A.DC. [Campanulaceae](balloon flower root); Forsythia suspensa (Thunb.) Vahl [Oleaceae](fructus forsythiae suspensae); Cicadae Periostracum | Y - Prepared according to People's Republic of China Pharmacopoeia | N |
| Wang, 2016 | Xiaoer Xiaoji Zhike oral liquid | Lunan Houpu Pharmaceutical Co., Ltd. (SFDA approval number: Z10970022) | Crataegus pinnatifida Bunge [Rosaceae](chinese hawthorn); Areca catechu L. [Arecaceae](areca nut); Citrus × aurantium L. [Rutaceae](aurantii fructus immaturus); Eriobotrya japonica (Thunb.) Lindl. [Rosaceae](eriobotryae folium); Trichosanthes kirilowii Maxim.(fructus trichosanthis); Raphanus raphanistrum subsp. sativus (L.) Domin [Brassicaceae](semen raphani); Descurainia sophia (L.) Webb ex Prantl [Brassicaceae](semen lepidii ); Platycodon grandiflorus (Jacq.) A.DC. [Campanulaceae](balloon flower root); Forsythia suspensa (Thunb.) Vahl [Oleaceae](fructus forsythiae suspensae); Cicadae Periostracum | Y - Prepared according to People's Republic of China Pharmacopoeia | N |
| Wang and Wang, 2019 | Xiaoer Xiaoji Zhike oral liquid | Lunan Houpu Pharmaceutical Co., Ltd. (SFDA approval number: Z10970022) | Crataegus pinnatifida Bunge [Rosaceae](chinese hawthorn); Areca catechu L. [Arecaceae](areca nut); Citrus × aurantium L. [Rutaceae](aurantii fructus immaturus); Eriobotrya japonica (Thunb.) Lindl. [Rosaceae](eriobotryae folium); Trichosanthes kirilowii Maxim.(fructus trichosanthis); Raphanus raphanistrum subsp. sativus (L.) Domin [Brassicaceae](semen raphani); Descurainia sophia (L.) Webb ex Prantl [Brassicaceae](semen lepidii ); Platycodon grandiflorus (Jacq.) A.DC. [Campanulaceae](balloon flower root); Forsythia suspensa (Thunb.) Vahl [Oleaceae](fructus forsythiae suspensae); Cicadae Periostracum | Y - Prepared according to People's Republic of China Pharmacopoeia | N |
| Xie, 2015 | Xiaoer Xiaoji Zhike oral liquid | Lunan Houpu Pharmaceutical Co., Ltd. (SFDA approval number: Z10970022) | Crataegus pinnatifida Bunge [Rosaceae](chinese hawthorn); Areca catechu L. [Arecaceae](areca nut); Citrus × aurantium L. [Rutaceae](aurantii fructus immaturus); Eriobotrya japonica (Thunb.) Lindl. [Rosaceae](eriobotryae folium); Trichosanthes kirilowii Maxim.(fructus trichosanthis); Raphanus raphanistrum subsp. sativus (L.) Domin [Brassicaceae](semen raphani); Descurainia sophia (L.) Webb ex Prantl [Brassicaceae](semen lepidii ); Platycodon grandiflorus (Jacq.) A.DC. [Campanulaceae](balloon flower root); Forsythia suspensa (Thunb.) Vahl [Oleaceae](fructus forsythiae suspensae); Cicadae Periostracum | Y - Prepared according to People's Republic of China Pharmacopoeia | N |
| Xue, 2020 | Xiaoer Xiaoji Zhike oral liquid | Lunan Houpu Pharmaceutical Co., Ltd. (SFDA approval number: Z10970022) | Crataegus pinnatifida Bunge [Rosaceae](chinese hawthorn); Areca catechu L. [Arecaceae](areca nut); Citrus × aurantium L. [Rutaceae](aurantii fructus immaturus); Eriobotrya japonica (Thunb.) Lindl. [Rosaceae](eriobotryae folium); Trichosanthes kirilowii Maxim.(fructus trichosanthis); Raphanus raphanistrum subsp. sativus (L.) Domin [Brassicaceae](semen raphani); Descurainia sophia (L.) Webb ex Prantl [Brassicaceae](semen lepidii ); Platycodon grandiflorus (Jacq.) A.DC. [Campanulaceae](balloon flower root); Forsythia suspensa (Thunb.) Vahl [Oleaceae](fructus forsythiae suspensae); Cicadae Periostracum | Y - Prepared according to People's Republic of China Pharmacopoeia | N |
| Yang, 2019 | Xiaoer Xiaoji Zhike oral liquid | Lunan Houpu Pharmaceutical Co., Ltd. (SFDA approval number: Z10970022) | Crataegus pinnatifida Bunge [Rosaceae](chinese hawthorn); Areca catechu L. [Arecaceae](areca nut); Citrus × aurantium L. [Rutaceae](aurantii fructus immaturus); Eriobotrya japonica (Thunb.) Lindl. [Rosaceae](eriobotryae folium); Trichosanthes kirilowii Maxim.(fructus trichosanthis); Raphanus raphanistrum subsp. sativus (L.) Domin [Brassicaceae](semen raphani); Descurainia sophia (L.) Webb ex Prantl [Brassicaceae](semen lepidii ); Platycodon grandiflorus (Jacq.) A.DC. [Campanulaceae](balloon flower root); Forsythia suspensa (Thunb.) Vahl [Oleaceae](fructus forsythiae suspensae); Cicadae Periostracum | Y - Prepared according to People's Republic of China Pharmacopoeia | N |
| Ye and Chen, 2018 | Xiaoer Xiaoji Zhike oral liquid | Lunan Houpu Pharmaceutical Co., Ltd. (SFDA approval number: Z10970022) | Crataegus pinnatifida Bunge [Rosaceae](chinese hawthorn); Areca catechu L. [Arecaceae](areca nut); Citrus × aurantium L. [Rutaceae](aurantii fructus immaturus); Eriobotrya japonica (Thunb.) Lindl. [Rosaceae](eriobotryae folium); Trichosanthes kirilowii Maxim.(fructus trichosanthis); Raphanus raphanistrum subsp. sativus (L.) Domin [Brassicaceae](semen raphani); Descurainia sophia (L.) Webb ex Prantl [Brassicaceae](semen lepidii ); Platycodon grandiflorus (Jacq.) A.DC. [Campanulaceae](balloon flower root); Forsythia suspensa (Thunb.) Vahl [Oleaceae](fructus forsythiae suspensae); Cicadae Periostracum | Y - Prepared according to People's Republic of China Pharmacopoeia | N |
| Yu, 2017 | Xiaoer Xiaoji Zhike oral liquid | Lunan Houpu Pharmaceutical Co., Ltd. (SFDA approval number: Z10970022) | Crataegus pinnatifida Bunge [Rosaceae](chinese hawthorn); Areca catechu L. [Arecaceae](areca nut); Citrus × aurantium L. [Rutaceae](aurantii fructus immaturus); Eriobotrya japonica (Thunb.) Lindl. [Rosaceae](eriobotryae folium); Trichosanthes kirilowii Maxim.(fructus trichosanthis); Raphanus raphanistrum subsp. sativus (L.) Domin [Brassicaceae](semen raphani); Descurainia sophia (L.) Webb ex Prantl [Brassicaceae](semen lepidii ); Platycodon grandiflorus (Jacq.) A.DC. [Campanulaceae](balloon flower root); Forsythia suspensa (Thunb.) Vahl [Oleaceae](fructus forsythiae suspensae); Cicadae Periostracum | Y - Prepared according to People's Republic of China Pharmacopoeia | N |
| Yuan and Wang, 2015 | Xiaoer Xiaoji Zhike oral liquid | Lunan Houpu Pharmaceutical Co., Ltd. (SFDA approval number: Z10970022) | Crataegus pinnatifida Bunge [Rosaceae](chinese hawthorn); Areca catechu L. [Arecaceae](areca nut); Citrus × aurantium L. [Rutaceae](aurantii fructus immaturus); Eriobotrya japonica (Thunb.) Lindl. [Rosaceae](eriobotryae folium); Trichosanthes kirilowii Maxim.(fructus trichosanthis); Raphanus raphanistrum subsp. sativus (L.) Domin [Brassicaceae](semen raphani); Descurainia sophia (L.) Webb ex Prantl [Brassicaceae](semen lepidii ); Platycodon grandiflorus (Jacq.) A.DC. [Campanulaceae](balloon flower root); Forsythia suspensa (Thunb.) Vahl [Oleaceae](fructus forsythiae suspensae); Cicadae Periostracum | Y - Prepared according to People's Republic of China Pharmacopoeia | N |
| Zhao, 2019 | Xiaoer Xiaoji Zhike oral liquid | Lunan Houpu Pharmaceutical Co., Ltd. (SFDA approval number: Z10970022) | Crataegus pinnatifida Bunge [Rosaceae](chinese hawthorn); Areca catechu L. [Arecaceae](areca nut); Citrus × aurantium L. [Rutaceae](aurantii fructus immaturus); Eriobotrya japonica (Thunb.) Lindl. [Rosaceae](eriobotryae folium); Trichosanthes kirilowii Maxim.(fructus trichosanthis); Raphanus raphanistrum subsp. sativus (L.) Domin [Brassicaceae](semen raphani); Descurainia sophia (L.) Webb ex Prantl [Brassicaceae](semen lepidii ); Platycodon grandiflorus (Jacq.) A.DC. [Campanulaceae](balloon flower root); Forsythia suspensa (Thunb.) Vahl [Oleaceae](fructus forsythiae suspensae); Cicadae Periostracum | Y - Prepared according to People's Republic of China Pharmacopoeia | N |
| Zheng et al., 2018 | Xiaoer Xiaoji Zhike oral liquid | Lunan Houpu Pharmaceutical Co., Ltd. (SFDA approval number: Z10970022) | Crataegus pinnatifida Bunge [Rosaceae](chinese hawthorn); Areca catechu L. [Arecaceae](areca nut); Citrus × aurantium L. [Rutaceae](aurantii fructus immaturus); Eriobotrya japonica (Thunb.) Lindl. [Rosaceae](eriobotryae folium); Trichosanthes kirilowii Maxim.(fructus trichosanthis); Raphanus raphanistrum subsp. sativus (L.) Domin [Brassicaceae](semen raphani); Descurainia sophia (L.) Webb ex Prantl [Brassicaceae](semen lepidii ); Platycodon grandiflorus (Jacq.) A.DC. [Campanulaceae](balloon flower root); Forsythia suspensa (Thunb.) Vahl [Oleaceae](fructus forsythiae suspensae); Cicadae Periostracum | Y - Prepared according to People's Republic of China Pharmacopoeia | N |
| Zong, 2019 | Xiaoer Xiaoji Zhike oral liquid | Lunan Houpu Pharmaceutical Co., Ltd. (SFDA approval number: Z10970022) | Crataegus pinnatifida Bunge [Rosaceae](chinese hawthorn); Areca catechu L. [Arecaceae](areca nut); Citrus × aurantium L. [Rutaceae](aurantii fructus immaturus); Eriobotrya japonica (Thunb.) Lindl. [Rosaceae](eriobotryae folium); Trichosanthes kirilowii Maxim.(fructus trichosanthis); Raphanus raphanistrum subsp. sativus (L.) Domin [Brassicaceae](semen raphani); Descurainia sophia (L.) Webb ex Prantl [Brassicaceae](semen lepidii ); Platycodon grandiflorus (Jacq.) A.DC. [Campanulaceae](balloon flower root); Forsythia suspensa (Thunb.) Vahl [Oleaceae](fructus forsythiae suspensae); Cicadae Periostracum | Y - Prepared according to People's Republic of China Pharmacopoeia | N |

SFDA: State Food and Drug Administration

# Table 2 Detailed Chemical Characterizations of Traditional Chinese Medicine Oral Liquids

| **Traditional Chinese medicine oral liquids*** | **Main chemical characterization** | **Molecular Formula** | **Median of test range (mg/ml)** |
| --- | --- | --- | --- |
| Pudilan Xiaoyan oral liquid | Baicalin | C21H18O11 | 9.6671 |
|  | Cichoric acid | C22H18O12 | 1.7853 |
|  | Adenosine | C10H13N5O4 | 0.0557 |
|  | Corynoline | C21H21NO5 | 0.0818 |
|  | Wogonin | C16H12O5 | 0.0339 |
|  | Chlorogenic acid | C16H18O9 | 0.0887 |
| Shuanghuanglian oral liquid | Chlorogenic acid | C16H18O9 | 0.566 |
|  | Forsythin | C27H34O11 | 0.6715 |
|  | Baicalin | C21H18O11 | 1.645 |
| Xiaoer Feire Kechuan oral liquid | Chlorogenic acid | C16H18O9 | 0.086 |
|  | Forsythin | C27H34O11 | 0.101 |
|  | Baicalin | C21H18O11 | 0.2 |
|  | (1R,2S)-(-)-EPHEDRINE HYDROCHLORIDE | C10H15NO·HCl | 0.1 |
|  | Forsythoside B | C34H44O19 | 0.105 |
|  | Liquiritin | C21H22O9 | 0.108 |
|  | Luteoloside | C21H20O11 | 0.0873 |
|  | Glycyrrhizic acid | C42H62O16 | 0.857 |
| Xiaoer Xiaoji Zhike oral liquid | synephrine | C9H13NO2 | 2.54 |
|  | Chlorogenic acid | C16H18O9 | 0.158 |
|  | quercetin3-O-gentobioside | C27H30O17 | 0.147 |
|  | Hyperoside | C21H20O12 | 0.034 |
|  | Forsythoside A | C29H36O15 | 0.432 |
|  | Naringin | C27H32O14 | 1.813 |
|  | hesperidin | C28H34O15 | 1.339 |
|  | neohesperidin | C28H34O15 | 2.58 |
|  | Forsythin | C27H34O11 | 0.175 |
|  | 3,29-Dibenzoyl rarounitriol | C44H58O5 | 1.615 |

***Reference**

Chen, J.,Li, B. Q.,Zhai, H. L.,Lü, W. J. and Zhang, X. Y. (2014). A practical application of wavelet moment method on the quantitative analysis of Shuanghuanglian oral liquid based on three-dimensional fingerprint spectra. J Chromatogr A. 1352, 55-61. doi:10.1016/j.chroma.2014.05.051

Dong, Z. B.,Li, C. and Shao, J. G. (2015). Simultaneous determination of seven components in Pudilan Xiaoyan oral liquid by HPLC. Zhongguo Zhong Yao Za Zhi. 40, 1747-1750

Liang, Y.,Zhang, G. M.,Su, R. Q.,Cheng, Y. L.,Zhao, H. Q. and Wang, X. (2017). Determination of chemical constituents and quantitative fingerprints of Xiao’er Xiaoji Zhike Oral Liquid by HPLC-ESI-Q-TOF/MS. Chinese Traditional and Herbal Drugs. 48, 4440-4447

Yu, Y. M.,Mou, N. and Zhang, P. (2013). Simultaneous determination of nine constituents in Xiao'er Feire Kechuan Liquid by HPLC-DAD. Chinese Traditional Patent Medicine. 35, 709-713

# Table 3 Characteristics of Included Studies

| ID | Sample | | Man | | Woman | | Age (years) | | Disease course (days) | | treatment course (days) | Intervention | | dosage | | Outcomes |
| --- | --- | --- | --- | --- | --- | --- | --- | --- | --- | --- | --- | --- | --- | --- | --- | --- |
|  | T | C | T | C | T | C | T | C | T | C |  | T | C | T | C |  |
| Fan 2017 | 58 | 58 | 33 | 35 | 25 | 23 | 4.9±1.4 | 4.7±1.2 | 1.21±0.22 | 1.18±0.21 | 15 | Shuanghuanglian oral liquid+azithromycin | azithromycin | Shuanghuanglian oral liquid (1-3age:10ml,po,tid; 4-7age:20ml,po,tid)+ azithromycin (sequential therapy:15mg/(kg·d),ivgtt,qd and 10~12mg/(kg·d),po,qd) | azithromycin (sequential therapy:15mg/(kg·d),ivgtt,qd and 10~12mg/(kg·d),po,qd) | ①⑦⑧⑩ |
| Guo 2017 | 60 | 60 | 35 | 37 | 25 | 23 | 5.2±0.8 | 4.8±1 | 3.8±0.5 | 4.1±0.6 | 14 | Shuanghuanglian oral liquid+azithromycin | azithromycin | Shuanghuanglian oral liquid (1-3age:10ml,po,tid; 4-7age:20ml,po,tid)+ azithromycin (8mg/(kg·d),po,qd) | azithromycin (8mg/(kg·d),po,qd) | ①⑩ |
| Liu 2019 | 60 | 60 | 34 | 38 | 26 | 22 | 6.58±2.72 | 6.48±2.25 | 2.51±0.94 | 2.43±0.89 | 14 | Shuanghuanglian oral liquid+azithromycin | azithromycin | Shuanghuanglian oral liquid (1-3age:10ml,po,tid; 4-7age:20ml,po,tid)+ azithromycin (sequential therapy:1mg/(kg·d),ivgtt,qd and 1mg/(kg·d),po,qd) | azithromycin (sequential therapy:1mg/(kg·d),ivgtt,qd and 1mg/(kg·d),po,qd) | ①②③④⑤⑨ |
| Liu 2016 | 50 | 50 | 28 | 27 | 22 | 23 | 5.9±1.3 | 6.1±1.4 | - | - | 14 | Shuanghuanglian oral liquid+azithromycin | azithromycin | Shuanghuanglian oral liquid (1-3age:10ml,po,tid; 4-7age:20ml,po,bid or tid)+ azithromycin (sequential therapy:10mg/(kg·d),ivgtt,qd and 10mg/(kg·d),po,qd) | azithromycin (sequential therapy:10mg/(kg·d),ivgtt,qd and 10mg/(kg·d),po,qd) | ①⑦⑧⑩ |
| Liu 2018 | 37 | 37 | 15 | 14 | 22 | 23 | 5.4±1.2 | 5.7±1.1 | - | - | 32 | Shuanghuanglian oral liquid+azithromycin | azithromycin | Shuanghuanglian oral liquid (1-3age:10ml,po,tid; 4-10age:20ml,po,tid)+ azithromycin (sequential therapy:1-2mg/ml,ivgtt,qd and 10mg/(kg·d),po,qd) | azithromycin (sequential therapy:1-2mg/ml,ivgtt,qd and 10mg/(kg·d),po,qd) | ①②③④⑦⑧⑩ |
| Shi 2020 | 39 | 39 | 21 | 20 | 18 | 19 | 4.33±1.15 | 4.28±1.09 | 3.28±0.61 | 3.53±0.59 | 10 | Shuanghuanglian oral liquid+azithromycin | azithromycin | Shuanghuanglian oral liquid (1-3age:10ml,po,tid; 4-7age:20ml,po,tid)+ azithromycin (sequential therapy:10mg/(kg·d),ivgtt,qd and 10mg/(kg·d),po,qd) | azithromycin (sequential therapy:10mg/(kg·d),ivgtt,qd and 10mg/(kg·d),po,qd) | ①②③④ |
| Wang 2018a | 55 | 55 | 35 | 33 | 20 | 22 | 3.73±1.38 | 3.38±1.15 | - | - | 14 | Shuanghuanglian oral liquid+azithromycin | azithromycin | Shuanghuanglian oral liquid (1-3age:10ml,po,tid; 4-7age:20ml,po,tid)+ azithromycin (sequential therapy:10mg/(kg·d),ivgtt,qd and 10mg/(kg·d),po,qd) | azithromycin (sequential therapy:10mg/(kg·d),ivgtt,qd and 10mg/(kg·d),po,qd) | ①②③④⑩ |
| Wang 2018b | 86 | 86 | 45 | 47 | 41 | 39 | 6.73±1.86 | 6.82±1.77 | 4.67±0.57 | 4.52±0.62 | 28 | Shuanghuanglian oral liquid+azithromycin | azithromycin | Shuanghuanglian oral liquid (20ml,po,tid)+ azithromycin (sequential therapy:10mg/(kg·d),ivgtt,qd and 10mg/(kg·d),po,qd) | azithromycin (sequential therapy:10mg/(kg·d),ivgtt,qd and 10mg/(kg·d),po,qd) | ①⑦⑧⑩ |
| Yao 2018 | 34 | 34 | 20 | 18 | 14 | 16 | 4.57±1.45 | 3.98±1.63 | 2.75±1.42 | 3.23±1.34 | 14 | Shuanghuanglian oral liquid+azithromycin | azithromycin | Shuanghuanglian oral liquid (1-3age:10ml,po,tid; 4-7age:20ml,po,tid)+ azithromycin (sequential therapy:15mg/(kg·d),ivgtt,qd and 10-12mg/(kg·d),po,qd) | azithromycin (sequential therapy:15mg/(kg·d),ivgtt,qd and 10-12mg/(kg·d),po,qd) | ①②③④⑩ |
| Zhang 2017 | 35 | 35 | 19 | 17 | 16 | 18 | 4.61±1.23 | 4.01±1.42 | 2.69±1.09 | 2.97±1.46 | - | Shuanghuanglian oral liquid+azithromycin | azithromycin | Shuanghuanglian oral liquid (1-3age:10ml,po,tid; 4-7age:20ml,po,tid)+ azithromycin (sequential therapy:10mg/(kg·d),ivgtt,qd and 10mg/(kg·d),po,qd) | azithromycin (sequential therapy:10mg/(kg·d),ivgtt,qd and 10mg/(kg·d),po,qd) | ①②③④⑤⑥⑧ |
| Gao 2019 | 52 | 52 | 29 | 31 | 23 | 21 | 5.35±0.7 | 5.49±0.62 | 4.18±0.56 | 4.22±0.57 | 11 | Pudilan Xiaoyan oral liquid+azithromycin | azithromycin | Pudilan Xiaoyan oral liquid (10ml,po,tid)+ azithromycin (sequential therapy:10mg/(kg·d),ivgtt,qd and 0.5g/d),po,qd) | azithromycin (sequential therapy:10mg/(kg·d),ivgtt,qd and 0.5g/d),po,qd) | ①②③④⑩ |
| Guo 2016a | 50 | 50 | 26 | 27 | 24 | 23 | 4.5±1.7 | 4.3±1.5 | 2.8±0.4 | 3.5±0.6 | 5 | Pudilan Xiaoyan oral liquid+azithromycin | azithromycin | Pudilan Xiaoyan oral liquid (10ml,po,tid)+ azithromycin (10mg/(kg·d),ivgtt,qd) | azithromycin (10mg/(kg·d),ivgtt,qd) | ①⑩ |
| Guo 2016b | 50 | 50 | - | - | - | - | 5.3±1.1 | 5.3±1.1 | 3.4±0.7 | 3.4±0.7 | 14 | Pudilan Xiaoyan oral liquid+azithromycin | azithromycin | Pudilan Xiaoyan oral liquid (1-3age:10ml,po,bid; 4-7age:15ml,po,bid;6-10age: 20ml,po,bid;10-13age:30ml,po,tid)+ azithromycin (sequential therapy:10-15mg/(kg·d),ivgtt,qd and 10-12mg/(kg·d),po,qd) | azithromycin (sequential therapy:10-15mg/(kg·d),ivgtt,qd and 10-12mg/(kg·d),po,qd) | ①②③④⑦⑧⑩ |
| Wang 2009 | 90 | 90 | 48 | 49 | 42 | 41 | 5±4 | 5±4 | 3.1±2 | 3.1±2 | 21 | Pudilan Xiaoyan oral liquid+azithromycin | azithromycin | Pudilan Xiaoyan oral liquid (5-10ml,po,bid)+ azithromycin (sequential therapy:10mg/(kg·d),ivgtt,qd and 10mg/(kg·d),po,qd) | azithromycin (sequential therapy:10mg/(kg·d),ivgtt,qd and 10mg/(kg·d),po,qd) | ① |
| Xia 2015 | 54 | 54 | 61 |  | 47 |  | 5.1±1.2 | 3.5±0.9 | - | - | 14 | Pudilan Xiaoyan oral liquid+azithromycin | azithromycin | Pudilan Xiaoyan oral liquid (1-3age:3ml,po,bid; 3-6age:5ml,po,tid;6-10age: 10ml,po,bid;10-13age:30ml,po,tid)+ azithromycin (sequential therapy:15mg/(kg·d),ivgtt,qd and 12mg/(kg·d),po,qd) | azithromycin (sequential therapy:15mg/(kg·d),ivgtt,qd and 12mg/(kg·d),po,qd) | ①②③④⑦⑧ |
| Zhang 2015 | 44 | 36 | 25 | 20 | 19 | 16 | 5.46±0.49 | 5.3±0.75 | 4.62±0.46 | 4.24±0.55 | 11 | Pudilan Xiaoyan oral liquid+azithromycin | azithromycin | Pudilan Xiaoyan oral liquid (10ml,po,bid)联合azithromycin (sequential therapy:10mg/(kg·d),ivgtt,qd) | azithromycin (10mg/(kg·d),ivgtt,qd) | ①②③④ |
| Bai 2017 | 40 | 40 | 20 | 21 | 20 | 19 | 7.5±2.3 | 7.6±2.1 | - | - | - | Xiaoer Feire Kechuan oral liquid+azithromycin | azithromycin | Xiaoer Feire Kechuan oral liquid (1-3age:10ml,po,tid; 4-7age:10ml,po,qid;7-14age: 20ml,po,tid)+ azithromycin (5-10mg/(kg·d),qd) | azithromycin (5-10mg/(kg·d),qd) | ① |
| Cai 2018 | 50 | 50 | 27 | 28 | 23 | 22 | 5.12±2.33 | 5.1±2.32 | 3.6±1.32 | 3.62±1.33 | 14 | Xiaoer Feire Kechuan oral liquid+azithromycin | azithromycin | Xiaoer Feire Kechuan oral liquid (＜7age:10ml,po,qid;≥7age: 20ml,po,tid)+ azithromycin (sequential therapy:10mg/(kg·d),ivgtt,qd and 10mg/(kg·d),po,qd) | azithromycin (sequential therapy:10mg/(kg·d),ivgtt,qd and 10mg/(kg·d),po,qd) | ①②③ |
| Chen 2011 | 81 | 81 | 79 |  | 83 |  | 4.6±1.4 | 4.6±1.4 | 13.6±3.8 | 13.6±3.8 | 21 | Xiaoer Feire Kechuan oral liquid+azithromycin | azithromycin | Xiaoer Feire Kechuan oral liquid (10ml,po,tid)+ azithromycin (sequential therapy:10-15mg/(kg·d),ivgtt,qd and 10-15mg/(kg·d),po,qd) | azithromycin (sequential therapy:10-15mg/(kg·d),ivgtt,qd and 10-15mg/(kg·d),po,qd) | ① |
| Ding 2019 | 42 | 42 | 23 | 24 | 19 | 18 | 8.49±1.05 | 7.15±1.49 | - | - | - | Xiaoer Feire Kechuan oral liquid+azithromycin | azithromycin | Xiaoer Feire Kechuan oral liquid (1-3age:10ml,po,qd; 4-7age:40ml,po,qd;≥7age: 60ml,po,qd)+ azithromycin (5mg/(kg·d),po,qd) | azithromycin (5mg/(kg·d),ivgtt,qd) | ②③ |
| Dong 2020 | 24 | 24 | 12 | 11 | 12 | 13 | 7.82±2.13 | 8.44±2.84 | - | - | 7 | Xiaoer Feire Kechuan oral liquid+azithromycin | azithromycin | Xiaoer Feire Kechuan oral liquid (10ml,po,tid)+ azithromycin (10mg/(kg·d),maximum dose=0.5g,ivgtt,qd) | azithromycin (10mg/(kg·d),maximum dose=0.5g,ivgtt,qd) | ①②③④⑩ |
| Fang 2019 | 47 | 47 | 26 | 25 | 21 | 22 | 6.11±1.27 | 6.34±1.08 | - | - | 11 | Xiaoer Feire Kechuan oral liquid+azithromycin | azithromycin | Xiaoer Feire Kechuan oral liquid (1-3age:10ml,po,tid; 4-7age:10ml,po,qid;8-14age: 20ml,po,tid)+ azithromycin (sequential therapy:10mg/(kg·d),ivgtt,qd and 10mg/(kg·d),po,qd) | azithromycin (sequential therapy:10mg/(kg·d),ivgtt,qd and 10mg/(kg·d),po,qd) | ①②③ |
| Gao 2018 | 40 | 40 | 25 | 10 | 15 | 30 | 6.4±2.3 | 8.1±4.3 | - | - | 11 | Xiaoer Feire Kechuan oral liquid+azithromycin | azithromycin | Xiaoer Feire Kechuan oral liquid (1-3age:10ml,po,tid; 4-7age:10ml,po,qid;8-14age: 20ml,po,tid)+ azithromycin (sequential therapy:0.1g/kg,ivgtt,qd and NAmg/(kg·d),po) | azithromycin (sequential therapy:0.1g/kg,ivgtt,qd and NA mg/(kg·d),po) | ①③ |
| Han 2016 | 34 | 34 | 19 | 18 | 15 | 16 | 3.58±0.56 | 3.14±0.22 | 10.05±1.77 | 11.02±1.05 | 14 | Xiaoer Feire Kechuan oral liquid+azithromycin | azithromycin | Xiaoer Feire Kechuan oral liquid (0-1age:10ml,po,tid; 1-3age:10ml,po,qid;＞3age: 20ml,po,tid)+ azithromycin (sequential therapy:10mg/(kg·d),ivgtt,qd and 10mg/(kg·d),po,qd) | azithromycin (sequential therapy:10mg/(kg·d),ivgtt,qd and 10mg/(kg·d),po,qd) | ①③④⑩ |
| Jin 2016 | 40 | 40 | 19 | 21 | 21 | 19 | 7.1±2 | 7.2±2.1 | 10.5±1.5 | 10.2±1.6 | 14 | Xiaoer Feire Kechuan oral liquid+azithromycin | azithromycin | Xiaoer Feire Kechuan oral liquid (≤7age:10ml,po,qid;≥8age: 20ml,po,tid)+ azithromycin (sequential therapy:10mg/(kg·d),ivgtt,qd and 10mg),po,qd) | azithromycin (sequential therapy:10mg/(kg·d),ivgtt,qd and 10mg),po,qd) | ①②③⑩ |
| Li 2019 | 100 | 100 | 51 | 53 | 49 | 47 | 4.98±1.15 | 5.21±1.03 | 4.2±1.3 | 4.16±1.28 | 21 | Xiaoer Feire Kechuan oral liquid+azithromycin | azithromycin | Xiaoer Feire Kechuan oral liquid (1-3age:10ml,po,tid; 4-7age:10ml,po,qid;8-14age: 20ml,po,tid)+ azithromycin (sequential therapy:10mg/(kg·d),ivgtt,qd and 10mg),po,qd) | azithromycin (sequential therapy:10mg/(kg·d),ivgtt,qd and 10mg/(kg·d),po,qd) | ②③⑨ |
| Lin 2019 | 88 | 87 | 50 | 48 | 38 | 39 | 7.23±1.73 | 7.12±1.61 | 9.11±1.52 | 9.06±1.46 | 42 | Xiaoer Feire Kechuan oral liquid+azithromycin | azithromycin | Xiaoer Feire Kechuan oral liquid (5-7age:10ml,po,qid;8-13age: 20ml,po,tid)+ azithromycin (sequential therapy:10mg/(kg·d),ivgtt,qd and 0.1g),po,qd) | azithromycin (sequential therapy:10mg/(kg·d),ivgtt,qd and 0.1g),po,qd) | ①②③④⑨⑦⑧⑩ |
| Liu 2017a | 50 | 50 | 27 | 26 | 23 | 24 | 7.9±2.4 | 7.9±2.1 | - | - | 5 | Xiaoer Feire Kechuan oral liquid+azithromycin | azithromycin | Xiaoer Feire Kechuan oral liquid (1-3age:10ml,po,tid; 4-7age:10ml,po,qid;8-14age: 20ml,po,tid)+ azithromycin (NAmg/(kg·d),ivgtt,qd) | azithromycin (NA mg/(kg·d),ivgtt,qd) | ① |
| Liu 2017b | 38 | 38 | 22 | 20 | 16 | 18 | 6.4±2.3 | 6.9±2.5 | - | - | 12 | Xiaoer Feire Kechuan oral liquid+azithromycin | azithromycin | Xiaoer Feire Kechuan oral liquid (4-7age:10ml,po,qid;8-12age: 20ml,po,tid)+ azithromycin (sequential therapy:10mg/(kg·d),ivgtt,qd and 10mg/(kg·d),po,qd) | azithromycin (sequential therapy:10mg/(kg·d),ivgtt,qd and 10mg/(kg·d),po,qd) | ①②③ |
| Luan 2020 | 50 | 50 | 29 | 28 | 21 | 22 | 4.01±2.25 | 4.21±2.11 | 5.71±1.24 | 5.21±1.08 | 5 | Xiaoer Feire Kechuan oral liquid+azithromycin | azithromycin | Xiaoer Feire Kechuan oral liquid ＜3age:10ml,po,tid; ≥3age:10ml,po,qid)+ azithromycin (10mg/(kg·d),ivgtt,qd) | azithromycin (10mg/(kg·d),ivgtt,qd) | ①②③④⑤⑨⑩ |
| Luo 2017 | 41 | 41 | 45 |  | 37 |  | 5.1±2.3 | 5.1±2.3 | 3.6±1.32 | 3.6±1.32 | 7 | Xiaoer Feire Kechuan oral liquid+azithromycin | azithromycin | Xiaoer Feire Kechuan oral liquid (1-3age:10ml,po,tid; 3-5age:10ml,po,qid;6-12age: 15-20ml,po,tid)+ azithromycin (sequential therapy:10mg/(kg·d),ivgtt,qd and 10mg/(kg·d),po,qd) | azithromycin (sequential therapy:10mg/(kg·d),ivgtt,qd and 10mg/(kg·d),po,qd) | ①③④⑤⑨⑦⑧ |
| Ma 2018 | 60 | 60 | 36 | 34 | 24 | 26 | 6.79±1.93 | 6.71±1.78 | 4.28±1.37 | 4.13±1.42 | 7 | Xiaoer Feire Kechuan oral liquid+azithromycin | azithromycin | Xiaoer Feire Kechuan oral liquid (1-3age:10ml,po,tid; 4-7age:10ml,po,qid;8-12age: 20ml,po,tid)+ azithromycin (5-10mg/(kg·d),ivgtt,qd) | azithromycin (5-10mg/(kg·d),ivgtt,qd) | ①②④⑤⑨⑦⑧ |
| Mei 2016 | 45 | 45 | 25 | 23 | 20 | 22 | 6.5±2.6 | 5.8±2.3 | - | - | 14 | Xiaoer Feire Kechuan oral liquid+azithromycin | azithromycin | Xiaoer Feire Kechuan oral liquid (1-3age:10ml,po,tid; 4-7age:10ml,po,qid;＞8age: 20ml,po,tid)+ azithromycin (sequential therapy:10mg/(kg·d),ivgtt,qd and NAmg/(kg·d),po,qd) | azithromycin (sequential therapy:10mg/(kg·d),ivgtt,qd and NA mg/(kg·d),po,qd) | ①②③⑤⑥ |
| Meng 2016 | 38 | 42 | 18 | 20 | 20 | 22 | 5.19±2.21 | 5.78±3.32 | - | - | - | Xiaoer Feire Kechuan oral liquid+azithromycin | azithromycin | Xiaoer Feire Kechuan oral liquid (1-3age:10ml,po,tid; 4-7age:10ml,po,qid;＞8-14age: 20ml,po,tid)+ azithromycin (5-10mg/(kg·d),po,qd) | azithromycin (5-10mg/(kg·d),po,qd) | ②③④ |
| Pang 2007 | 130 | 128 | 135 |  | 123 |  | - | - | - | - | 7 | Xiaoer Feire Kechuan oral liquid+azithromycin | azithromycin | Xiaoer Feire Kechuan oral liquid (1-3age:10ml,po,tid; 4-7age:10ml,po,qid;＞8-14age: 20ml,po,tid)+ azithromycin (NAmg/(kg·d),ivgtt,qd) | azithromycin (NA mg/(kg·d),ivgtt,qd) | ① |
| Song 2019 | 30 | 30 | 19 | 17 | 11 | 13 | 6.5±6.5 | 7.5±6.5 | 8.5±3.5 | 9.5±3.5 | - | Xiaoer Feire Kechuan oral liquid+azithromycin | azithromycin | Xiaoer Feire Kechuan oral liquid (1-3age:10ml,po,tid; 4-7age:10ml,po,qid;＞8-14age: 20ml,po,tid)+ azithromycin (5-10mg/(kg·d),maximum dose=0.5g,po,qd) | azithromycin (5-10mg/(kg·d),maximum dose=0.5g,po,qd) | ②③④ |
| Tan 2019 | 39 | 39 | 19 | 21 | 20 | 18 | 7.5±2 | 7.6±1.9 | - | - | 14 | Xiaoer Feire Kechuan oral liquid+azithromycin | azithromycin | Xiaoer Feire Kechuan oral liquid (≤7age:10ml,po,qid;＞8-14age: 20ml,po,tid)+ azithromycin (sequential therapy:10mg/(kg·d),ivgtt,qd and 10mg/(kg·d),po,qd) | azithromycin (sequential therapy:10mg/(kg·d),ivgtt,qd and 10mg/(kg·d),po,qd) | ①②③⑩ |
| Wang 2019a | 60 | 60 | 28 | 31 | 32 | 29 | 6.35±0.42 | 6.02±0.35 | - | - | 14 | Xiaoer Feire Kechuan oral liquid+azithromycin | azithromycin | Xiaoer Feire Kechuan oral liquid (20ml,po,tid)+ azithromycin (10mg/(kg·d),ivgtt,qd) | azithromycin (10mg/(kg·d),ivgtt,qd) | ①⑩ |
| Wang 2019b | 24 | 24 | 10 | 14 | 14 | 10 | 3.4 | 3.3 | - | - | 14 | Xiaoer Feire Kechuan oral liquid+azithromycin | azithromycin | Xiaoer Feire Kechuan oral liquid (1-3age:10ml,po,qid;＞3age: 20ml,po,tid or pid)+ azithromycin (10mg/(kg·d),ivgtt,qd) | azithromycin (10mg/(kg·d),ivgtt,qd) | ① |
| Xiong 2018 | 47 | 47 | 25 | 24 | 22 | 23 | 7.44±2.16 | 7.32±2.21 | 9.89±2.16 | 3.93±2.22 | 14 | Xiaoer Feire Kechuan oral liquid+azithromycin | azithromycin | Xiaoer Feire Kechuan oral liquid (≤7age:10ml,po,qid;＞7age: 20ml,po,tid)+ azithromycin (sequential therapy:10mg/(kg·d),ivgtt,qd and 10mg/(kg·d),po,qd) | azithromycin (sequential therapy:10mg/(kg·d),ivgtt,qd and 10mg/(kg·d),po,qd) | ①②③④⑤⑨⑦⑧ |
| Xu 2016 | 25 | 25 | 11 | 12 | 14 | 13 | 7.21±1.65 | 6.78±1.52 | - | - | 14 | Xiaoer Feire Kechuan oral liquid+azithromycin | azithromycin | Xiaoer Feire Kechuan oral liquid (1-3age:10ml,po,tid; 4-7age:10ml,po,qid;＞8-14age:20ml,po,tid)+ azithromycin (sequential therapy:10mg/(kg·d),ivgtt,qd and 10mg/(kg·d),po,qd) | azithromycin (sequential therapy:10mg/(kg·d),ivgtt,qd and 10mg/(kg·d),po,qd) | ①②③④⑥ |
| Yang 2018 | 65 | 65 | 35 | 36 | 30 | 29 | 4.8±0.4 | 4.5±0.5 | 5.6±0.3 | 5.5±0.4 | 14 | Xiaoer Feire Kechuan oral liquid+azithromycin | azithromycin | Xiaoer Feire Kechuan oral liquid (20ml,po,tid or qid)+ azithromycin (10mg/(kg·d),ivgtt,qd) | azithromycin (10mg/(kg·d),ivgtt,qd) | ① |
| Yang 2020 | 59 | 59 | 30 | 29 | 29 | 30 | 8.45±1.73 | 8.39±1.64 | - | - | 14 | Xiaoer Feire Kechuan oral liquid+azithromycin | azithromycin | Xiaoer Feire Kechuan oral liquid (1-3age:10ml,po,tid;＞4age:20ml,po,tid)+ azithromycin (5-10mg/(kg·d),ivgtt,qd) | azithromycin (5-10mg/(kg·d),ivgtt,qd) | ① |
| Yu 2016 | 43 | 43 | 24 | 22 | 19 | 21 | 7.2 | 8.5 | 3.5 | 4.6 | 14 | Xiaoer Feire Kechuan oral liquid+azithromycin | azithromycin | Xiaoer Feire Kechuan oral liquid +azithromycin (sequential therapy:10mg/(kg·d),ivgtt,qd and 10mg/(kg·d),po,qd) | azithromycin (sequential therapy:10mg/(kg·d),ivgtt,qd and 10mg/(kg·d),po,qd) | ①②③ |
| Zhu 2019 | 35 | 35 | 19 | 18 | 16 | 17 | 5.1±2.5 | 5.4±2.1 | 5.8±2.3 | 5.6±2.6 | 7 | Xiaoer Feire Kechuan oral liquid+azithromycin | azithromycin | Xiaoer Feire Kechuan oral liquid (1-3age:10ml,po,tid; 4-7age:10ml,po,qid;＞8-14age:20ml,po,tid)+ azithromycin (10mg/(kg·d),po,qd) | azithromycin (10mg/(kg·d),po,qd) | ①②③④⑨⑧⑩ |
| Zhu 2017 | 33 | 33 | 22 | 18 | 11 | 15 | 7.2±0.6 | 7.2±0.3 | - | - | - | Xiaoer Feire Kechuan oral liquid+azithromycin | azithromycin | Xiaoer Feire Kechuan oral liquid (1-3age:10ml,po,tid; 4-7age:10ml,po,qid;＞8-12age:20ml,po,tid)+ azithromycin (sequential therapy:0.1g/ml,ivgtt,qd and NAmg/(kg·d),po,qd) | azithromycin (sequential therapy:0.1g/ml,ivgtt,qd and NA mg/(kg·d),po,qd) | ① |
| Chao 2019 | 52 | 52 | 30 | 32 | 22 | 20 | 5.27±1.32 | 5.13±1.26 | 8.12±2.46 | 7.94±2.34 | 14 | Xiaoer Xiaoji Zhike oral liquid+azithromycin | azithromycin | Xiaoer Xiaoji Zhike oral liquid (＜5age:10ml,po,tid; ≥5age:15ml,tid)+ azithromycin (10mg/(kg·d),ivgtt,qd) | azithromycin (10mg/(kg·d),ivgtt,qd) | ①⑦ |
| Chen 2018 | 66 | 66 | 35 | 34 | 31 | 32 | 6.4±2.5 | 6.5±2.4 | - | - | 10 | Xiaoer Xiaoji Zhike oral liquid+azithromycin | azithromycin | Xiaoer Xiaoji Zhike oral liquid (＜5age:10ml,po,tid; ≥5age:15ml,tid)+ azithromycin (1day:10mg/kg; 2-10days:5mg/kg,maximum dose＜0.25g,ivgtt,qd) | azithromycin (1day:10mg/kg; 2-10days:5mg/kg,maximum dose＜0.25g,po,qd) | ①②③④⑩ |
| Hu 2018 | 86 | 86 | 42 | 41 | 44 | 45 | 8.25±3.71 | 8.31±2.55 | 18.33±4.12 | 18.43±4.36 | 28 | Xiaoer Xiaoji Zhike oral liquid+azithromycin | azithromycin | Xiaoer Xiaoji Zhike oral liquid (＜5age:10ml,po,tid; ≥5age:15ml,po,tid)+ azithromycin (10mg/kg,maximum dose＜0.5g,po,qd) | azithromycin (10mg/kg,maximum dose＜0.5g,po,qd) | ①②③④⑤⑩ |
| Huang 2019 | 108 | 108 | 54 | 58 | 54 | 50 | 3.1±0.5 | 3.4±0.3 | 6.1±1.3 | 6.31±1.7 | 30 | Xiaoer Xiaoji Zhike oral liquid+azithromycin | azithromycin | Xiaoer Xiaoji Zhike oral liquid (15ml,po,tid)+ azithromycin (sequential therapy:10mg/(kg·d),ivgtt,qd and 10mg/(kg·d),po,qd) | azithromycin (sequential therapy:10mg/(kg·d),ivgtt,qd and 10mg/(kg·d),po,qd) | ① |
| Li 2018a | 42 | 42 | 20 | 17 | 22 | 25 | 5.5±2.1 | 5.2±1.5 | - | - | 14 | Xiaoer Xiaoji Zhike oral liquid+azithromycin | azithromycin | Xiaoer Xiaoji Zhike oral liquid (1-4age:10ml,po,tid; ≥5age:20ml,po,tid)+ azithromycin (10mg/(kg·d),ivgtt,qd) | azithromycin (10mg/(kg·d),ivgtt,qd) | ①②③④ |
| Li 2018b | 63 | 63 | 36 | 34 | 27 | 29 | 5.6±1.3 | 5.5±1.2 | 3.8±0.7 | 3.8±0.6 | 7 | Xiaoer Xiaoji Zhike oral liquid+azithromycin | azithromycin | Xiaoer Xiaoji Zhike oral liquid (10ml,po,tid)+ azithromycin (10mg/(kg·d),ivgtt,qd) | azithromycin (10mg/(kg·d),ivgtt,qd) | ①⑨ |
| Wang 2016 | 32 | 32 | 14 | 17 | 18 | 15 | 6.32±2.14 | 7.54±2.75 | - | - | 7 | Xiaoer Xiaoji Zhike oral liquid+azithromycin | azithromycin | Xiaoer Xiaoji Zhike oral liquid (1-2age:10mg,po,tid; 3-4age:15mg,po,tid; ＞5age:20mg,po,tid)+ azithromycin (1day:10mg/kg,maximum dose＜0.5g; 2-10days:5mg/kg,maximum dose＜0.25g,po,qd) | azithromycin (1day:10mg/kg,maximum dose＜0.5g; 2-10days:5mg/kg,maximum dose＜0.25g,po,qd) | ① |
| Wang 2019c | 54 | 54 | 27 | 29 | 27 | 25 | 3.11±0.87 | 3.05±0.91 | 6.19±1.34 | 6.2±1.25 | 30 | Xiaoer Xiaoji Zhike oral liquid+azithromycin | azithromycin | Xiaoer Xiaoji Zhike oral liquid (15ml,po,tid)+ azithromycin (sequential therapy:10mg/(kg·d),ivgtt,qd and 10mg/(kg·d),po,qd) | azithromycin (sequential therapy:10mg/(kg·d),ivgtt,qd and 10mg/(kg·d),po,qd) | ①②③④⑩ |
| Xie 2015 | 60 | 60 | 31 | 30 | 29 | 30 | 5.19±2.38 | 5.21±2.46 | - | - | 7 | Xiaoer Xiaoji Zhike oral liquid+azithromycin | azithromycin | Xiaoer Xiaoji Zhike oral liquid (＜5age:10ml,po,tid;＞5age:15ml,po,tid)+ azithromycin (1day:10mg/kg; 2-10days:5mg/kg,po,qd) | azithromycin (1day:10mg/kg; 2-10days:5mg/kg,po,qd) | ① |
| Xue 2020 | 46 | 46 | 19 | 18 | 27 | 28 | 5.41±1.1 | 5.24±1.03 | 5.53±1.3 | 5.38±1.25 | 14 | Xiaoer Xiaoji Zhike oral liquid+azithromycin | azithromycin | Xiaoer Xiaoji Zhike oral liquid (＜5age:10ml,po,tid;＞5age:15ml,po,tid)+ azithromycin (1day:10mg/kg; 2-5 days:0.5g/kg,po,qd) | azithromycin (1day:10mg/kg; 2-5days:0.5g/kg,po,qd) | ①②③④⑥⑩ |
| Yang 2019 | 22 | 22 | 12 | 12 | 10 | 10 | 6.1±1.96 | 5.73±2.38 | - | - | 14 | Xiaoer Xiaoji Zhike oral liquid+azithromycin | azithromycin | Xiaoer Xiaoji Zhike oral liquid (＜5age:10ml,po,tid;＞5age:15ml,po,tid)+ azithromycin (10mg/(kg·d),ivgtt,qd) | azithromycin (10mg/(kg·d),ivgtt,qd) | ① |
| Ye 2018 | 40 | 40 | 23 | 15 | 17 | 25 | 6.9±1.2 | 7.3±1.5 | - | - | 7 | Xiaoer Xiaoji Zhike oral liquid+azithromycin | azithromycin | Xiaoer Xiaoji Zhike oral liquid (＜1age:5ml,po,tid; 1-2age:10ml,po,tid; 3-4age:15ml,po,tid;＞5age:20ml,po,tid)+ azithromycin (1day:10mg/kg; 2-7days:0.5g/kg,po,qd) | azithromycin (1day:10mg/kg; 2-7days:0.5g/kg,po,qd) | ① |
| Yu 2017 | 31 | 31 | 16 | 14 | 15 | 17 | 6.32±3.12 | 6.94±2.76 | - | - | 7 | Xiaoer Xiaoji Zhike oral liquid+azithromycin | azithromycin | Xiaoer Xiaoji Zhike oral liquid (1-2age:10ml,po,tid; 3-4age:15ml,po,tid;＞5age:20ml,po,tid)+ azithromycin (1day:10mg/kg; 2-7days:0.5g/kg,po,qd) | azithromycin (1day:10mg/kg; 2-7days:0.5g/kg,po,qd) | ① |
| Yuan 2015 | 43 | 43 | 16 | 14 | 27 | 29 | 7.56±2.28 | 7.92±3.12 | 2.31±1.42 | 2.23±1.51 | 14 | Xiaoer Xiaoji Zhike oral liquid+azithromycin | azithromycin | Xiaoer Xiaoji Zhike oral liquid (＜5age:10ml,po,tid;＞5age:15ml,po,tid)+ azithromycin (10mg/(kg·d),ivgtt,qd) | azithromycin (10mg/(kg·d),ivgtt,qd) | ①②③④⑤⑥ |
| Zhao 2019 | 35 | 35 | 19 | 20 | 16 | 15 | 7.55±2.06 | 7.51±2.02 | 6.96±1.54 | 7±1.57 | 14 | Xiaoer Xiaoji Zhike oral liquid+azithromycin | azithromycin | Xiaoer Xiaoji Zhike oral liquid (4-5age:10ml,po,tid;＞5age:15ml,po,tid)+ azithromycin (10mg/(kg·d),ivgtt,qd) | azithromycin (10mg/(kg·d),ivgtt,qd) | ①②③④⑥⑩ |
| Zheng 2018 | 30 | 30 | 18 | 13 | 12 | 17 | 8.01±2.32 | 2.47±2.06 | 5.32±1.54 | 5.61±1.13 | 30 | Xiaoer Xiaoji Zhike oral liquid+azithromycin | azithromycin | Xiaoer Xiaoji Zhike oral liquid (3-4age:15ml,po,tid;＞5age:20ml,po,tid)+ azithromycin (NAmg/(kg·d),ivgtt,qd) | azithromycin (NA mg/(kg·d),ivgtt,qd) | ①⑩ |
| Zong 2019 | 47 | 46 | 26 | 25 | 21 | 21 | 3.27±0.77 | 3.19±0.68 | 3.41±0.62 | 3.27±0.71 | 15 | Xiaoer Xiaoji Zhike oral liquid+azithromycin | azithromycin | Xiaoer Xiaoji Zhike oral liquid (10ml,po,bid)+ azithromycin (sequential therapy:10mg/(kg·d),ivgtt,qd and 5mg/(kg·d),po,qd) | azithromycin (sequential therapy:10mg/(kg·d),ivgtt,qd and 5mg/(kg·d),po,qd) | ①②③④⑥ |

**Groups:** T: Treatment group; C: Control group

**Outcome measurements:①**Response rate; ②disappearance time of cough; ③disappearance time of fever; ④disappearance time of pulmonary rales; ⑤Average hospitalization time; ⑥disappearance time of pulmonary shadows in X‐ray; ⑦serum level of TNF-α; ⑧IL‐6; ⑨C-reaction protein; ⑩Adverse events.

# Table 4 The SUCRA of Primary Outcomes in Biplot

| Interventions | [Compare](E:/Program%20Files%20(x86)/Youdao/Dict/8.9.3.0/resultui/html/index.html#/javascript:;) with Primary Outcome | disappearance time of cough | disappearance time of fever | disappearance time of pulmonary rales |
| --- | --- | --- | --- | --- |
| SHL+Azithromycin | clinical effective rate | 37.12% | 31.36% | 39.68% |
| PDLXY+Azithromycin | clinical effective rate | 36.54% | 36.54% | 28.56% |
| XEFRKC+Azithromycin | clinical effective rate | 35.99% | 17.11% | 23.01% |
| XEXJZK+Azithromycin | clinical effective rate | 37.40% | 72.25% | 68.00% |
| Azithromycin | clinical effective rate | 0% | 0% | 0% |

**Interventions:** PDLXY: Pudilan Xiaoyan oral liquid; SHL: Shuanghuanlian oral liquid; XEFRKC: Xiaoer Feire Kechuan oral liquid; XRXJZK: Xiaoer Xiaoji Zhike oral liquid.

# Table 5 League table of Length of Hospitalization Time and Disappearance Time of Pulmonary Shadows in X-ray


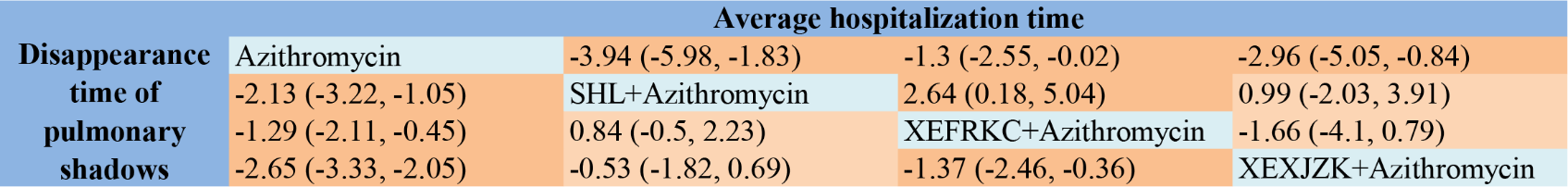


**Dark brown represents statistical significance. Light brown represents no statistical significance.**

**Interventions:** PDLXY: Pudilan Xiaoyan oral liquid; SHL: Shuanghuanlian oral liquid; XEFRKC: Xiaoer Feire Kechuan oral liquid; XRXJZK: Xiaoer Xiaoji Zhike oral liquid.

# Table 6 League Table of Interleukin-6 and Tumor Necrosis Factor-α


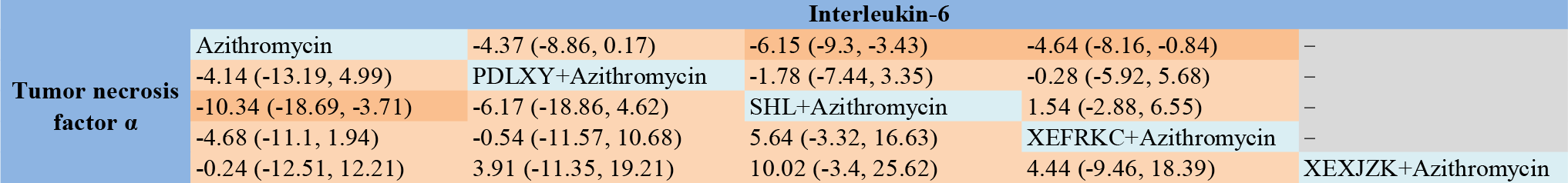


**Dark brown represents statistical significance. Light brown represents no statistical significance. Gray represents inexistence.**

**Interventions:** PDLXY: Pudilan Xiaoyan oral liquid; SHL: Shuanghuanlian oral liquid; XEFRKC: Xiaoer Feire Kechuan oral liquid; XRXJZK: Xiaoer Xiaoji Zhike oral liquid.

# Table 7 League Table of C-reactive Protein


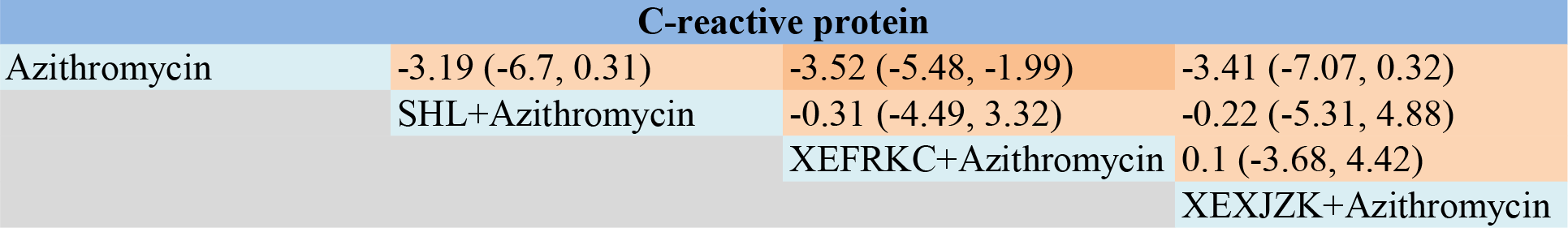


**Dark brown represents statistical significance. Light brown represents no statistical significance. Gray represents inexistence.**

**Interventions:** PDLXY: Pudilan Xiaoyan oral liquid; SHL: Shuanghuanlian oral liquid; XEFRKC: Xiaoer Feire Kechuan oral liquid; XRXJZK: Xiaoer Xiaoji Zhike oral liquid.

# Table 8 Adverse Events

| **PDLXY** | | | | | | | | | | | | | | | | | | | | | | | | | | | | | | | | | | | | | | | | | |
| --- | --- | --- | --- | --- | --- | --- | --- | --- | --- | --- | --- | --- | --- | --- | --- | --- | --- | --- | --- | --- | --- | --- | --- | --- | --- | --- | --- | --- | --- | --- | --- | --- | --- | --- | --- | --- | --- | --- | --- | --- | --- |
| **Study** | | **Treatment group** | | **Total adverse events** | | | **Nausea and vomiting** | | | | | | | | | | | | **Gastrointestinal reaction** | | | | | | | | | | | | **Pain at the injection site** | | | | | | | | | | |
| Gao 2019 | | PDLXY+Azithromycin | | 1 | | | 1 | | | | | | | | | | | |  | | | | | | | | | | | |  | | | | | | | | | | |
| Guo 2016a | | PDLXY+Azithromycin | | 1 | | | 1 | | | | | | | | | | | |  | | | | | | | | | | | |  | | | | | | | | | | |
| Guo 2016b | | PDLXY+Azithromycin | | 3 | | |  | | | | | | | | | | | | 3 | | | | | | | | | | | |  | | | | | | | | | | |
| Xia 2015 | | PDLXY+Azithromycin | | 4 | | |  | | | | | | | | | | | | 3 | | | | | | | | | | | | 1 | | | | | | | | | | |
| **SHL** | | | | | | | | | | | | | | | | | | | | | | | | | | | | | | | | | | | | | | | | | |
| **Study** | | **Treatment group** | | **Total adverse events** | | | **Nausea and vomiting** | | | | | | **Abdominal pain and diarrhea** | | | | | | | **Gastrointestinal reaction** | | | | | | | | **erythra** | | | | | | | | **Pain at the injection site** | | | | | |
| Fan 2017 | | SHL+Azithromycin | | 5 | | |  | | | | | |  | | | | | | | 2 | | | | | | | | 1 | | | | | | | | 2 | | | | | |
| Guo 2017 | | SHL+Azithromycin | | 7 | | | 3 | | | | | | 2 | | | | | | |  | | | | | | | | 2 | | | | | | | |  | | | | | |
| Liu 2016 | | SHL+Azithromycin | | 3 | | | 2 | | | | | | 1 | | | | | | |  | | | | | | | |  | | | | | | | |  | | | | | |
| Liu 2018 | | SHL+Azithromycin | | 6 | | | 2 | | | | | | 3 | | | | | | |  | | | | | | | | 1 | | | | | | | |  | | | | | |
| Wang 2018a | | SHL+Azithromycin | | 1 | | |  | | | | | | 1 | | | | | | |  | | | | | | | |  | | | | | | | |  | | | | | |
| Wang 2018b | | SHL+Azithromycin | | 3 | | | 2 | | | | | | 1 | | | | | | |  | | | | | | | |  | | | | | | | |  | | | | | |
| Yao 2018 | | SHL+Azithromycin | | 3 | | |  | | | | | | 1 | | | | | | |  | | | | | | | | 2 | | | | | | | |  | | | | | |
| Zhang 2017 | | SHL+Azithromycin | | 3 | | | 2 | | | | | |  | | | | | | |  | | | | | | | | 1 | | | | | | | |  | | | | | |
| **XEFRKC** | | | | | | | | | | | | | | | | | | | | | | | | | | | | | | | | | | | | | | | | | |
| **Study** | **Treatment group** | | | **Total adverse events** | | | **Nausea and vomiting** | | | | **Abdominal pain and diarrhea** | | | | | **Lack of appetite** | | | | | | **erythra** | | | | | **Dizziness and headache** | | | | | | **Diarrhea and abdominal pain** | | | | | **tachycardia** | | |  |
| Chen 2011 | XEFRKC+Azithromycin | | | 5 | | | 1 | | | | 4 | | | | |  | | | | | |  | | | | |  | | | | | |  | | | | |  | | |  |
| Dong 2020 | XEFRKC+Azithromycin | | | 2 | | | 1 | | | | 1 | | | | |  | | | | | |  | | | | |  | | | | | |  | | | | |  | | |  |
| Han 2016 | XEFRKC+Azithromycin | | | 4 | | | 3 | | | | 1 | | | | |  | | | | | |  | | | | |  | | | | | |  | | | | |  | | |  |
| Jin 2016 | XEFRKC+Azithromycin | | | 5 | | | 2 | | | | 2 | | | | | 1 | | | | | |  | | | | |  | | | | | |  | | | | |  | | |  |
| Lin 2019 | XEFRKC+Azithromycin | | | 16 | | |  | | | | 7 | | | | |  | | | | | | 2 | | | | |  | | | | | | 4 | | | | | 3 | | |  |
| Luan 2020 | XEFRKC+Azithromycin | | | 1 | | |  | | | |  | | | | |  | | | | | |  | | | | | 1 | | | | | |  | | | | |  | | |  |
| Mei 2016 | XEFRKC+Azithromycin | | | 5 | | |  | | | | 3 | | | | | 2 | | | | | |  | | | | |  | | | | | |  | | | | |  | | |  |
| Tan 2019 | XEFRKC+Azithromycin | | | 4 | | | 1 | | | | 1 | | | | | 2 | | | | | |  | | | | |  | | | | | |  | | | | |  | | |  |
| Wang 2019a | XEFRKC+Azithromycin | | | 2 | | | 1 | | | | 1 | | | | |  | | | | | |  | | | | |  | | | | | |  | | | | |  | | |  |
| Yu 2016 | XEFRKC+Azithromycin | | | 6 | | | 4 | | | | 2 | | | | |  | | | | | |  | | | | |  | | | | | |  | | | | |  | | |  |
| Zhu 2019 | XEFRKC+Azithromycin | | | 4 | | |  | | | | 2 | | | | | 2 | | | | | |  | | | | |  | | | | | |  | | | | |  | | |  |
| **XEXJZK** | | | | | | | | | | | | | | | | | | | | | | | | | | | | | | | | | | | | | | | | | |
| **Study** | **Treatment group** | | | **Total adverse events** | | | **Nausea and vomiting** | | **Abdominal pain and diarrhea** | | | | | **Gastrointestinal reaction** | | | **abdominal distention** | | | | | | **constipation** | | **erythra** | | | | | **Skin allergies** | | | | **Dizziness and headache** | | | | | **Throat irritation** | | |
| Chen 2018 | XEXJZK+Azithromycin | | | 29 | | | 3 | |  | | | | |  | | | 10 | | | | | | 13 | |  | | | | | 2 | | | | 1 | | | | |  | | |
| Hu 2018 | XEXJZK+Azithromycin | | | 10 | | |  | |  | | | | |  | | |  | | | | | |  | |  | | | | |  | | | |  | | | | |  | | |
| Wang 2019c | XEXJZK+Azithromycin | | | 12 | | | 7 | | 4 | | | | |  | | |  | | | | | |  | |  | | | | |  | | | |  | | | | | 1 | | |
| Xue 2020 | XEXJZK+Azithromycin | | | 10 | | |  | |  | | | | | 4 | | |  | | | | | |  | | 2 | | | | |  | | | | 4 | | | | |  | | |
| Zhao 2019 | XEXJZK+Azithromycin | | | 1 | | |  | |  | | | | | 1 | | |  | | | | | |  | |  | | | | |  | | | |  | | | | |  | | |
| Zheng 2018 | XEXJZK+Azithromycin | | | 2 | | |  | |  | | | | | 2 | | |  | | | | | |  | |  | | | | |  | | | |  | | | | |  | | |
| **Azithromycin** | | | | | | | | | | | | | | | | | | | | | | | | | | | | | | | | | | | | | | | | | |
| **Study** | **Control group** | | **Total adverse events** | | **Nausea and vomiting** | **Abdominal pain and diarrhea** | | **Lack of appetite** | | **Gastrointestinal reaction** | | **abdominal distention** | | | **constipation** | | | **erythra** | | | **Skin allergies** | | | **Dizziness and headache** | | **Pain at the injection site** | | | **Upper abdominal discomfort** | | | **Diarrhea and abdominal pain** | | | **hoarseness** | | **tachycardia** | | | **Liver and kidney damage** | |
| Fan 2017 | Azithromycin | | 7 | |  |  | |  | | 3 | |  | | |  | | | 2 | | |  | | |  | | 2 | | |  | | |  | | |  | |  | | |  | |
| Guo 2017 | Azithromycin | | 9 | | 3 | 3 | |  | |  | |  | | |  | | | 2 | | |  | | |  | |  | | |  | | |  | | |  | |  | | | 1 | |
| Liu 2016 | Azithromycin | | 5 | | 3 | 2 | |  | |  | |  | | |  | | |  | | |  | | |  | |  | | |  | | |  | | |  | |  | | |  | |
| Liu 2018 | Azithromycin | | 5 | | 2 | 1 | |  | |  | |  | | |  | | | 2 | | |  | | |  | |  | | |  | | |  | | |  | |  | | |  | |
| Wang 2018b | Azithromycin | | 2 | | 1 | 1 | |  | |  | |  | | |  | | |  | | |  | | |  | |  | | |  | | |  | | |  | |  | | |  | |
| Yao 2018 | Azithromycin | | 4 | | 4 |  | |  | |  | |  | | |  | | |  | | |  | | |  | |  | | |  | | |  | | |  | |  | | |  | |
| Zhang 2017 | Azithromycin | | 4 | | 2 |  | |  | |  | |  | | |  | | | 2 | | |  | | |  | |  | | |  | | |  | | |  | |  | | |  | |
| Guo 2016b | Azithromycin | | 5 | |  |  | |  | | 4 | |  | | |  | | | 1 | | |  | | |  | |  | | |  | | |  | | |  | |  | | |  | |
| Xia 2015 | Azithromycin | | 6 | |  |  | |  | | 4 | |  | | |  | | | 1 | | |  | | |  | | 1 | | |  | | |  | | |  | |  | | |  | |
| Chen 2011 | Azithromycin | | 35 | | 15 | 20 | |  | |  | |  | | |  | | |  | | |  | | |  | |  | | |  | | |  | | |  | |  | | |  | |
| Dong 2020 | Azithromycin | | 9 | | 3 | 4 | | 2 | |  | |  | | |  | | |  | | |  | | |  | |  | | |  | | |  | | |  | |  | | |  | |
| Han 2016 | Azithromycin | | 3 | | 2 | 1 | |  | |  | |  | | |  | | |  | | |  | | |  | |  | | |  | | |  | | |  | |  | | |  | |
| Jin 2016 | Azithromycin | | 6 | | 2 | 2 | | 2 | |  | |  | | |  | | |  | | |  | | |  | |  | | |  | | |  | | |  | |  | | |  | |
| Lin 2019 | Azithromycin | | 14 | |  | 6 | |  | |  | |  | | |  | | | 1 | | |  | | |  | |  | | |  | | | 3 | | |  | | 4 | | |  | |
| Luan 2020 | Azithromycin | | 1 | |  |  | |  | |  | |  | | |  | | |  | | |  | | | 1 | |  | | |  | | |  | | |  | |  | | |  | |
| Mei 2016 | Azithromycin | | 4 | |  | 2 | | 2 | |  | |  | | |  | | |  | | |  | | |  | |  | | |  | | |  | | |  | |  | | |  | |
| Tan 2019 | Azithromycin | | 7 | | 2 | 2 | | 3 | |  | |  | | |  | | |  | | |  | | |  | |  | | |  | | |  | | |  | |  | | |  | |
| Wang 2019a | Azithromycin | | 13 | | 5 | 5 | | 3 | |  | |  | | |  | | |  | | |  | | |  | |  | | |  | | |  | | |  | |  | | |  | |
| Yu 2016 | Azithromycin | | 5 | |  | 3 | |  | |  | |  | | |  | | |  | | |  | | |  | |  | | | 2 | | |  | | |  | |  | | |  | |
| Zhu 2019 | Azithromycin | | 3 | |  | 1 | | 2 | |  | |  | | |  | | |  | | |  | | |  | |  | | |  | | |  | | |  | |  | | |  | |
| Chen 2018 | Azithromycin | | 30 | | 2 |  | |  | |  | | 11 | | | 14 | | |  | | | 1 | | | 2 | |  | | |  | | |  | | |  | |  | | |  | |
| Hu 2018 | Azithromycin | | 25 | |  |  | |  | |  | |  | | |  | | |  | | |  | | |  | |  | | |  | | |  | | |  | |  | | |  | |
| Wang 2019c | Azithromycin | | 9 | | 5 | 3 | |  | |  | |  | | |  | | |  | | |  | | |  | |  | | |  | | |  | | | 1 | |  | | |  | |
| Xue 2020 | Azithromycin | | 8 | |  |  | |  | | 3 | |  | | |  | | | 2 | | |  | | | 3 | |  | | |  | | |  | | |  | |  | | |  | |
| Zhao 2019 | Azithromycin | | 2 | |  |  | |  | | 1 | |  | | |  | | |  | | |  | | | 1 | |  | | |  | | |  | | |  | |  | | |  | |
| Zheng 2018 | Azithromycin | | 3 | |  |  | |  | |  | |  | | |  | | |  | | |  | | |  | | 1 | | | 2 | | |  | | |  | |  | | |  | |

**Interventions:** PDLXY: Pudilan Xiaoyan oral liquid; SHL: Shuanghuanlian oral liquid; XEFRKC: Xiaoer Feire Kechuan oral liquid; XRXJZK: Xiaoer Xiaoji Zhike oral liquid.

# Table 9 The Meta-regression for Primary Outcomes

| Centred covariates at the following overall mean values | | | |
| --- | --- | --- | --- |
| Response rate | | | |
|  | Mean | 2.5% | 97.5% |
| β[Man percent] | 0.19 | -0.06 | 0.63 |
| β[Age] | -0.15 | -0.55 | 0.35 |
| β[drug delivery way] | 0.27 | -16.46 | 16.21 |
| β[course of treatment] | -0.16 | -0.97 | 0.39 |
| β[course of disease] | -0.05 | -0.27 | 0.17 |
| β[(PDLXY+Azithromycin)×Man percent] | 0.33 | -0.14 | 1.09 |
| β[(SHL+Azithromycin)×Man percent] | 0 | -0.24 | 0.35 |
| β[(XEFRKC+Azithromycin)×Man percent] | -0.35 | -1.33 | 0.03 |
| β[(XEXJZK+Azithromycin)×Man percent] | -0.37 | -1.46 | 0.05 |
| β[(PDLXY+Azithromycin)×Age] | -0.31 | -8.71 | 6.86 |
| β[(SHL+Azithromycin)×Age] | 0.64 | -0.41 | 1.58 |
| β[(XEFRKC+Azithromycin)×Age] | 0.25 | -0.43 | 1.48 |
| β[(XEXJZK+Azithromycin)×Age] | -0.42 | -1.73 | 0.54 |
| β[(PDLXY+Azithromycin)×drug delivery way] | -0.01 | -1.32 | 1.24 |
| β[(SHL+Azithromycin)×drug delivery way] | 0.36 | -1.08 | 2.41 |
| β[(XEFRKC+Azithromycin)×drug delivery way] | -0.04 | -0.62 | 0.63 |
| β[(XEXJZK+Azithromycin)×drug delivery way] | 0.19 | -1.08 | 1.56 |
| β[(PDLXY+Azithromycin)×course of treatment] | -0.2 | -0.59 | 0.32 |
| β[(SHL+Azithromycin)×course of treatment] | 0.54 | -0.11 | 1.89 |
| β[(XEFRKC+Azithromycin)×course of treatment] | -0.31 | -1.27 | 0.07 |
| β[(XEXJZK+Azithromycin)×course of treatment] | 0.08 | -0.08 | 0.25 |
| β[(PDLXY+Azithromycin)×course of disease] | 0.87 | -2.34 | 4.02 |
| β[(SHL+Azithromycin)×course of disease] | -0.57 | -1.83 | 0.54 |
| β[(XEFRKC+Azithromycin)×course of disease] | 0.03 | -0.13 | 0.21 |
| β[(XEXJZK+Azithromycin)×course of disease] | 0.12 | -0.19 | 0.46 |
| Disappearance time of cough | | | |
|  | Mean | 2.5% | 97.5% |
| β[Man percent] | -0.02 | -0.26 | 0.2 |
| β[Age] | 0.41 | -0.4 | 1.21 |
| β[drug delivery way] | -0.58 | -17.84 | 15.41 |
| β[course of treatment] | -0.02 | -0.13 | 0.1 |
| β[course of disease] | 0.16 | -0.15 | 0.45 |
| β[(PDLXY+Azithromycin)×Man percent] | 0.01 | -8.48 | 8.43 |
| β[(SHL+Azithromycin)×Man percent] | -0.12 | -0.33 | 0.09 |
| β[(XEFRKC+Azithromycin)×Man percent] | -0.05 | -0.11 | 0.02 |
| β[(XEXJZK+Azithromycin)×Man percent] | -0.04 | -0.19 | 0.11 |
| β[(PDLXY+Azithromycin)×Age] | 1.91 | -10.48 | 14.26 |
| β[(SHL+Azithromycin)×Age] | 0.39 | -2.1 | 2.87 |
| β[(XEFRKC+Azithromycin)×Age] | 0.15 | -0.3 | 0.61 |
| β[(XEXJZK+Azithromycin)×Age] | 0.71 | -0.28 | 1.67 |
| β[(PDLXY+Azithromycin)×drug delivery way] | -0.38 | -15.49 | 14.53 |
| β[(SHL+Azithromycin)×drug delivery way] | -0.07 | -17.52 | 17.33 |
| β[(XEFRKC+Azithromycin)×drug delivery way] | 0.7 | -0.1 | 1.49 |
| β[(XEXJZK+Azithromycin)×drug delivery way] | -1.36 | -3.6 | 0.98 |
| β[(PDLXY+Azithromycin)×course of treatment] | 0.01 | -3.37 | 3.36 |
| β[(SHL+Azithromycin)×course of treatment] | -0.06 | -0.29 | 0.17 |
| β[(XEFRKC+Azithromycin)×course of treatment] | 0.08 | -0.03 | 0.19 |
| β[(XEXJZK+Azithromycin)×course of treatment] | 0 | -0.17 | 0.17 |
| β[(PDLXY+Azithromycin)×course of disease] | 0.79 | -12.39 | 13.89 |
| β[(SHL+Azithromycin)×course of disease] | 0.96 | -2.99 | 4.94 |
| β[(XEFRKC+Azithromycin)×course of disease] | -0.2 | -0.44 | 0.06 |
| β[(XEXJZK+Azithromycin)×course of disease] | -0.08 | -0.42 | 0.26 |
| Disappearance time of fever | | | |
|  | Mean | 2.5% | 97.5% |
| β[Man percent] | 0 | -0.08 | 0.07 |
| β[Age] | -0.04 | -0.38 | 0.31 |
| β[drug delivery way] | -2.68 | -12.34 | 8.3 |
| β[course of treatment] | -0.09 | -0.23 | 0.05 |
| β[course of disease] | 0.09 | -0.03 | 0.2 |
| β[(PDLXY+Azithromycin)×Man percent] | 0.53 | -3.78 | 4.6 |
| β[(SHL+Azithromycin)×Man percent] | -0.02 | -0.08 | 0.04 |
| β[(XEFRKC+Azithromycin)×Man percent] | -0.03 | -0.07 | 0.02 |
| β[(XEXJZK+Azithromycin)×Man percent] | -0.08 | -0.21 | 0.03 |
| β[(PDLXY+Azithromycin)×Age] | 0.4 | -19.09 | 19.83 |
| β[(SHL+Azithromycin)×Age] | -0.43 | -1.03 | 0.18 |
| β[(XEFRKC+Azithromycin)×Age] | -0.1 | -0.33 | 0.16 |
| β[(XEXJZK+Azithromycin)×Age] | -0.41 | -1.03 | 0.18 |
| β[(PDLXY+Azithromycin)×drug delivery way] | 0.82 | -5.87 | 7.23 |
| β[(SHL+Azithromycin)×drug delivery way] | 1.13 | -15.26 | 18.88 |
| β[(XEFRKC+Azithromycin)×drug delivery way] | 0.14 | -0.18 | 0.49 |
| β[(XEXJZK+Azithromycin)×drug delivery way] | 0.44 | -0.82 | 1.71 |
| β[(PDLXY+Azithromycin)×course of treatment] | 0.8 | -4.44 | 5.74 |
| β[(SHL+Azithromycin)×course of treatment] | 0 | -0.23 | 0.23 |
| β[(XEFRKC+Azithromycin)×course of treatment] | 0.05 | -0.02 | 0.11 |
| β[(XEXJZK+Azithromycin)×course of treatment] | -0.05 | -0.12 | 0.03 |
| β[(PDLXY+Azithromycin)×course of disease] | 0.58 | -9.17 | 10.02 |
| β[(SHL+Azithromycin)×course of disease] | -0.09 | -0.86 | 0.68 |
| β[(XEFRKC+Azithromycin)×course of disease] | -0.16 | -0.25 | 0.05 |
| β[(XEXJZK+Azithromycin)×course of disease] | 0.21 | -0.06 | 0.51 |
| Disappearance time of pulmonary rales | | | |
|  | Mean | 2.5% | 97.5% |
| β[Man percent] | -0.01 | -0.5 | 0.42 |
| β[Age] | -0.31 | -2.19 | 1.33 |
| β[drug delivery way] | -0.13 | -18.04 | 17.12 |
| β[course of treatment] | 0.12 | -0.19 | 0.45 |
| β[course of disease] | 0.05 | -0.2 | 0.28 |
| β[(PDLXY+Azithromycin)×Man percent] | -1.83 | -4.19 | 0.67 |
| β[(SHL+Azithromycin)×Man percent] | -0.09 | -0.22 | 0.03 |
| β[(XEFRKC+Azithromycin)×Man percent] | 0.04 | -0.11 | 0.21 |
| β[(XEXJZK+Azithromycin)×Man percent] | 0.07 | -0.08 | 0.22 |
| β[(PDLXY+Azithromycin)×Age] | -1.13 | -20.32 | 18.31 |
| β[(SHL+Azithromycin)×Age] | -0.57 | -2.83 | 1.49 |
| β[(XEFRKC+Azithromycin)×Age] | 0.09 | -0.31 | 0.45 |
| β[(XEXJZK+Azithromycin)×Age] | -0.09 | -1.39 | 1.22 |
| β[(PDLXY+Azithromycin)×drug delivery way] | -2.25 | -7.92 | 3.75 |
| β[(SHL+Azithromycin)×drug delivery way] | 1.3 | -15.28 | 17.96 |
| β[(XEFRKC+Azithromycin)×drug delivery way] | -0.24 | -1.79 | 1.25 |
| β[(XEXJZK+Azithromycin)×drug delivery way] | 0.57 | -2.74 | 3.74 |
| β[(PDLXY+Azithromycin)×course of treatment] | 1.22 | -1.81 | 4.3 |
| β[(SHL+Azithromycin)×course of treatment] | 0.03 | -0.33 | 0.4 |
| β[(XEFRKC+Azithromycin)×course of treatment] | -0.02 | -0.21 | 0.16 |
| β[(XEXJZK+Azithromycin)×course of treatment] | 0.05 | -0.12 | 0.2 |
| β[(PDLXY+Azithromycin)×course of disease] | -5.43 | -10.74 | 0.07 |
| β[(SHL+Azithromycin)×course of disease] | -0.33 | -2.89 | 2.09 |
| β[(XEFRKC+Azithromycin)×course of disease] | -0.09 | -0.44 | 0.29 |
| β[(XEXJZK+Azithromycin)×course of disease] | -0.15 | -0.45 | 0.18 |

**Interventions:** PDLXY: Pudilan Xiaoyan oral liquid; SHL: Shuanghuanlian oral liquid; XEFRKC: Xiaoer Feire Kechuan oral liquid; XRXJZK: Xiaoer Xiaoji Zhike oral liquid.

# File 1 The Detailed Search Strategy

**The Pubmed database Search Strategy**

#1: (((((Mycoplasma pneumoniae pneumonia[MeSH Terms]) OR (Mycoplasma pneumoniae pneumonia[Title/Abstract])) OR (Mycoplasma pneumoniae pneumonia in children[Title/Abstract])) OR (Mycoplasma pneumonia in children[Title/Abstract])) OR (Mycoplasma pneumoniae in pediatric patients[Title/Abstract])) OR (Children with Mycoplasma Pneumonia[Title/Abstract])

Results 4549

#2: ((((oral liquid[Title/Abstract]) OR (Pudilan Xiaoyan oral liquid[Title/Abstract])) OR (Shuanghuanglian oral liquid[Title/Abstract])) OR (Xiaoer Feire Kechuan oral liquid[Title/Abstract])) OR (Xiaoer Xiaoji Zhike oral liquid[Title/Abstract])

Results 866

#3: (((((((((randomized controlled study[Title/Abstract]) OR randomized controlled trial[Title/Abstract]) OR randomized trial[Title/Abstract]) OR randomized study[Title/Abstract]) OR randomized placebo-controlled study[Title/Abstract]) OR randomized parallel-group study[Title/Abstract]) OR controlled clinical trial[Title/Abstract]) OR multicenter study[Title/Abstract]) OR double-blinded controlled study[Title/Abstract])

Results 193844

#4：#1 AND #2 AND #3

Results **0**

**The Cochrane library database Search Strategy**

#1: (Mycoplasma pneumoniae pneumonia or Mycoplasma pneumoniae pneumonia in children or Mycoplasma pneumonia in children or Mycoplasma pneumoniae in pediatric patients or Children with Mycoplasma Pneumonia):ti,ab,kw

Results 188

#2: (oral liquid or Pudilan Xiaoyan oral liquid or Shuanghuanglian oral liquid or Xiaoer Fire Kechuan oral liquid or Xiaoer Xiaoji Zhike oral liquid):ti,ab,kw

Results 6367

#3: (randomized controlled study or randomized controlled trial or randomized trial or randomized study or randomized placebo-controlled study or randomized parallel-group study or controlled clinical trial or multicenter study or double-blinded controlled study):ti,ab,kw

Results 896579

#:4：#1 AND #2 AND#3

Results 0

**The Embase database Search Strategy**

#1: 'mycoplasma pneumoniae pneumonia':ab,ti OR 'mycoplasma pneumoniae pneumonia in children':ab,ti OR 'mycoplasma pneumonia in children':ab,ti OR 'mycoplasma pneumoniae in pediatric patients':ab,ti OR 'children with mycoplasma pneumonia':ab,ti

Results 665

#2: 'oral liquid':ab,ti OR 'pudilan xiaoyan oral liquid':ab,ti OR 'shuanghuanglian oral liquid':ab,ti OR 'xiaoer feire kechuan oral liquid':ab,ti OR 'xiaoer xiaoji zhike oral liquid':ab,ti

Results 1356

#3: 'randomized controlled study':ab,ti OR 'andomized controlled trial':ab,ti OR 'randomized trial':ab,ti OR 'randomized study':ab,ti OR 'randomized placebo-controlled study':ab,ti OR 'randomized parallel-group study':ab,ti OR 'controlled clinical trial':ab,ti OR 'multicenter study':ab,ti OR 'double-blinded controlled study':ab,ti

Results 172635

#4: #1 AND #2 AND #3

Results 0

**Results of the references of included studies and reviews and Register the website of clinical trials**

References of included studied Results =0

References of reviews Results =0

Chinese Clinical Trial Registry (http://www.chictr.org.cn) Results =0

# File 2 Reference of Included Studies

Bai, J. X. (2017). Observation on the curative effect of Xiaoerfeirekechuan oral liquid combined with azithromycin in the treatment of Mycoplasma pneumoniae pneumonia. Electronic Journal of Clinical Medical Literature. 4, 18757

Cai, F. G. (2018). Efficacy of Xiaoerfeirekechuan Oral Liquid combined with Azithromycin in the treatment of children with Mycoplasma pneumoniae pneumonia. Health Guide, 379. doi:10.3969/j.issn.1006-6845.2018.17.365

Chao, L. M. and Huang, L. L. (2019). Effect of Xiaoer Xiaoji Zhike Oral Liquid combined with Azithromycin on the level of inflammatory cells in children with mycoplasma pneumonia. Strait Pharmaceutical Journal. 31, 189-191

Chen, J. Q. (2011). Clinical observation on sequential treatment of children's mycoplasma pneumonia by xiaoer feire kechuan oral liquid and azithromycin. Zhejiang Clinical Medical Journal. 13, 896-897. doi:10.3969/j.issn.1008-7664.2011.08.026

Chen, L. L. (2018). The effect of integrate traditional chinese and western medicine on immune function of mycoplasma pneumonia. Chinese Medicine Modern Distance Education of China. 16, 59-61

Ding, X. L. and Hu, T. (2019). To observe the curative effect of Xiaoerfeirekechuan oral liquid combined with azithromycin in the treatment of children with Mycoplasma pneumoniae pneumonia. China Health Care & Nutrition. 29, 297

Dong, J.,Yu, X. Q.,Mao, Y.,Jie.C.H. and Jiang.Z.H. (2020). Effect of azithromycin combined with pediatric feike kechuan oral liquid on mycoplasma pneumonia. China Health Standard Management. 11, 112-114

Fan, S. H. and He, Y. X. (2017). Shuanghuanglian oral liquid (child type) combined with azithromycin in the treatment of Clinical study of mycoplasma pneumonia in children. China Practical Medicine. 12, 114-115

Fang, H. (2019). Evaluation of the effect of Xiaoerfeirekechuan oral liquid combined with azithromycin in the treatment of children with Mycoplasma pneumoniae pneumonia. Contemporary Medical Symposium. 17, 141-142

Gao, J. (2018). Study on the effect of Xiaoerfeirekechuan oral liquid combined with azithromycin in the treatment of children with Mycoplasma pneumoniae pneumonia. Bao Jian Wen Hui, 153. doi:10.3969/j.issn.1671-5217.2018.12.154

Gao, J. and Wu, X. L. (2019). The influence of pudilan combined with western medicine on pulmonary function and inflammation in children with mycoplasma pneumonia. Chinese Journal of Coal Industry Medicine. 22, 307-311

Guo, C. and He, Y. X. (2017). Observation on the effect of Shuanghuanglian oral liquid (child type) combined with azithromycin in the treatment of mycoplasma pneumonia in children. Journal of Practical Traditional Chinese Medicine. 33, 818-819

Guo, Y. B. (2016). Pudilan antiphlogistic oral liquid combined with azithromycin in the treatment of mycoplasma pneumonia in children for 50 cases. Chinese Medicine Modern Distance Education of China. 14, 100-101

Guo, Y. N. (2016). Pudilan combined with azithromycin in the treatment of children with mycoplasma pneumonia clinical efficacy and influence on the causes of cytositis. Journal of China Prescription Drug. 14, 79-80

Han, J. F.,Liu, X. Y. and Wang, H. J. (2016). Effect of azithromycin combined with infantile feirekechuan oral solution for mycoplasma pneumonia in children. Journal of Xinxiang Medical University. 33, 483-484+488

Hu, Y. H. (2018). Observation on the curative effect of azithromycin combined with pediatric antitussive oral solution in the treatment of children's mycoplasma pneumonia. Chinese Community Doctors. 34, 105-106

Huang, Y. K. (2019). Effect of xiaoer xiaoji zhike oral liquid in children with mycoplasma pneumonia. Henan Medical Research. 28, 3401-3402

Jin, Q. Y. (2016). Analysis of the curative effect of Xiaoerfeirekechuan oral liquid combined with azithromycin in the treatment of children with Mycoplasma pneumoniae pneumonia. Chinese Journal of Modern Drug Application. 10, 190-191

Li, J. (2019). Effect of infantile feire kechuan oral solution combined with azithromycin in treatment of mycoplasma pneumonia in children. Infectious Disease Information. 32, 151-153

Li, X. H. (2018). Observation on the effect of azithromycin combined with Xiaoer Xiaoji Zhike Oral Liquid in the treatment of mycoplasma pneumonia in children. China Rural Health, 44+43

Li, Y. F. and Jin, Y. X. (2018). Observation of the curative effect of azithromycin combined with Xiaoer Xiaoji Zhike oral liquid in the treatment of mycoplasma pneumonia. Journal of North Pharmacy. 15, 38-39

Lin, H. R.,Wan, N. J.,Qian, Y.,Zhang, Y. M. and Zhang, Q. (2019). Effects of xiaoer feire kechuan oral liquid combined with azithromycin on pulmonary function and inflammatory factors in children with mycoplasma pneumonia. Progress in Modern Biomedicine. 19, 3267-3270+3217

Liu, H. (2019). Clinical trial of azithromycin sequential therapy combined with Shuanghuanglian

oral liquid on infantile mycoplasma pneumonia. The Chinese Journal of Clinical Pharmacology. 35, 1996-1998

Liu, J. (2017). Analysis of the curative effect of Xiaoerfeirekechuan oral liquid combined with azithromycin in the treatment of mycoplasmal pneumonia. Nei Mongol Journal of Traditional Chinese Medicine. 36, 77-78

Liu, N.,Chen, X. D. and Yin, L. H. (2016). Clinical effect of azithromycin sequential therapy combined with shuanghuanglian oral solution on pediatric mycoplasma pneumonia and the impact on serum inflammatory cytokines levels and immunologic function. Practical Journal of Cardiac Cerebral Pneumal and Vascular Disease. 24, 94-97

Liu, Y. L. (2018). Clinical effect observation of Shuanghuang Lian oral liquid(only for children) on Mycoplasma Pneumonia in Children. Clinical Journal of Traditional Chinese Medicine. 30, 300-303

Liu, Y. M. and Cao, Z. Y. (2017). Analysis of the curative effect of Xiaoerfeirekechuan oral liquid combined with azithromycin in the treatment of children with Mycoplasma pneumoniae pneumonia. World Latest Medicine Information. 17, 115

Luan, F.,Man, L. N.,Wang, K.,Lv, X. Y.,Li, X. and Liu, Y. C. (2020). Effect of Xiaoerfeirekechuan Oral Liquid and Azithromycin on Mycoplasma Pneumoniae Pneumonia. Chinese Journal of Modern Drug Application. 14, 133-135

Luo, Y. T.,Kong, X. D.,Lin, Z. Z.,Zeng, Z. M.,Zhang, Y. Z. and Chen, J. M. (2017). Efficacy of Xiaoer-feire-kechuan oral solution in the treatment of Mycoplasma pneumoniae infection and its effect on serum inflammatory factors in children. Hainan Medical Journal. 28, 589-591

Ma, G. Q.,Cao, H. Q. and Liu, J. L. (2018). Observation on therapeutic effect of oral liquid of cough and asthma for children with lung heat combined with antibiotics on mycoplasma pneumonia in children. Chinese Youjiang Medical Journal. 46, 701-705

Mei, J. H. (2016). Clinical observation on the treatment of 45 children with mycoplasma pneumonia pneumonia by azithromycin sequential therapy and pediatric feire kechuan oral liquid. Chinese Remedies & Clinics. 16, 1197-1198

Meng, R. R. (2016). Observation on the effect of xiao'er feire kechuan oral solution with azithromyci on mycoplasma pneumoniae pneumonia. World Chinese Medicine. 11, 1517-1519+1523

Pang, X. L. and Guo, H. L. (2007). 130 cases of children's mycoplasma pneumonia treated by xiaoerfeire kechuan oral liquid combined with azithromycin. Shaanxi Journal of Traditional Chinese Medicine, 777-778

Shi, J. Y. (2020). Observation on the effect of azithromycin combined with Shuanghuanglian oral liquid in the treatment of mycoplasma pneumonia in children. Diet Health. 7, 47

Song, L. J. (2019). Observation on the curative effect of Xiaoerfeirekechuan oral liquid combined with azithromycin in the treatment of mycoplasma pneumonia in children. Journals of Frontiers of Medicine. 9, 134-135

Tan, Z. B. (2019). Xiaoer feire kechuan oral liquid and azithromycin in the treatment of children with mycoplasma pneumoniae pneumonia. China Continuing Medical Education. 11, 138-141

Wang, D. (2016). Treatment of mycoplasma in children with azithromycin and xiaoer xiaoji zhike oral liquid clinical efficacy of pneumonia. Clinical Research and Practice. 1, 91

Wang, H. J. (2018). Efficacy of Shuanghuanglian Oral Liquid Only for Children Combined with Azithromycin in the Treatment of Mycoplasma Pneumonia in Children. Chinese and Foreign Medical Research. 16, 25-27

Wang, M.,Zhang, X. Q.,Yang, J.,W.J., L. and Lin, W. J. (2018). Effect of sequential therapy with azithromycin combined shuanghuanglian oral liquid on the T lymphocyte subsets, serum inflammatory cytokines levels of children with mycoplasma pneumonia. Hebei Medicine. 24, 1013-1017

Wang, X. D. (2019). Analysis of the clinical effect and short-term recurrence of infantile mycoplasma pneumonia treated with pediatric feire kechuan oral liquid combined with azithromycin. Chinese Community Doctors. 35, 106-107

Wang, X. F. (2019). Evaluation of the effect of Xiaoerfeirekechuan oral liquid combined with azithromycin in the treatment of children with mycoplasma pneumoniae pneumonia. Renowned Doctor, 266

Wang, Y. P. and Wang, X. L. (2019). Analysis of the clinical effectiveness of Pudilan combined with azithromycin in the treatment of mycoplasma pneumonia in children. Shanxi Medical Journal. 48, 1711-1713

Wang, Y. R.,Zhang, Y.,Zhou, Q.,Wang, W. L.,Wang, J. and Zhang, Q. (2019). Effect of xiaoer xiaoji zhike oral liquid combined with azithromycin onmycoplasma immune function in children with mycoplasma pneumonia. Chinese Archives of Traditional Chinese Medicine. 37, 1168-1171

Xia, J. X.,Lu, Q. F. and Shao, J. (2015). Clinical study of Pudilan combined with azithromycin in the treatment of mycoplasma pneumonia in children. China Higher Medical Education, 137+140

Xie, Y. K. (2015). Xiaoerxiaojizhike Oral Liquid Combined with Azithromycin for Treating Mycoplasma

Pneumonia in 60 Cases. China Pharmaceuticals. 24, 100-101

Xiong, H. Q. (2018). Observation on the curative effect of Xiaoerfeirekechuan oral liquid combined with azithromycin in the treatment of Mycoplasma pneumoniae pneumonia. Cardiovascular Disease Electronic Journal of Integrated Traditional Chinese and Western Medicine. 6, 197-198

Xu, Y. N. (2016). Xiaoer feire kechuan oral liquid and azithromycin injection in the treatment of pediatric mycoplasma pneumoniae infection for 25 cases. Chinese Medicine Modern Distance Education of China. 14, 102-103

Xue, Y. (2020). Effect of Xiaoer Xiaoji Zhike Oral Liquid and Azithromycin on Symptom Improvement and Immune Function in Children with Mycoplasma Pneumonia. Medical Journal of Chinese People's Health. 32, 75-77

Yang, L. X. (2018). Clinical study of azithromycin combined with Xiaoerfeirekechuan oral liquid in the treatment of children with mycoplasma pneumonia. Cardiovascular Disease Electronic Journal of Integrated Traditional Chinese and Western Medicine. 6, 172-173

Yang, X.,Jiao, R. and Zhang, C. (2020). Therapeutic effects of children feire kechuan oral liquid combined with antibiotics on child patients with mycoplasma pneumonia. China Journal of Pharmaceutical Economics. 15, 111-114

Yang, Y. (2019). Observation on the curative effect of azithromycin combined with Xiaoer Xiaoji Zhike Oral Liquid in the treatment of mycoplasma pneumonia. Health Guide, 216

Yao, Y. X. (2018). Effective observation on treating mycoplasma pneumonia with the Shuanghuanglian oral liquid (only for children). Clinical Journal of Chinese Medicine. 10, 29-31

Ye, P. F. and Chen, Q. F. (2018). Clinical efficacy and safety analysis of azithromycin combined with Xiaoer Xiaoji Zhike Oral Liquid in the treatment of mycoplasma pneumonia in children. For All Health. 12, 172-173. doi:10.3969/j.issn.1009-6019(x).2018.02.226

Yu, Q. and Yuan.H.Y. (2016). Xiaoer feirekechuan oral solution combined with azithromycin for treating pediatric Mycoplasmal pneumonia in 43 cases. China Pharmaceuticals. 25, 121-122

Yu, W. (2017). Clinical analysis of azithromycin combined with Xiaoer Xiaoji Zhike Oral Liquid in the treatment of mycoplasma pneumonia in children. Electronic Journal of Clinical Medical Literature. 4, 5704

Yuan, L. F. and Wang, Q. L. (2015). Observation on the curative effect of azithromycin combined with Xiaoer Xiaoji Zhike Oral Liquid in the treatment of 43 children with mycoplasma pneumonia. Journal of Pediatrics of Traditional Chinese Medicine. 11, 40-42

Zhang, C.,Chen, Z. G. and Liu, Y. (2015). Forty-Four Cases of Children's Mycoplasma Pneumonia Treated with Oral Pu Di Lan Solution in Combination with Azithromycin. Henan Traditional Chinese Medicine. 35, 2531-2533

Zhang, W. J. (2017). Clinical observation of shuanghuanglian oral liquid (children's type) combined with azithromycin sequential therapy for treatment of mycoplasma pneumonia in children. World Latest Medicine Information. 17, 174-175+178

Zhao, X. L. (2019). Analysis of the curative effect of azithromycin combined with Xiaoer Xiaoji Zhike Oral Liquid in the treatment of mycoplasma pneumonia in children. Journal of Medical Forum. 40, 143-144

Zheng, F. F.,Jiang, D.,Mai, L. and Zhou, C. X. (2018). Curative effect of xiaoji zhike oral solution combined with azithromycin in treatment of mycoplasmal pneumonia children and effect on the T-lymphocyte subset level. Systems Medicine. 3, 1-4

Zhu, G. R. (2019). Clinical observation of azithromycin combined with Xiaoerfeirekechuan oral liquid in the treatment of mycoplasma pneumonia in children. Clinical Medicine. 39, 94-96

Zhu, M.,Chen, G. M. and Xu, S. J. (2017). Observation on the curative effect of azithromycin in treatment of mycoplasma pneumonia in children with infantile Feirekechuan oral liquid. Jilin Medical Journal. 38, 441-442

Zong, D. L. (2019). Effect of Azithromycin Combined with Xiaoer Xiaoji Zhike Oral Liquid in Treating Mycoplasma Pneumonia in Children. China Health Vision, 73. doi:10.3969/j.issn.1005-0019.2019.09.116

# PRISMA Checklist

| **Section and Topic** | **Item #** | **Checklist item** | **Location where item is reported** |
| --- | --- | --- | --- |
| **TITLE** | | |  |
| Title | 1 | Identify the report as a systematic review. | 1 |
| **ABSTRACT** | | |  |
| Abstract | 2 | See the PRISMA 2020 for Abstracts checklist. | 2 |
| **INTRODUCTION** | | |  |
| Rationale | 3 | Describe the rationale for the review in the context of existing knowledge. | 4 |
| Objectives | 4 | Provide an explicit statement of the objective(s) or question(s) the review addresses. | 4 |
| **METHODS** | | |  |
| Eligibility criteria | 5 | Specify the inclusion and exclusion criteria for the review and how studies were grouped for the syntheses. | 5 |
| Information sources | 6 | Specify all databases, registers, websites, organisations, reference lists and other sources searched or consulted to identify studies. Specify the date when each source was last searched or consulted. | 5 |
| Search strategy | 7 | Present the full search strategies for all databases, registers and websites, including any filters and limits used. | 7 |
| Selection process | 8 | Specify the methods used to decide whether a study met the inclusion criteria of the review, including how many reviewers screened each record and each report retrieved, whether they worked independently, and if applicable, details of automation tools used in the process. | 8 |
| Data collection process | 9 | Specify the methods used to collect data from reports, including how many reviewers collected data from each report, whether they worked independently, any processes for obtaining or confirming data from study investigators, and if applicable, details of automation tools used in the process. | 8 |
| Data items | 10a | List and define all outcomes for which data were sought. Specify whether all results that were compatible with each outcome domain in each study were sought (e.g. for all measures, time points, analyses), and if not, the methods used to decide which results to collect. | 8 |
|  | 10b | List and define all other variables for which data were sought (e.g. participant and intervention characteristics, funding sources). Describe any assumptions made about any missing or unclear information. | 8 |
| Study risk of bias assessment | 11 | Specify the methods used to assess risk of bias in the included studies, including details of the tool(s) used, how many reviewers assessed each study and whether they worked independently, and if applicable, details of automation tools used in the process. | 8 |
| Effect measures | 12 | Specify for each outcome the effect measure(s) (e.g. risk ratio, mean difference) used in the synthesis or presentation of results. | 8 |
| Synthesis methods | 13a | Describe the processes used to decide which studies were eligible for each synthesis (e.g. tabulating the study intervention characteristics and comparing against the planned groups for each synthesis (item #5)). | 9 |
|  | 13b | Describe any methods required to prepare the data for presentation or synthesis, such as handling of missing summary statistics, or data conversions. | 9 |
|  | 13c | Describe any methods used to tabulate or visually display results of individual studies and syntheses. | 9 |
|  | 13d | Describe any methods used to synthesize results and provide a rationale for the choice(s). If meta-analysis was performed, describe the model(s), method(s) to identify the presence and extent of statistical heterogeneity, and software package(s) used. | 9 |
|  | 13e | Describe any methods used to explore possible causes of heterogeneity among study results (e.g. subgroup analysis, meta-regression). | 9 |
|  | 13f | Describe any sensitivity analyses conducted to assess robustness of the synthesized results. | 9 |
| Reporting bias assessment | 14 | Describe any methods used to assess risk of bias due to missing results in a synthesis (arising from reporting biases). | 8 |
| Certainty assessment | 15 | Describe any methods used to assess certainty (or confidence) in the body of evidence for an outcome. | 9 |
| **RESULTS** | | |  |
| Study selection | 16a | Describe the results of the search and selection process, from the number of records identified in the search to the number of studies included in the review, ideally using a flow diagram. | 10 |
|  | 16b | Cite studies that might appear to meet the inclusion criteria, but which were excluded, and explain why they were excluded. | 10 |
| Study characteristics | 17 | Cite each included study and present its characteristics. | 10 |
| Risk of bias in studies | 18 | Present assessments of risk of bias for each included study. | 10 |
| Results of individual studies | 19 | For all outcomes, present, for each study: (a) summary statistics for each group (where appropriate) and (b) an effect estimate and its precision (e.g. confidence/credible interval), ideally using structured tables or plots. | 10 |
| Results of syntheses | 20a | For each synthesis, briefly summarise the characteristics and risk of bias among contributing studies. | 10 |
|  | 20b | Present results of all statistical syntheses conducted. If meta-analysis was done, present for each the summary estimate and its precision (e.g. confidence/credible interval) and measures of statistical heterogeneity. If comparing groups, describe the direction of the effect. | 15 |
|  | 20c | Present results of all investigations of possible causes of heterogeneity among study results. | 15 |
|  | 20d | Present results of all sensitivity analyses conducted to assess the robustness of the synthesized results. |  |
| Reporting biases | 21 | Present assessments of risk of bias due to missing results (arising from reporting biases) for each synthesis assessed. | 15 |
| Certainty of evidence | 22 | Present assessments of certainty (or confidence) in the body of evidence for each outcome assessed. | 11 |
| **DISCUSSION** | | |  |
| Discussion | 23a | Provide a general interpretation of the results in the context of other evidence. | 16 |
|  | 23b | Discuss any limitations of the evidence included in the review. | 18 |
|  | 23c | Discuss any limitations of the review processes used. | 18 |
|  | 23d | Discuss implications of the results for practice, policy, and future research. | 18 |
| **OTHER INFORMATION** | | |  |
| Registration and protocol | 24a | Provide registration information for the review, including register name and registration number, or state that the review was not registered. | 5 |
|  | 24b | Indicate where the review protocol can be accessed, or state that a protocol was not prepared. | 5 |
|  | 24c | Describe and explain any amendments to information provided at registration or in the protocol. | 5 |
| Support | 25 | Describe sources of financial or non-financial support for the review, and the role of the funders or sponsors in the review. | 20 |
| Competing interests | 26 | Declare any competing interests of review authors. | 20 |
| Availability of data, code and other materials | 27 | Report which of the following are publicly available and where they can be found: template data collection forms; data extracted from included studies; data used for all analyses; analytic code; any other materials used in the review. | 20 |

*From:*  Page MJ, McKenzie JE, Bossuyt PM, Boutron I, Hoffmann TC, Mulrow CD, et al. The PRISMA 2020 statement: an updated guideline for reporting systematic reviews. BMJ 2021;372:n71. doi: 10.1136/bmj.n71

For more information, visit: <http://www.prisma-statement.org/>
